# SPOP-mediated ZMYND8 ubiquitination and phase separation exclusion drives mTOR inhibitor resistance in kidney cancer

**DOI:** 10.1038/s41467-026-77043-9

**Published:** 2026-08-25

**Authors:** Bo Tang, Rui Sun, Jingwei Shao, Qi-You Wu, Yuqian Yan, Dejie Wang, Zhixian Yu, Lianxin Hu, Yicheng Chen, Chengheng Liao, Qiang Wei, Yige Bao, Haojie Huang

**Affiliations:** 1 Department of Urology, Institute of Urology, West China Hospital, Sichuan University, Chengdu, China; 2 Department of Biochemistry and Molecular Biology, Mayo Clinic College of Medicine and Science, Rochester, MN USA; 3 Department of Urology, Mayo Clinic College of Medicine and Science, Rochester, MN USA; 4 Department of Urology, Institute of Urologic Science and Technology, the First Affiliated Hospital of Zhejiang University School of Medicine, Hangzhou, China; 5 The First Affiliated Hospital of Wenzhou Medical University, Wenzhou, China; 6 Department of Urology, Sir Run Run Shaw Hospital, School of Medicine, Zhejiang University, Hangzhou, China; 7 Department of Pathology, University of Texas Southwestern Medical Center, Dallas, TX USA

**Keywords:** Renal cell carcinoma, Ubiquitylation

## Abstract

mTOR inhibitors including everolimus and temsirolimus have been approved by US FDA for treatment of clear cell renal cell carcinoma (ccRCC) in clinic. However, resistance to these drugs has been inevitable and the underlying mechanism remains poorly understood. Histone modifier ZMYND8 paradoxically acts as a transcription coactivator or corepressor. Here we show that SPOP, a CULLIN3-RING E3 ubiquitin ligase (CRL) substrate-binding protein that is often overexpressed in ccRCC in patients, promotes K63-linked polyubiquitination of ZMYND8 at lysine 398, which inhibits ZMYND8 to form phase separation compartments and drives the formation of ZMYND8-ZHX2 transactivation complex, resulting in aberrant *NEK7* kinase gene transcription, alternative activation of p70S6K, and mTOR inhibitor-resistant cell growth. Inhibition of either SPOP or NEK7 increases ccRCC cell sensitivity to mTOR inhibitor. Treatment with a NEK7 proteolysis-targeting chimera (PROTAC) effectively inhibits aberrant p70S6K activation and overcame everolimus resistance in ccRCC cells in vitro and in mice. Our findings uncover a function switch of ZMYND8 driven by overexpressed SPOP as a key mechanism that causes mTOR inhibitor resistance and nominate NEK7 as a potential target of thwarting mTOR inhibitor resistance in ccRCC.

## Abstract (web summary)

Resistance to mTOR inhibitors is common in clear cell renal cell carcinoma (ccRCC) patients. The authors show that the histone reader ZMYND8 undergoes ubiquitination, disrupting its phase separation and promoting its recruitment into a transcriptional activator complex with ZHX2, which leads to mTOR-independent activation of p70S6K and drug resistance in preclinical ccRCC models

## Introduction

Renal cell carcinoma is a common malignancy, with an estimated 434, 840 incident cases worldwide in 2022^1^. Clear cell renal cell carcinoma (ccRCC) is the most common histologic subtype, accounting for 75–80% of all cases^1^. In advanced or metastatic RCC, first-line treatment typically involves combining immune checkpoint inhibitors with tyrosine kinase inhibitors, while second-line treatments often use mTOR or HIF2α inhibitors^2,3^. Resistance to mTOR inhibitors is widespread in ccRCC patients through multiple mechanisms. Tumor cells often activate alternative signaling cascades, such as the PI3K/AKT or MAPK/ERK pathways, to bypass mTOR blockade. In addition, inhibition of mTOR may relieve negative feedback regulation, leading to compensatory activation of upstream kinases like AKT. Genetic or epigenetic alterations in pathway components, as well as transcriptional reprogramming, can further diminish drug sensitivity. Moreover, cancer cells may adapt by rewiring their metabolism or exploiting protective cues from the tumor microenvironment, together contributing to reduced efficacy of mTOR-targeted therapies. Drug resistance continues to be a significant challenge for patients with advanced ccRCC. The median survival time for patients with advanced renal cancer is approximately four years^1^. Addressing drug resistance remains a crucial focus in the treatment of renal cancer.

The bromodomain protein zinc finger MYND-type containing 8 (ZMYND8, also termed RACK7 and PRCBP1) serves as a reader of the dual histone mark H3K4me1/H3K14ac^4^. Increasing evidence suggests that ZMYND8 plays an important role in cancer pathogenesis, although its function varies depending on the context. ZMYND8 recruits lysine demethylase 5C (KDM5C) histone demethylase to restrict enhancer overactivation and suppress tumorigenesis^5^. It can also recruit KDM5D and antagonize metastasis-linked gene expression^4^. In contrast to its tumor-suppressing roles, ZMYND8 can promote tumorigenesis by recruiting bromodomain-containing protein 4 (BRD4) or enhancer of zeste homolog 2 (EZH2) to positively regulate gene expression^6,7^. Thus, ZMYND8 paradoxically acts as both a transcriptional coactivator or corepressor although the underlying mechanisms remain unclear.

Speckle-type POZ protein (SPOP) is the substrate-recognition subunit of the CULLIN-3 (CUL3)–RING-box E3 ubiquitin ligase (CRL) complex, where it mediates the ubiquitination of target proteins through RBX1-dependent recruitment of the E2 ubiquitin-conjugating enzyme. In prostate cancer, SPOP functions as a tumor suppressor by promoting the proteasomal degradation of several cancer-related proteins, including androgen receptor (AR), ETS-related gene (ERG) and BRD4 in the nucleus^8–10^. Intriguingly, in kidney cancer, SPOP is often overexpressed in ccRCC and functions as an oncoprotein by inducing degradation of tumor suppressor proteins such as PTEN, SETD2, and LATS1 in the cytoplasm of ccRCC cells^11–13^. Given the context-dependent roles of SPOP in human malignancies, further investigation into the molecular mechanisms by which SPOP drives oncogenesis in different cancer types is imperative^14^.

Here, we show that SPOP overexpression induces K63-linked ubiquitination of ZMYND8 in human ccRCC cell lines. The ubiquitination of ZMYND8 appears to function as a molecular switch, altering its regulatory roles in distinct cellular contexts. Rather than facilitating phase separation, this modification redirects ZMYND8 toward assembling a transcriptional activator complex with zinc fingers and homeoboxes 2 (ZHX2). Through this interaction, ZMYND8 gains the capacity to promote transcriptional upregulation of *NEK7* gene, which in turn contributes to the alternative activation of p70S6K signaling. This cascade ultimately provides a mechanistic basis for the emergence of resistance to mTOR inhibitors in ccRCC.

## Results

### SPOP overexpression promotes mTOR inhibitor resistance in ccRCC via K63-linked polyubiquitination of ZMYND8 at lysine-398

**Fig. 1:**
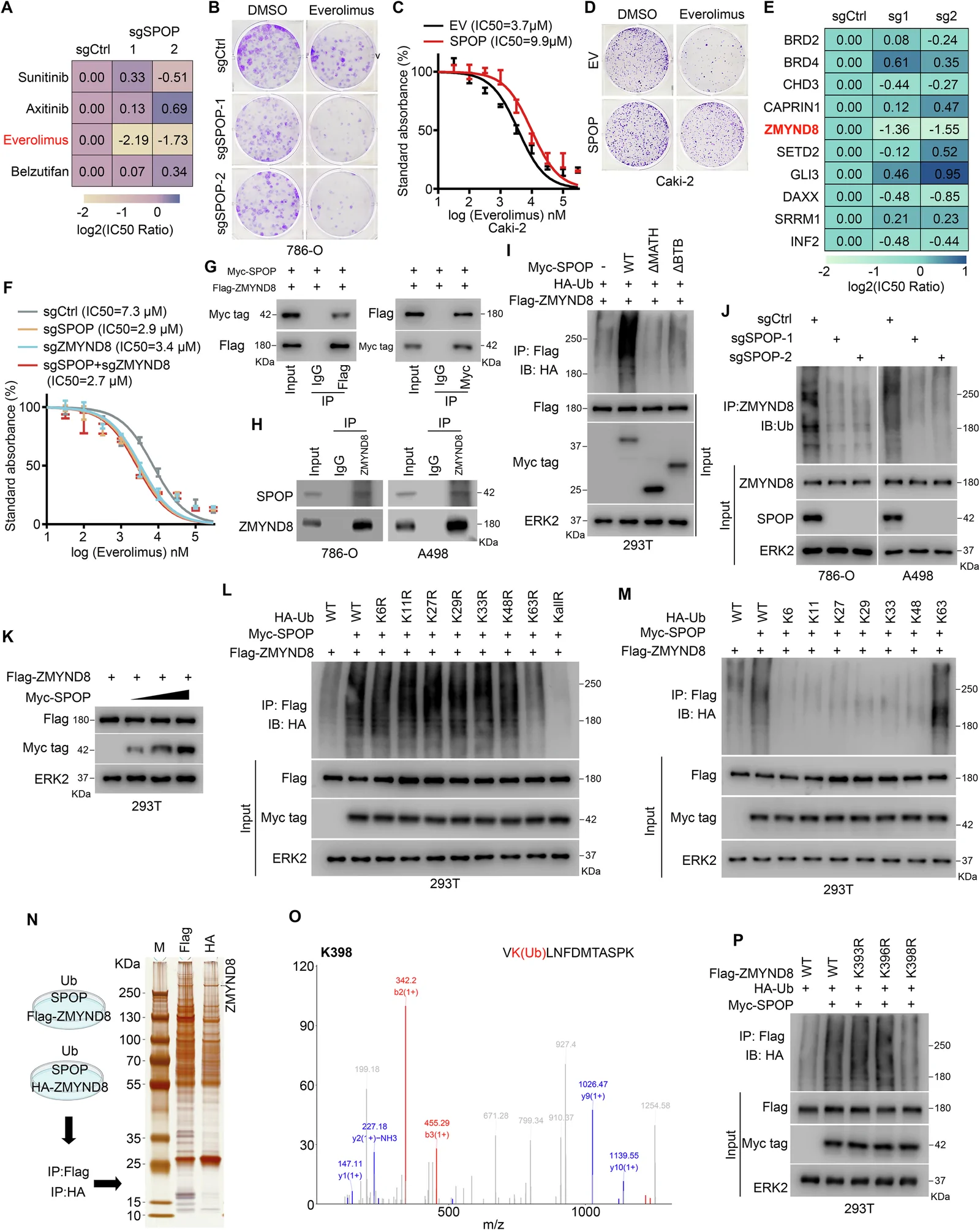
SPOP promotes resistance to mTOR inhibitors in ccRCC via K63-linked polyubiquitination of ZMYND8 at Lysine-398. **A** 786-O cells infected with lentivirus for sgCtrl or sgSPOP were treated with different drugs, and cell viability was measured by MTS assay. Heatmap showing the normalized IC50 ratio (log2[IC50ratio]). **B** Colony formation assay of sgCtrl or sgSPOP 786-O cells treated with DMSO or everolimus (5 μM, 24 h), followed by photograph. **C** IC50 of everolimus in control or SPOP overexpressing Caki-2 cells treated with everolimus for 72 h. **D** Colony formation assay of control or SPOP-overexpressing Caki-2 cells treated with DMSO or everolimus (5 μM, 24 h) followed by photograph. **E** 786-O cells infected with lentivirus for indicated sgRNAs were treated with everolimus (5 μM, 24 h), and cell viability was measured by MTS assay. Heatmap showing the normalized IC50 ratio (log2[IC50ratio]). **F** 72 h IC50 of everolimus in 786-O cells transfected with indicated sgRNAs. **G** Western blot (WB) analysis of whole-cell lysate (WCL) and co-IP samples of immunoglobulin G (IgG) or indicated antibody in 293 T cells transfected with indicated plasmids. **H** WB analysis of co-IP samples of IgG or anti-ZMYND8 antibody in 786-O or A498 cells. **I** WB analysis of WCL and co-IP samples in 293 T cells transfected with indicated plasmids. ERK2 was used as a loading control. **J** WB analysis of indicated proteins in 786-O and A498 cells stably expressing control (sgCtrl) or SPOP-specific sgRNAs. **K** WB of indicated proteins in 293 T cells transfected with indicated plasmids. **L**, **M** WB analysis of indicated proteins in 293 T cells transfected with WT ubiquitin or K-to-R mutant (**L**) or WT ubiquitin or specific-lysine-residue-only ubiquitin mutant (**M**). **N** Schematic of the strategy for analyzing ZMYND8 ubiquitination sites. **O** Identification and quantification of ZMYND8 K398 ubiquitination. **P** WB analysis of indicated proteins in WCL and co-IP samples from 293 T cells transfected with indicated plasmids.WB analyses (**G**–**M**, **P**) are representative of three independent experiments with similar results. MTS assays (**A**, **C**, **E**, **F**), colony formation assays (**B**, **D**) are obtained in three biological replicates. Data are shown as mean ± SD (**C**, **F**). Source data are provided as a Source Data file.

Considering the important regulatory role of SPOP as an oncogene in ccRCC^13^, we first investigated the effect of SPOP on the efficacy of ccRCC drug treatment. 786-O is a typical ccRCC cell line with high expression of SPOP (Supplementary Fig. 1A). SPOP was knocked out (KO) in 786-O cells (Supplementary Fig. 1B), followed by treatment with several drugs in clinical use for ccRCC. Drug sensitivity (IC_50_) analysis with MTS assay showed that SPOP KO largely sensitized 786-O cells to the mTOR inhibitor everolimus, but not other three drugs tested (Fig. 1A and Supplementary Fig. 1C). The mTOR signaling pathway is frequently dysregulated in ccRCC, and is associated with aggressive behavior and poor prognosis in ccRCC patient^15^. FDA-approved mTOR inhibitors everolimus and temsirolimus are now being used for second-line treatment of ccRCC^3,16^. Colony formation assay also showed that everolimus sensitivity was increased in SPOP KO 786-O cells (Fig. 1B and Supplementary Fig. 1D). Conversely, exogenous expression of SPOP in SPOP-low Caki-2 cells (Supplementary Fig. 1A, E) reduced everolimus sensitivity (Fig. 1C, D and Supplementary Fig. 1F). Combined treatment of everolimus and SPOP-IN-6b, a SPOP-specific inhibitor^17^, significantly enhanced inhibition on cell proliferation compared with each single agent alone (Supplementary Fig. 1G–I). These data suggest that SPOP overexpression decreases mTOR inhibitor sensitivity in ccRCC cells.

As an E3 ubiquitin ligase, SPOP usually regulates specific substrates through ubiquitination, leading to changes in downstream signaling pathways^14^. To understand how SPOP regulates ccRCC resistance to mTOR inhibitors, we re-analyzed the results of the yeast two-hybrid screen we previously performed using the full-length SPOP as bait^8^. We focused on the top ten interacting proteins of SPOP (Supplementary Fig. 1J) by knocking out them individually in 786-O cells and treated the cells with everolimus. We found that knockout of ZMYND8 but not other nine top hits largely increased everolimus sensitivity in 786-O (Fig. 1E and Supplementary Fig. 1K). We further showed that knockout of SPOP or ZMYND8 alone increased everolimus sensitivity, but their co-knockout failed to further improve the sensitivity (Fig. 1F), indicating that SPOP and ZMYND8 may regulate mTOR inhibitor sensitivity in 786-O ccRCC cells through the same pathway.

We next determined whether SPOP regulates ZMYND8 function as an E3 ubiquitin ligase. Co-immunoprecipitation (co-IP) assays confirmed that ectopically expressed SPOP and ZMYND8 interact with each other in 293 T cells (Fig. 1G). Their interaction also occurred in the endogenous level in 786-O and A498 cells, two SPOP-high expression ccRCC cell lines^13^ (Fig. 1H and Supplementary Fig. 1A). These results indicate that SPOP interacts with ZMYND8 under physio- and/or pathological conditions.

SPOP is a structurally well-characterized BTB protein that contains a substrate-binding MATH domain at the N terminus and an internal CUL3-binding BTB domain^18,19^. Deletion of the MATH domain (ΔMATH), but not the BTB domain (ΔBTB), abolished the interaction between SPOP and ZMYND8 (Supplementary Fig. 2A, B). Several common mutations on SPOP MATH domain, such as F102C and F133V, can also significantly diminish the interaction between SPOP and ZMYND8 (Supplementary Fig. 2C). Only wild-type (WT) SPOP, but not ΔMATH or enzymatically dead mutant (ΔBTB), was able to induce ZMYND8 ubiquitination (Fig. 1I). We also demonstrated that the enzymatic activity is required for SPOP-mediated ZMYND8 polyubiquitination in vitro and in vivo (Supplementary Fig. 2D, E). Specific substrate-binding consensus (SBC) motifs (Φ-π-S/T-S/T-S/T, where Φ is a nonpolar residue and π is a polar residue) have been identified in known SPOP substrates, such as AR, ERG and BRD4^8–10^. We identified three putative SBC motifs (puSBC1-3) in ZMYND8, including ^648^LKSTS^652^, ^819^WNSSS^823^, and ^1088^GSSSS^1092^ (Supplementary Fig. 2F). Analysis with deletion of each puSBC motif individually revealed that only the C-terminal ^1088^GSSSS^1092^ motif was critical for SPOP binding, as its deletion completely abolished SPOP-ZMYND8 interaction (Supplementary Fig. 2G).

To investigate whether SPOP causes ZMYND8 degradation, we knocked out endogenous SPOP in 786-O and A498 cells. As expected, we observed a decrease in ZMYND8 ubiquitination level, but to our surprise, neither ZMYND8 mRNA nor protein expression was affected by SPOP KO, and similar results were obtained for the half-life of ZMYND8 protein (Fig. 1J and Supplementary Fig. 2H–J). Similarly, treatment with the SPOP inhibitor SPOP-IN-6b abolished SPOP overexpression-induced ZMYND8 polyubiquitination but had no effect on protein expression (Supplementary Fig. 2K). Moreover, ectopically expressed SPOP had little or no effect on ZMYND8 protein level in 293 T cells (Fig. 1K). These data indicate that SPOP promotes non-degradable polyubiquitination of ZMYND8.

To determine the type of the polyubiquitination chain on ZMYND8 caused by SPOP, we mutated seven lysine residues in ubiquitin protein to arginine, either individually or together. Only K63R mutation abolished SPOP-induced ZMYND8 polyubiquitination (Fig. 1L). Ubiquitination assay using a K63-only ubiquitin, in which only the K63 residue remains unmutated, further confirmed that SPOP catalyzes K63-linked polyubiquitination of ZMYND8 (Fig. 1M).

To identify the ubiquitinated site(s) on ZMYND8 in SPOP-overexpressing cells, we immunoprecipitated Flag-ZMYND8 and HA-ZMYND8 proteins for LC-MS/MS analysis (Fig. 1N). Three potential ubiquitinated lysine sites, including K393, K396, and K398, were identified (Fig. 1O and Supplementary Fig. 2L, M). We mutated the three lysine residues into arginine separately (K393R, K396R, and K398R) and found that only K398R reduced ZMYND8 ubiquitination level (Fig. 1P), suggesting that K398 is the major site in ZMYND8 protein ubiquitinated by SPOP. These results suggest that SPOP mediates non-degradable K63-linked polyubiquitination of ZMYND8 primarily on K398 residue.

### SPOP inhibits ZMYND8 to form liquid compartments

**Fig. 2:**
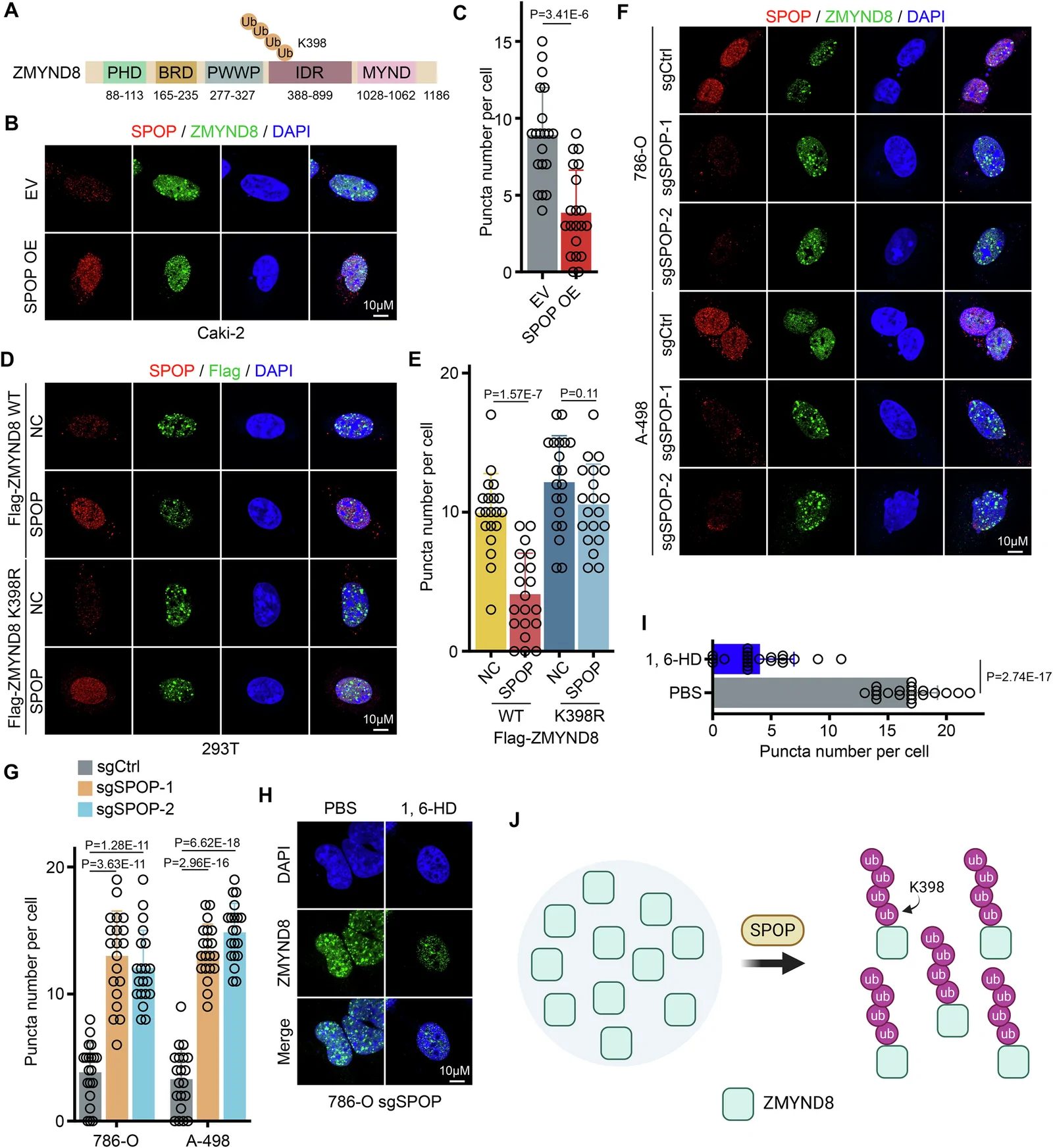
SPOP inhibits ZMYND8 forming liquid compartments. **A** ZMYND8 domain architecture. **B** Immunofluorescent cytochemistry (IFC) of SPOP and ZMYND8 in control and SPOP-overexpressed (OE) Caki-2 cells. **C** Quantification of ZMYND8 puncta number in cells from (**B**). *P* = 3.41E-6. **D** Immunofluorescent cytochemistry (IFC) of SPOP and Flag in Caki-2 ZMYND8-KO cells transfected with indicated plasmids. **E** Quantification of Flag puncta number in cells from (**D**). *P* values arranged in order*:*1.57E-7, 0.11. **F** Immunofluorescent cytochemistry (IFC) of SPOP and ZMYND8 in mock and SPOP KO 786-O and A-498 cells infected with lentivirus expressing the indicated sgRNAs. **G** Quantification of ZMYND8 puncta number in cells from (**F**). *P* values arranged in order*:*3.63E-11, 1.28E-11, 2.96E-16, 6.62E-18. **H** Immunofluorescent cytochemistry (IFC) of 786-O SPOP KO cells treated with DMSO or 1,6-hexanediol. **I** Quantification of ZMYND8 puncta number in cells from (**H**). *P* = 2.74E-17. **J** A hypothetical model depicting the inhibition of the ZMYND8 liquid compartment formation by SPOP overexpression and ZMYND8 K398 ubiquitination (Created in BioRender. Qiu, L. (2026) https://BioRender.com/gg20smz). IFC Data are shown as the mean ± SD from 20 randomly selected fields from three biological replicates (**B**, **D**, **F**, **H**). Statistics: Two-tailed unpaired Student’s *t*-test are used in (**C**, **E**, **G**, and **I**). Source data are provided as a Source Data file.

ZMYND8 can form phase-separated liquid compartments and its intrinsically disordered region (IDR) is required for liquid–liquid phase separation (LLPS)^20^. In vitro analysis confirmed that the liquid droplets of ZMYND8 were sensitive to 1,6-hexanediol (Supplementary Fig. 3A), a potent inhibitor of LLPS driven by hydrophobic interactions^21^ and their fluorescence signal exhibited fast recovery after photo bleaching (Supplementary Fig. 3B). We then evaluated the spheric protein puncta of ZMYND8 in SPOP-low Caki-2 cells (Supplementary Fig. 1A) and found that ZMYND8 is primarily localized in the nucleus, where it forms protein puncta. Treatment with 1,6-hexanediol significantly reduced the number of these puncta (Supplementary Fig. 3C, D). Given the previous report that ubiquitination can affect LLPS^22^ and our observation that K398 on ZMYND8 is located within the IDR domain (Fig. 2A), we hypothesized that ubiquitination at K398 might influence ZMYND8 phase separation. To this end, we overexpressed SPOP in Caki-2 cells and observed that SPOP overexpression largely decreased the number of ZMYND8 puncta in this cell line (Fig. 2B, C), a phenomenon observed in prostate cancer where SPOP-mediated non-degradative ubiquitination decreases p62 puncta formation^23^.

We further performed a rescue experiment by co-transfecting SPOP with ZMYND8-WT or ZMYND8-K398R into Caki-2 ZMYND8-KO cells. We found that SPOP expression only decreased the puncta number of ZMYND8-WT, but not the K398R mutant (Fig. 2D, E). Conversely, in SPOP-high 786-O and A-498 cells, the basal number of ZMYND8 puncta was relatively low; however, SPOP KO significantly increased ZMYND8 puncta formation (Fig. 2F, G), which could be largely diminished by 1,6-hexanediol treatment (Fig. 2H, I). Together, these data suggest that K398 ubiquitination of ZMYND8 mediated by SPOP plays an important role in preventing ZMYND8 from forming phase separation compartments (Fig. 2J).

### SPOP-mediated ZMYND8 ubiquitination promotes ccRCC cell proliferation and p70S6K phosphorylation independently of mTOR

**Fig. 3:**
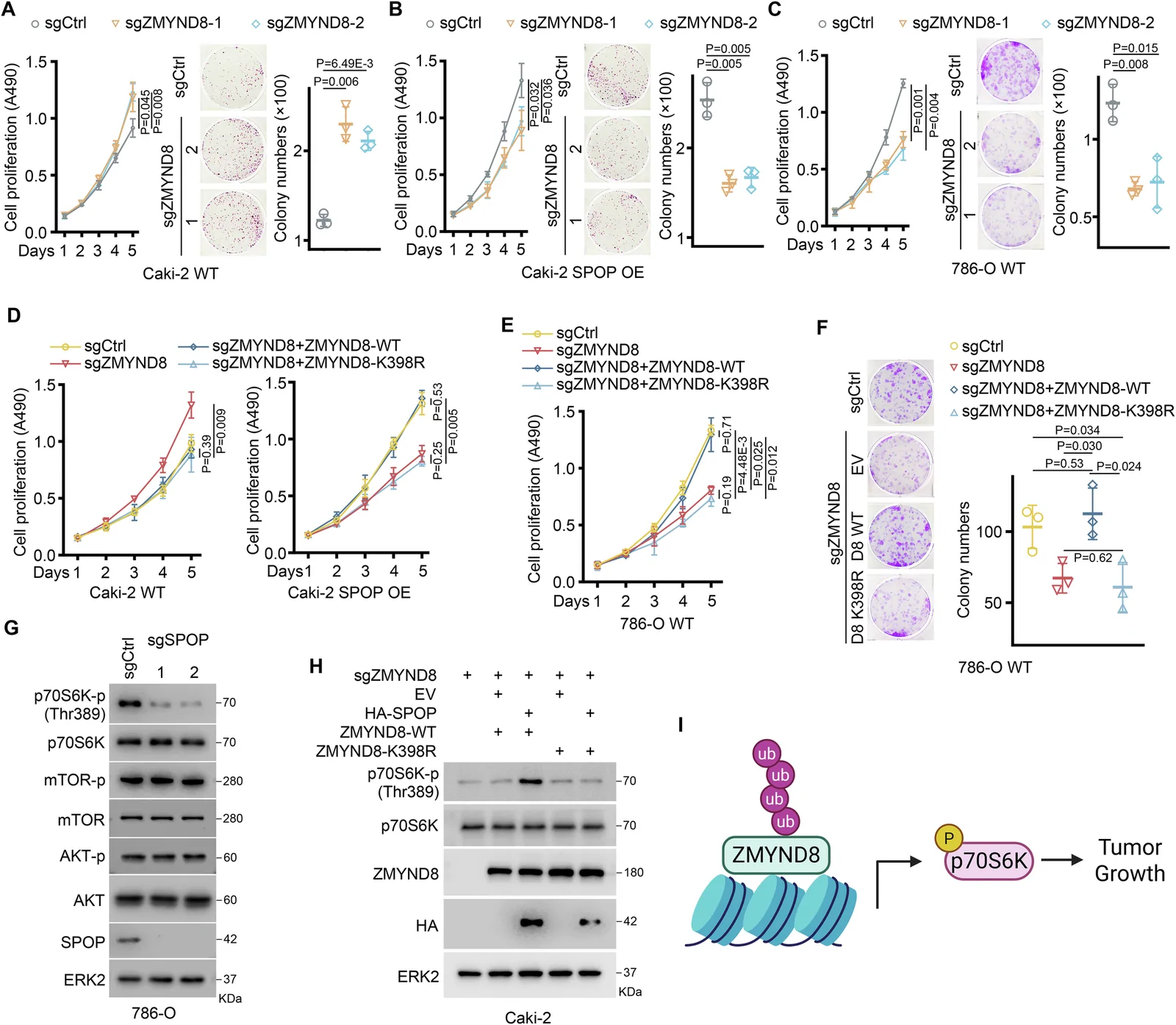
ZMYND8 ubiquitination mediated by SPOP promotes ccRCC cell proliferation and p70S6K phosphorylation independently of mTOR. **A**–**C** Measurement of proliferation by MTS and colony formation assay in Caki-2 WT cells (**A**), Caki-2 SPOP OE cells (**B**) and 786-O WT cells (**C**) infected with lentivirus expressing the indicated sgRNAs. *P* values arranged in order: 0.045, 0.008, 0.006, 6.49E-3, 0.032, 0.036, 0.005, 0.005, 0.001, 0.004, 0.008, 0.015. **D** Measurement of proliferation of mock (left) and SPOP OE (right) Caki-2 cells transfected with indicated plasmids. *P* values arranged in order: 0.39, 0.009, 0.53, 0.25, 0.005. **E**, **F** Measurement of proliferation by MTS (**E**) and colony formation (**F**) assay in 786-O cells infected with lentivirus expressing the indicated plasmids. *P* values arranged in order: 0.71, 0.19, 4.48E-3, 0.025, 0.012, 0.034, 0.030, 0.53, 0.024, 0.62. **G**, **H** WB analysis of indicated proteins in 786-O (**G**) and Caki-2 (**H**) transfected with indicated plasmids. **I** A hypothetical model depicting p70S6K phosphorylation induced by the SPOP-ZMYND8 pathway in a manner independent of the mTOR pathway (Created in BioRender. Qiu, L. (2026) https://BioRender.com/bbtgj1b). WB analyses (**G**, **H**) are representative of three independent experiments with similar results. MTS assays and colony formation assays (**A**–**F**) are obtained in three biological replicates. Data are shown as mean ± SD (**A**–**F**). Statistics: Two-tailed unpaired Student’s *t*-test are used in (**A**–**F**). Source data are provided as a Source Data file.

Since ubiquitination of ZMYND8 at K398 affects phase separation rather than causing protein degradation, we hypothesized that this modification might confer an undefined function. To test this hypothesis, we first examined the effect of ZMYND8 on the growth of SPOP-low Caki-2 cells and found that ZMYND8-KO increased cell proliferation and colony formation (Fig. 3A and Supplementary Fig. 4A), consistent with the previously reported tumor suppressor role of ZMYND8^4,5^. However, ZMYND8 KO had the opposite effects on the growth of SPOP-OE Caki-2 cells (Fig. 3B and Supplementary Fig. 4A). Similarly, ZMYND8 KO in SPOP-high 786-O cells significantly decreased cell proliferation and colony formation (Fig. 3C and Supplementary Fig. 4B), while the opposite effect was observed in an SPOP-KO background (Supplementary Fig. 4C). In WT Caki-2 cells, re-expression of both ZMYND8 WT and K398R mutant decreased cell proliferation (Fig. 3D). However, in SPOP-OE Caki-2 cells, only re-expression of ZMYND8 WT, but not K398R mutant, restored cell proliferation (Fig. 3D). In 786-O cells, MTS and colony formation assays revealed a proliferation phenotype similar to that in SPOP-OE Caki-2 cells (Fig. 3E, F). Restoration of ZMYND8 K398R did not restore cell resistance to everolimus in 786-O cells (Supplementary Fig. 4D). The above results showed a functional switch of ZMYND8 under conditions of SPOP OE and ZMYND8 K398 ubiquitination, leading to mTOR inhibitor resistance through an AKT-mTOR independent signaling pathway.

To elucidate the molecular mechanism underlying ZMYND8 ubiquitination-induced ccRCC cell proliferation, we analyzed the expression of proteins involved in proliferation-related signaling pathways, including the AKT-mTOR and MAPK cascades. We found that ZMYND8-KO decreased the phosphorylation of p70S6K at threonine 389 (p70S6K-p) without affecting upstream effectors in the canonical AKT-mTOR pathway (Supplementary Fig. 4E). Intriguingly, this effect could be reversed by restored expression of ZMYND8 WT, but not the K398R mutant (Supplementary Fig. 4E). To further explore the role of SPOP-ZMYND8 interaction in regulating p70S6K-p level, we knocked out SPOP in 786-O cells and observed a substantial reduction in p70S6K-p level, while phosphorylation of mTOR remained largely unaffected (Fig. 3G). In this cell line, as expected, everolimus inhibited p70S6K-p level through mTOR-p70s6k signaling pathway (Supplementary Fig. 4F). Furthermore, SPOP overexpression increased p70S6K-p level in Caki-2 cells, but only when co-expressed with ZMYND8 WT, not the K398R mutant (Fig. 3H). SPOP-ZMYND8 axis only affected p70S6K Thr389 phosphorylation activity but had little effect on other phosphorylation site of p70S6K, such as Thr229 (Supplementary Fig. 4G, H). These data suggest that SPOP-mediated ubiquitination of ZMYND8 is required for p70S6K Thr389 phosphorylation and ccRCC cell proliferation through a mechanism independently of the AKT-mTOR axis (Fig. 3I).

### Ubiquitination of ZMYND8 promotes p70S6K phosphorylation through NEK7

**Fig. 4:**
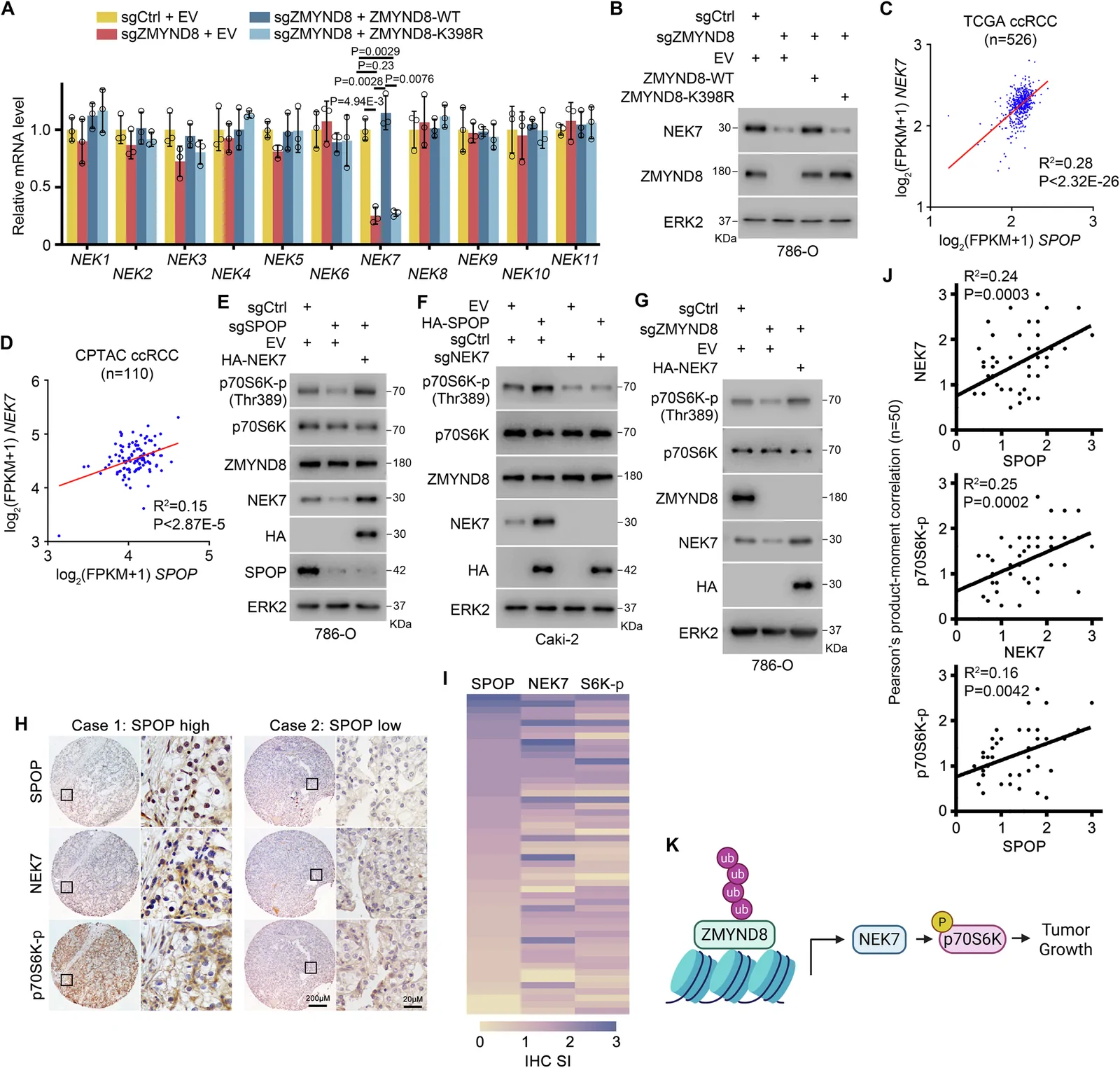
ZMYND8 ubiquitination promotes p70S6K phosphorylation through NEK7. **A** RT-qPCR analysis of mRNA expression of NEK family members in the indicated 786-O cells. Data are shown as mean ± SD derived from three biological replicates. Two-tailed unpaired Student’s *t*-test; *P* values arranged in order: 0.0029, 0.23, 0.0028, 0.0076, 4.94E-3. **B** WB analysis of NEK7 protein expression in indicated 786-O cells. **C** Correlation between SPOP and NEK7 mRNA level in TCGA ccRCC database download from https://www.cbioportal.org/. Pearson correlation analysis *R*^2^ = 0.28; Two-sided test; *P* = 2.32E-26. **D** Correlation between SPOP and NEK7 mRNA level in CPTAC ccRCC database download from https://proteomics.cancer.gov/programs/cptac. Pearson correlation analysis *R*^2^ = 0.28; Two-sided test; *P* = 2.87E-5. **E**–**G** WB analysis of indicated proteins in ccRCC cells transfected with indicated plasmids. **H** Representative images of IHC for SPOP, NEK7, and p70S6K-p-T389 on TMA patient specimens (*n* = 50 TMA elements). **I** Heatmap showing the staining index (SI) of SPOP, NEK7, and p70S6K-p-T389 proteins on TMA. **J** Correlation analysis of the staining index (SI) of SPOP, NEK7, and p70S6K-p-T389 proteins on TMA. Pearson correlation analysis *R*^2^ = 0.24, 0.25, 0.16; Two-sided test; *P* values arranged in order: 0.0003, 0.0002, 0.0042. **K** A hypothetical model depicting the role of the SPOP-ZMYND8-NEK7 axis in phosphorylating p70S6K (Created in BioRender. Qiu, L. (2026) https://BioRender.com/bbtgj1b).WB analyses (**B**, **E**–**G**) are representative of three independent experiments with similar results. Source data are provided as a Source Data file.

Besides mTOR, previous study has reported that members of the NIMA-related kinase (NEK) family could also catalyze p70S6K T389 phosphorylation^24,25^. NEK family comprises eleven members and all of which share similar kinase domains^26^. To determine whether NEK family members are involved in regulation of p70S6K phosphorylation by SPOP-ZMYND8 axis in ccRCC cells, we performed an unbiased survey of mRNA expression of all eleven NEK family members in 786-O cells with or without ZMYND8 expression. We found that ZMYND8-KO specifically decreased mRNA expression of *NEK7*, but not the other ten members. This effect could be reversed by restored expression of ZMYND8 WT, but not the K398R mutant (Fig. 4A). Furthermore, SPOP-KO significantly downregulated *NEK7* and moderately decreased *NEK9* mRNA expression in 786-O cells (Supplementary Fig. 5A). Western blot analysis confirmed that both SPOP and ZMYND8 regulated NEK7 protein expression (Fig. 4B and Supplementary Fig. 5B). Since NEK9 is not regulated by ZMYND8 (Fig. 4A), we focused on determining whether NEK7 is the key mediator of p70S6K phosphorylation regulated by SPOP-ZMYND8 axis.

Analysis of Clinical Proteomic Tumor Analysis Consortium (CPTAC) ccRCC dataset^27^ revealed that both mRNA and protein expression of NEK7 are significantly elevated in primary ccRCC compared with adjacent normal tissues (Supplementary Fig. 5C, D). These findings were corroborated by the tissue microarray (TMA)-based immunohistochemistry (IHC) analysis of NEK7 protein from an independent ccRCC cohort (Supplementary Fig. 5E, F). Next, we sought to determine whether NEK7 phosphorylates p70S6K in ccRCC cells. Co-IP analysis showed that endogenous NEK7 and p70S6K proteins interact with each other in 786-O cells (Supplementary Fig. 5G). NEK7 KO by two independent sgRNAs decreased p70S6K-p Thr389 but not Thr229, without affecting the total protein level of p70S6K (Supplementary Fig. 5H). In contrast, overexpression of NEK7 WT, but not the kinase-dead mutant (NEK7-D179N)^28^ increased p70S6K-p level (Supplementary Fig. 5I). Furthermore, we performed a double knockdown of NEK7 and ZMYND8 in 786-O cells and found that simultaneous knockdown of both proteins did not further reduce p70S6k phosphorylation, showing that NEK7 and ZMYND8 are involved in the same signaling pathway (Supplementary Fig. 5J). These data indicate that NEK7 is overexpressed in a subset of ccRCC and NEK7 overexpression augments p70S6K phosphorylation in ccRCC cells.

Analysis of The Cancer Genome Atlas (TCGA) and CPTAC ccRCC datasets revealed that *SPOP* mRNA level positively correlated with *NEK7* mRNA level in these two cohorts (Fig. 4C, D). We further examined whether SPOP or ZMYND8 regulates p70S6K-p level through NEK7. As expected, SPOP depletion decreased NEK7 expression and p70S6K-p; however, downregulation of p70S6K-p in SPOP KO cells were completely reversed by restored expression of NEK7 (Fig. 4E). Furthermore, NEK7 KO completely abolished SPOP overexpression-induced increase of p70S6K-p (Fig. 4F). Similarly, ectopic expression of NEK7 reversed ZMYND8 KO-induced downregulation of p70S6K-p level (Fig. 4G).

To validate the correlation between SPOP, NEK7 and p70S6K-p expression in human ccRCC specimens, we performed IHC on a TMA derived from an independent ccRCC cohort (*n* = 50). Representative images of low/no and high staining of SPOP, NEK7, and p70S6K-p are shown in Fig. 4H. Consistent with mRNA expression from TCGA and CPTAC database (Fig. 4C, D), SPOP expression positively correlated with NEK7 level (Pearson’s product-moment correlation coefficient *R*^2^ = 0.24, *p* = 0.0003, the same below) (Fig. 4I, J). As a kinase responsible for p70S6K phosphorylation, NEK7 expression positively correlated with p70S6K-p level (*R*^2^ = 0.25, *p* = 0.0002) (Fig. 4I, J). Most importantly, p70S6K-p level positively correlated with SPOP protein level in this cohort (*R*^2^ = 0.16, *p* = 0.004) (Fig. 4I, J). These data imply that NEK7 is a key alternative kinase mediating SPOP-ZMYND8 axis activation-induced p70S6K phosphorylation (Fig. 4K).

### Ubiquitinated ZMYND8 promotes *NEK7* transcription by forming a protein complex with ZHX2

**Fig. 5:**
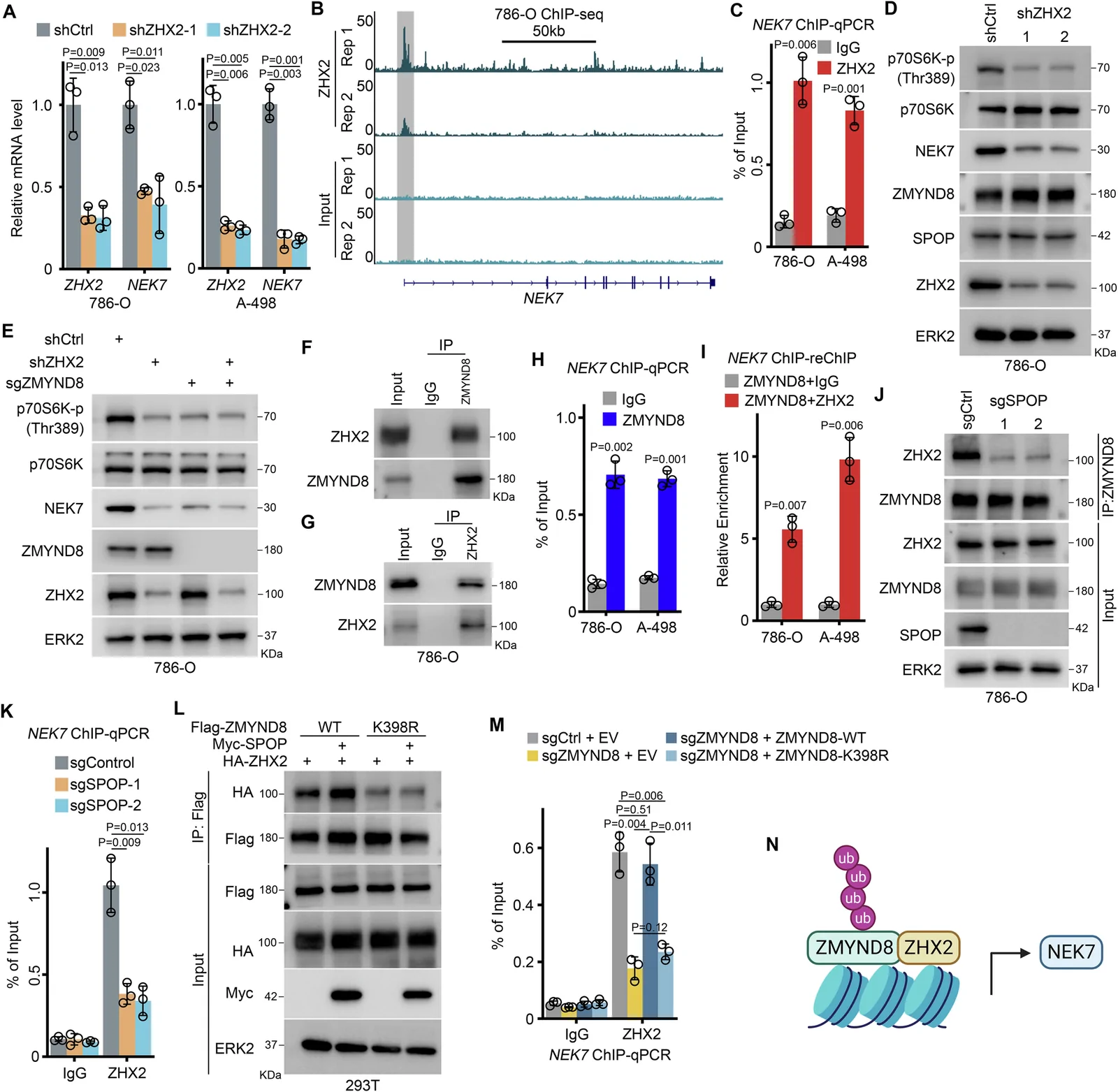
Ubiquitinated ZMYND8 promotes NEK7 transcription by forming a transactivation complex with ZHX2. **A** RT-qPCR analysis of ZHX2 and NEK7 mRNA expression in mock or ZHX2 KD 786-O (left) and A-498 (right) cells. *P* values arranged in order: 0.009, 0.013, 0.011, 0.023, 0.005, 0.006, 0.001, 0.003. **B** UCSC genome browser screen shot of ChIP-seq signal of input control and ZHX2 in the *NEK7* gene locus^34^. **C** ChIP-qPCR analysis of ZHX2 binding at NEK7 promoter in 786-O and A-498 cells. *P* values arranged in order: 0.006, 0.001. **D**, **E** WB analysis of indicated proteins in 786-O cells transfected with indicated plasmids. **F**, **G** Western blot analysis of co-IP of endogenous proteins by anti-ZMYND8 (**F**) and anti-ZHX2 (**G**) antibody in 786-O cells. **H** ChIP-qPCR analysis of ZMYND8 binding at NEK7 promoter in 786-O cells. *P* values arranged in order: 0.002, 0.001. **I** ChIP-reChIP analysis examining the co-localization of ZMYND8 and ZHX2 in the *NEK7* gene promoters in 786-O and A-498 cells. *P* values arranged in order: 0.007, 0.006. **J** WB analysis of co-IP samples of anti-ZMYND8 antibody obtained from WCL of mock or SPOP KO 786-O cells. **K** ChIP-qPCR analysis of ZHX2 binding at *NEK7* promoter in mock or SPOP KO 786-O cells. *P* values arranged in order: 0.013, 0.010. **L** WB analysis of co-IP samples of anti-Flag antibody in WCL of 293 T cells transfected with indicated plasmids. **M** ChIP-qPCR analysis of ZHX2 binding at *NEK7* promoter in 786-O cells transfected with indicated plasmids. *P* values arranged in order: 0.006, 0.51, 0.004, 0.011, 0.12. **N** A hypothetical model depicting the role of the ZMYND8-ZHX2 complex in transactivation of NEK7 gene (Created in BioRender. Qiu, L. (2026) https://BioRender.com/bbtgj1b). WB analyses (**D**–**G**, **J**, **L**) are representative of three independent experiments with similar results. RT-qPCR (**A**), ChIP-qPCR (**C**, **H**, **I**, K, and **M**). Data are shown as mean ± SD derived from three biological replicates. Statistics: Two-tailed unpaired Student’s *t*-test are used in (**A**, **C**, **H**, **I**, **K**, and **M**). Source data are provided as a Source Data file.

To elucidate the mechanism by which SPOP-induced ZMYND8 ubiquitination promotes *NEK7* transcription in ccRCC, we performed an unbiased screening of several reported transcription factors with critical roles in ccRCC, including EPAS1 (also known as HIF2A)^29^, PAX8^30^, RUNX1^31^, TFEB, TFE3^32^, STAT3^33^, and ZHX2^34^. We found that only knockdown of ZHX2, but not other transcription factors examined, decreased *NEK7* mRNA expression in 786-O cells (Fig. 5A and Supplementary Fig. 6A). ZHX2 knockdown also decreased *NEK7* mRNA expression in A-498 cells (Fig. 5A).

A recent study has identified ZHX2 as a VHL target that serves as a transcription activator to drive ccRCC oncogenesis^34^. Meta-analysis of ChIP-seq data indicated that ZHX2 occupies a region proximal to the *NEK7* gene promoter in ccRCC cells (Fig. 5B), whereas HIF2α was not significantly enriched at this region (Supplementary Fig. 6B). ChIP-qPCR analyses confirmed ZHX2 occupancy at *NEK7* gene locus in ccRCC cell lines (Fig. 5C). Furthermore, knockdown of ZHX2 decreased NEK7 protein expression and caused downregulation of p70S6K-p (Fig. 5D).

While knockdown of ZMYND8 or ZHX2 alone decreased p70S6K-p level, their co-knockdown did not result in further reduction in p70S6K-p level (Fig. 5E), suggesting that ZMYND8 and ZHX2 may regulate p70S6K phosphorylation within the same pathway. To test this possibility, we performed co-IP assays and found that endogenous ZMYND8 and ZHX2 interact with each other in 786-O cells (Fig. 5F, G). Meta-analysis of ChIP-seq data^6,34^ suggested that ZMYND8 occupies a region proximal to the *NEK7* gene promoter (Supplementary Fig. 6C), which was further confirmed in 786-O cells by ChIP-qPCR (Fig. 5H). Consistent with the ChIP-seq data that both ZMYND8 and ZHX2 occupy the same region of the *NEK7* promoter (Supplementary Fig. 6C), sequential chromatin immunoprecipitation (ChIP-reChIP) assay confirmed this finding (Fig. 5I). Notably, ZMYND8-ZHX2 interaction was significantly weakened upon SPOP depletion (Fig. 5J), and the occupancy of ZHX2 at *NEK7* gene locus was also reduced due to SPOP KO (Fig. 5K). However, compared to WT ZMYND8, K398R mutant impeded ZHX2-ZMYND8 interaction. In contrast, SPOP overexpression failed to promote the interaction between ZHX2 and ZMYND8 K398R mutant (Fig. 5L), suggesting that SPOP likely enhances ZHX2-ZMYND8 interaction via K398 polyubiquitination. Moreover, ZHX2 occupancy at the *NEK7* gene locus was significantly reduced by knockdown of ZMYND8, which was reversed by re-expression of ZMYND8 WT, but not the K398R mutant (Fig. 5M). Collectively, these data suggest that SPOP-mediated ubiquitination of ZMYND8 at the K398 residue is critical for ZHX2 occupancy at the *NEK7* promoter and for NEK7 expression in ccRCC (Fig. 5N).

### NEK7 inhibition augments the anti-cancer efficacy of mTOR inhibitor

**Fig. 6:**
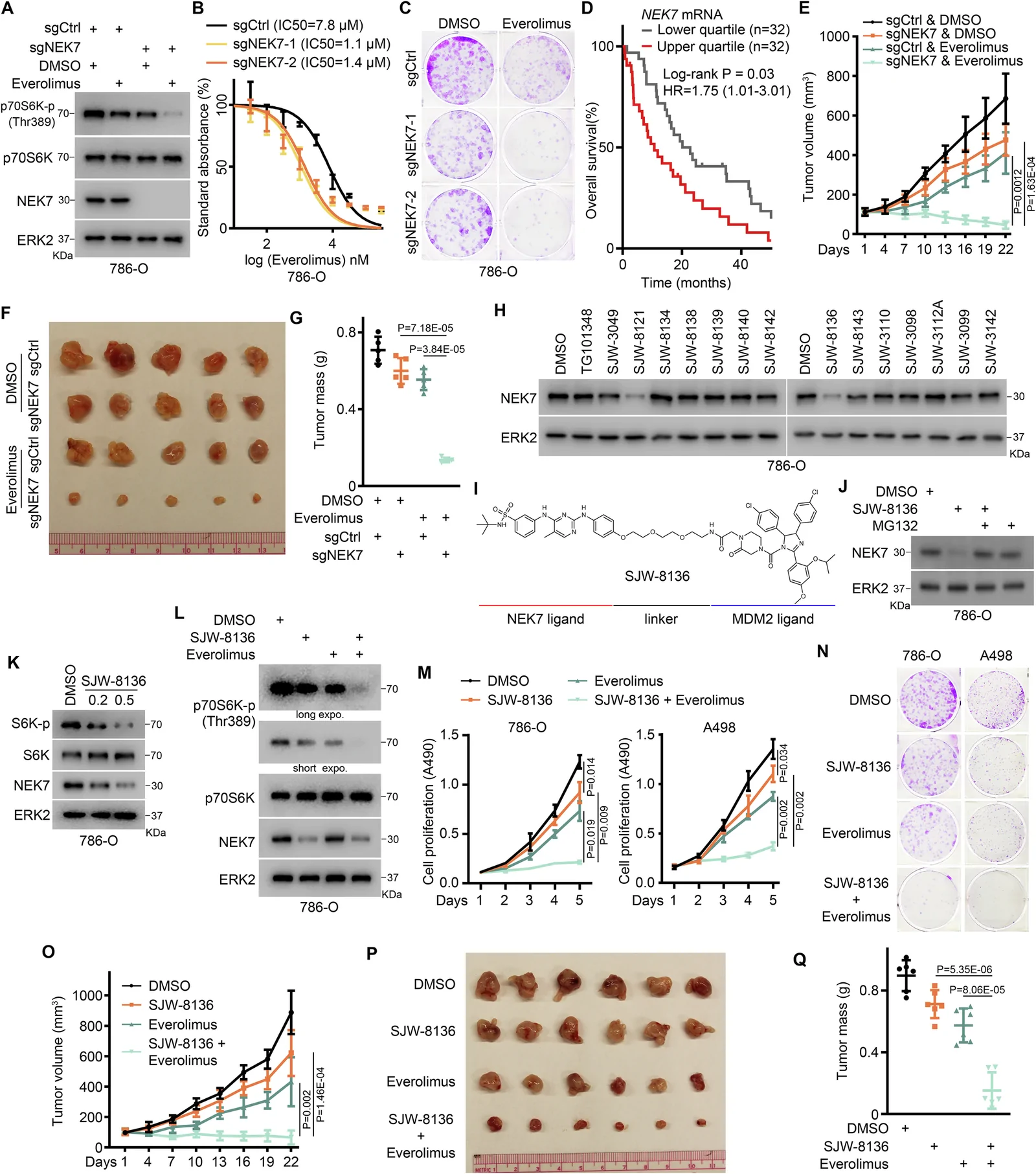
Co-administration of NEK7 degrader and mTOR inhibitor effectively inhibits ccRCC tumor growth. **A** WB analysis of indicated proteins in mock or NEK7 KO 786-O cells treated with DMSO or everolimus (5 Μm, 24 h). **B** IC50 of everolimus in mock or NEK7 KO 786-O cells treated with everolimus for 72 h. **C** Colony formation assay of mock or NEK7 KO 786-O cells treated with DMSO or everolimus (5 μM, 24 h), followed by photograph. **D** Kaplan–Meier curves of ccRCC patients treated with everolimus and stratified by NEK7 mRNA expression level. HR hazard ratio; log-rank test *P* = 0.03. **E**–**G** Growth of mock and NEK7 KO stable 786-O tumors in SCID mice treated with vehicle (DMSO) or everolimus for 22 days (**E**). Tumors were harvested and photographed (**F**) and weight of tumors are shown in (**G**). Data are shown as mean ± SD (*n* = 5 mice per group). *P* values arranged in order: 0.0012, 1.63E-04, 7.18E-05, 3.84E-05. **H** WB analysis of NEK7 protein level in 786-O cells treated with indicated small molecules (1 μM, 24 h). **I** Chemical structures of SJW-8136. **J**–**L** WB analysis of indicated protein in 786-O cells treated with DMSO, SJW-8136 (indicated concentrations, 24 h), everolimus (5 μM, 24 h) and/or MG132 (20 μM, 12 h). **M** MTS assay of 786-O and A498 cells treated with SJW-8136 (0.5 μM) and/or everolimus (5 μM). *P* values arranged in order:0.014, 0.016, 0.009, 0.034, 0.002, 0.002. **N** Colony formation assay of 786-O and A498 cells treated with SJW-8136 (0.5 μM) and/or everolimus (5 μM), followed by photograph. **O**–**Q** Growth of 786-O tumors in SCID mice treated with SJW-8136, everolimus or both for 22 days (**O**). Tumors were harvested and photographed (**P**) and weight of tumors are shown in (**Q**). Data are shown as mean ± SD (*n* = 6 mice per group). *P* values arranged in order: 0.002, 1.46E-04, 8.06E-05, 5.35E-06.WB analyses (**A**, **H**, **J**–**L**) are representative of three independent experiments with similar results. MTS assays (**B**, **M**) and colony formation assays (**C**, **N**) are obtained in three biological replicates. Data are shown as mean ± SD (**B**, **M**). Statistics: Two-tailed unpaired Student’s *t*-test are used in (**E**, G, **M**, **O**, and **Q**). Source data are provided as a Source Data file.

Combined treatment of everolimus and SPOP-IN-6b significantly enhanced inhibition of p70S6K-p level (Supplementary Fig. 7A), suggesting that SPOP-ZMYND8-NEK7 axis and mTOR signaling play complementary roles in promoting p70S6K phosphorylation, which explains why inhibition of SPOP can increase the sensitivity of ccRCC to mTOR inhibitors. However, as a CRL adaptor protein, SPOP has diverse roles in different cancer types. It seems that SPOP acts as a tumor suppressor in prostate cancer but an oncoprotein in ccRCC^14^, which may limit the use of SPOP inhibitor due to its potential carcinogenic activity in other tissues, including the prostate. We have discovered that NEK7 is a key kinase that phosphorylates p70S6K in a mTOR-independent manner. In 786-O cells with NEK7 KO, treatment of everolimus significantly reduced p70S6K-p level (Fig. 6A). Moreover, everolimus sensitivity was increased upon NEK7 depletion in 786-O cells (Fig. 6B, C and Supplementary Fig. 7B). Conversely, exogenous expression of NEK7 in Caki-2 cells counteracted the inhibitory effect of everolimus on p70S6K-p level (Supplementary Fig. 7C) and reduced the sensitivity to this drug (Supplementary Fig. 7D–F).

Analysis of a published database comprising 130 advanced ccRCC patients with everolimus treatment^35^ revealed that high NEK7 expression predicted worse outcomes (Fig. 6D). Consistent with the results obtained from in vitro assays, everolimus treatment displayed stronger inhibitory effect on the growth of NEK7 KO 786-O tumors than the control tumors (Fig. 6E–G). This result is further supported by the expression of Ki67 and cleaved caspase-3 in tumors from the indicated groups (Supplementary Fig. 7G–I). These data suggest that NEK7 is a promising therapeutic target to improve mTOR inhibitor sensitivity in ccRCC.

### Co-administration of NEK7 degrader and mTOR inhibitor effectively inhibits ccRCC tumor growth

At present, there is no specific NEK7 inhibitor available to target NEK-mediated p70S6K activation. Proteolysis-targeting chimera (PROTAC) is a bivalent small molecule that contains one ligand recognizing the target protein and the other for binding of an E3 ubiquitin ligase, which promotes proximity-induced ubiquitination and degradation of the target protein^36^. TG101348, a compound reported to bind with NEK7^37^, served as a structural basis for designing and synthesizing a series of NEK7 PROTACs (13 compounds in total). These PROTACs recruit NEK7 to the proximity of E3 ubiquitin ligases MDM2 (7 compounds) or cereblon (CRBN, 6 compounds) for ubiquitination and degradation (see details in Materials and Methods). Among the NEK7 PROTACs tested, SJW-8121 and SJW-8136 displayed high potency in inducing NEK7 degradation in 786-O cells (Fig. 6H). TG101348 was previously reported to inhibit JAK2 kinase activity and phosphorylation of its target STAT3^38^. Notably, treatment with SJW-8136 (Fig. 6I) did not decrease STAT3 phosphorylation as much as TG101348 and SJW-8121 (Supplementary Fig. 8A), indicating a more specific effect of SJW-8136 on NEK7. Dose-dependent studies showed that SJW-8136 effectively induced NEK7 degradation starting at a concentration of 0.1 μM (Supplementary Fig. 8B), with maximal degradation effect in the range of 0.5–3 μM (Supplementary Fig. 8C). Furthermore, JAK2 knockdown (KD) by pooled shRNAs failed to decrease NEK7 protein level. SJW-8136 treatment decreased NEK7 protein level in JAK2 KD cells to a similar extent as in control 786-O cells, indicating that the effect of SJW-8136 on NEK7 is independent of JAK2 activity (Supplementary Fig. 8D). As expected, SJW-8136 treatment failed to alter NEK7 mRNA expression (Supplementary Fig. 8E) but did induce NEK7 protein degradation and the effect was blocked by the proteasome inhibitor MG132 (Fig. 6J). Moreover, treatment of SJW-8136 induced NEK7 polyubiquitination and significantly shortened the half-life of NEK7 protein in 786-O cells (Supplementary Fig. 8F–H).

Given its selectivity in degrading NEK7 but not JAK2, SJW-8136 was selected for further assessment of its anti-cancer activities. SJW-8136 treatment decreased p70S6K-p level in a dose-dependent manner and the effect correlated with the change of NEK7 protein level (Fig. 6K). Combined treatment of everolimus and SJW-8136 further reduced p70S6K-p to a barely detectable level (Fig. 6L). We also found that, upon NEK7 depletion, SJW-8136 failed to further reduce p70S6K-p level (Supplementary Fig. 8I). Functionally, MTS assay and colony formation assay showed that SJW-8136 only inhibited cell proliferation in NEK7 WT cells but not NEK7 KO cells (Supplementary Fig. 8J–L). These results suggest that SJW-8136 inhibits p70S6K phosphorylation and ccRCC cell proliferation through degradation of NEK7.

We then established the Caki-2 everolimus-resistant sublines (Caki-2 ER, Supplementary Fig. 8M). WB analysis showed that NEK7 protein expression was slightly upregulated in drug-resistant cell lines (Supplementary Fig. 8N). Importantly, treatment with NEK7 PROTAC SJW-8136 restored the sensitivity of Caki-2 ER cells to everolimus (Supplementary Fig. 8O). Combination of everolimus and SJW-8136 significantly inhibited cell proliferation compared to each treatment alone (Fig. 6M, N and Supplementary Fig. 8P). Consistent with the results of in vitro assays, co-administration of everolimus and SJW-8136 was more potent in decreasing p70S6K phosphorylation and suppressing the growth of 786-O cell-derived tumors than everolimus alone in mice (Fig. 6O–Q and Supplementary Fig. 8Q) while having little or no significant effect on mouse body weight (Supplementary Fig. 8R). Taken together, these results suggest that SJW-8136 could effectively decrease NEK7-mediated p70S6K phosphorylation and enhance the mTOR inhibitor sensitivity in ccRCC tumors in vivo.

## Discussion

**Fig. 7:**
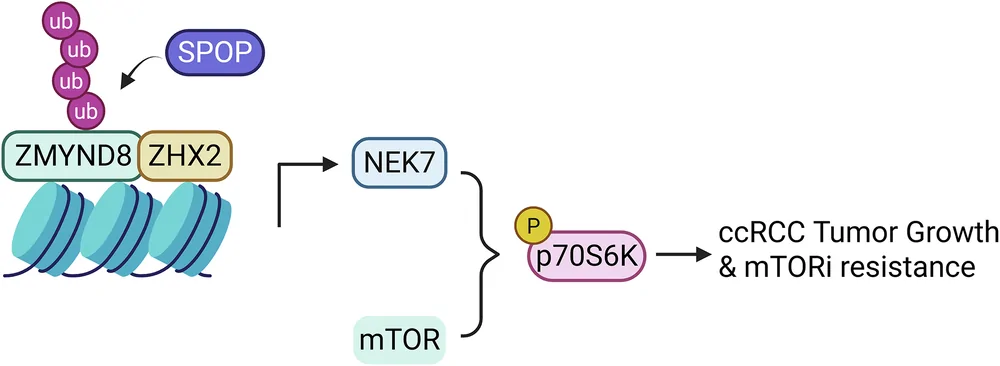
Schematic model. SPOP acts as a bona fide E3 ubiquitin ligase that mediates K63-linked polyubiquitination of ZMYND8, which enables an assembly of the ZMYND8-ZHX2 transactivation complex that promotes transcriptional upregulation of oncogenic genes such as NEK7. The upregulated NEK7 phosphorylates p70S6K, which drives proliferation of cancer cells and leads to the resistance of mTOR inhibitors such as everolimus (Created in BioRender. Qiu, L. (2026) https://BioRender.com/bbtgj1b).

The role of SPOP in cancer is complex as previous studies suggest that SPOP can function as either an oncogene or tumor suppressor depending on the cellular contexts^14^. SPOP is often overexpressed and acts as an oncoprotein in ccRCC through different mechanisms, including targeting several important tumor suppressors (e.g., PTEN, SETD2, and LATS1) for degradation in the cytoplasm^11–13^. In the present study, we identify SPOP as a bona fide E3 ubiquitin ligase that mediates K63-linked polyubiquitination of ZMYND8, which enables an assembly of the ZMYND8-ZHX2 transactivation complex that promotes expression of *NEK7*, cancer cell growth and drug resistance (Fig. 7).

ZMYND8 functions as a chromatin reader that recognizes histone codes, such as methylation (e.g., H3K4me1, H3K4me3, and H3K26me2) and/or acetylation (e.g., H3K14ac and H3K16ac). Given its multiple domains (PHD, BROMO, and PWWP) recognizing various posttranslational modifications, it is not surprising that the role of ZMYND8 in cancer is far more complicated than previously understood. While reported as a tumor suppressor by regulating the enhancer activity and inhibiting multiple metastasis–associated genes^5^, it has also been implicated as an oncogene by activating HIF and YAP^6,39^. Recent studies suggest that ZMYND8 forms liquid compartments with NF-κB/p65 to silence latent super-enhancers^20^. Our previous work demonstrated that ZMYND8 is overexpressed and functions as an oncogene in ccRCC^7^. In this study, we further identified a role of ZMYND8 ubiquitination at lysine 398 located in its IDR region^20^, which prevents ZMYND8 from forming liquid compartments and facilitates formation of a cancer-promoting complex with ZHX2, establishing K398 ubiquitination as a functional switch of ZMYND8 (Fig. 7). Although the detail mechanism remains to be investigated, previous studies imply that ubiquitin binding disrupts LLPS by interfering with multivalent interactions among specific protein region, which is required for LLPS formation^22^. Currently, there are no small-molecule drugs targeting ZMYND8, but our study reveals that SPOP can bind to the C-terminus of ZMYND8 and mediate its ubiquitination by interacting with ZMYND8 in the MYND region. The MYND domain is a zinc-binding protein–protein interaction module presented in transcriptional regulators such as ZMYND family proteins. Its small, highly conserved β-β-α fold region mediates recognition of proline-rich motifs, making it critical for chromatin recruitment and transcriptional control. However, drugging the MYND domain is challenging because its interaction surfaces are relatively flat and rely on multiple weak contacts rather than deep pockets. This limits opportunities for conventional small-molecule inhibitors. Emerging approaches include designing peptide mimetics, fragment-based binders, or molecular glues to modulate MYND-mediated complexes. Thus, while difficult, the MYND domain remains a tractable but demanding drug target.

Kidney cancer is one of the most common cancer types worldwide and ccRCC is the major subtypes, accounting for approximately 75% of all cases^40^. While early-stage patients often have good prognosis following surgical resection and multidisciplinary treatments, about 20–25% of patients are diagnosed at advanced stage with a low rate of 5-year survival and poor life quality^41^. Antiangiogenic therapy and immunotherapy are first-line therapies for advanced ccRCC^42^. Although AKT-mTOR pathway has been reported to be linked to ccRCC tumorigenesis and progression^15^, mTOR inhibitors are currently the adjuvant therapy choice^16^. Here, we identify NEK7 as an alternative kinase promoting p70S6K phosphorylation independently of AKT-mTOR signaling, which is thought to be widely activated in ccRCC^43,44^. Our findings also suggest that aberrant upregulation and activation of NEK7 induced by SPOP overexpression and ZMYND8 polyubiquitination contribute to mTORi resistance in ccRCC (Fig. 7).

As SPOP has diverse roles in different cancer types, inhibiting SPOP in kidney cancer might have adverse effects in other organs, such as the prostate, limiting its clinical potential. To target the cancer-promoting SPOP-ZMYND8-ZHX2-NEK7 axis, we designed the NEK7-targeting PROTAC SJW-8136 by linking a NEK7-binding moiety derived from TG101348 to a MDM2 binding ligand. As TG101348 is a JAK2 inhibitor^38^, it is not surprising that SJW-8136 retains certain activity of JAK2 inhibitor. Most importantly, SJW-8136 exhibited little or no activity in degrading JAK2, suggesting a specific action on NEK7. Given that STAT3 is often aberrantly activated in human cancers and its inhibition suppresses tumor growth in vitro and in vivo^45^, SJW-8136 could be a potential anti-cancer agent due to its bilateral activities in ccRCC.

Compared with conventional small-molecule inhibitors, PROTACs offer several distinct advantages. First, rather than relying on continuous target occupancy to suppress enzymatic activity, PROTACs act catalytically by recruiting E3 ubiquitin ligases to induce proteasomal degradation of the target protein. This event-driven mechanism can lead to more profound and sustained suppression of signaling, even at lower drug concentrations. Second, PROTACs can eliminate both enzymatic and non-enzymatic functions of the target protein, thereby overcoming the limitations of inhibitors that block only the active site. Third, PROTACs have the potential to circumvent resistance mutations that reduce inhibitor binding affinity, since degradation depends on ternary complex formation rather than high-affinity inhibition of the catalytic pocket. Finally, because PROTACs are not consumed in the degradation process, a single molecule can induce multiple rounds of target elimination, which may translate into improved potency and reduced dosing requirements. Taken together, these properties highlight PROTACs as a promising therapeutic strategy with advantages that extend beyond traditional inhibition.

In summary, we identify SPOP as a bona fide E3 ubiquitin ligase for ZMYND8 and propose that SPOP-mediated polyubiquitination of ZMYND8 tips the function of ZMYND8 from tumor suppression to cancer promotion. We reveal that ubiquitinated ZMYND8 forms a transactivation complex with ZHX2 and promotes NEK7 expression. As a result, NEK7-mediated p70S6K phosphorylation confers ccRCC resistance to mTOR inhibitor, which can be overcome by a NEK7-targeting PROTAC. These findings not only nominate NEK7 as a potential therapeutic target in ccRCC, but also imply that NEK7 PROTAC could be a viable arsenal to fight against the mTOR inhibitor tolerance in ccRCC patients.

## Methods

All research was conducted in compliance with all relevant ethical regulations. The study protocol was reviewed and approved by the Mayo clinic Ethics Committee. Further information and requests for resources and reagents should be directed to and will be fulfilled by the corresponding authors.

### Cell lines and cell culture

786-O (Cat#CRL-1932), A498 (Cat#HTB-44), Caki-2 (Cat#HTB-47) and 293 T (Cat#CRL-3216) cell lines were purchased from American Type Culture Collection (ATCC). A498 and 293 T cells were maintained in DMEM supplemented with 10% FBS. 786-O cells were cultured in RPMI 1640 supplemented with 10% FBS. Caki-2 cells were cultured in McCoy’s 5A supplemented with 10% FBS. All cell lines were authenticated by STR profiling and cultured in incubator with 37 °C and 5% CO_2_.

### Stable cell line generation

Lentivirus transduction system was utilized to generate stable cell lines with specific gene knockdown or overexpression. PEI (Sigma-Aldrich, Cat#408727) was used to transfect sgRNA/shRNA plasmids together with lentivirus package plasmids (PSPAX2 and PMD2.G) into 293 T cells. 48 h after transfection, supernatant containing viruses was collected, filtered and utilized to infect indicated cells. Polybrene (Sigma-Aldrich, Cat#TR-1003-G, 8 μg/ml) was added to the viral supernatant to increase the infection efficiency. 48 h after infection, culture medium was replaced with fresh medium, and puromycin (Selleckchem, Cat#S7417, 1 μg/ml) was administrated for cell selection. sgRNA and shRNA sequence information is provided in Supplementary Table 1.

### Clinical samples, tissue microarray (TMA) and immunohistochemistry (IHC)

A tissue microarray was constructed from the formalin-fixed, paraffin-embedded (FFPE) samples of ccRCC, identified after a search of pathologic and clinical databases of archival tissues collected originally with written informed consent from patients^46^. The Mayo Clinic institutional review board (IRB) approved the experimental protocols for retrieving pathology blocks/slides and for accessing electronic medical records. The TMA contained 50 paired cores (one from tumor region and one from adjacent normal tissue region) of 50 patients. Four-micron thick sections were cut from FFPE blocks of human ccRCC specimens. IHC was performed as described previously. IHC analysis of tumor samples was performed using primary anti-SPOP (Proteintech, Cat#16750-1-AP, 1:500), anti-NEK7 (Bethyl Lab Inc., Cat#A302-684A, 1:500), anti-p70S6K-p (Cell Signaling Technology, Cat#9205, 1:200) antibodies, anti-Ki67 (Abcam, Cat# ab15580, 1:10000), anti-Cleaved Caspase-3 (Cell Signaling Technology, Cat#9579, 1:1000). Staining index (SI) was calculated as follows: Staining intensity (intensity: 0 = no staining, 1 = low staining, 2 = medium staining, and 3 = strong staining) and staining percentage was graded accordingly. A final SI score for each staining was obtained by multiplying values of staining percentage and intensity.

### Co-immunoprecipitation (co-IP) and Western blot (WB)

For co-IP assay, cells were lysed with IP buffer (150 mM NaCl, 50 mM Tris-HCl pH = 7.5, 1% Nonidet P-40, 0.5% sodium deoxycholate and 1% protease inhibitor cocktails) for 30 min, and cell lysate was harvested by centrifuging followed by incubation with indicated antibodies and Protein G Plus/Protein A agarose beads (Sigma-Aldrich, USA) at 4 °C overnight. Next day, the beads bound by target proteins were washed 6 times with IP buffer. Proteins were denatured for WB analysis. For WB, target proteins were denatured using sample buffer supplied with 10% DTT (Thermo Fisher Scientific, USA) and denatured at 95 °C for 10 min. Samples were subjected to SDS-polyacrylamide gel (Bio-Rad, USA) separation, and the gels were further transferred to nitrocellulose (NC) membranes (Thermo Fisher Scientific, USA). After transferring, the NC membranes were blocked in 5% non-fat milk (Bio-Rad, USA) for 1 h at room temperature and incubated with the indicated primary antibodies at 4 °C overnight. Next day, the NC membranes were washed with TBST for 10 min each for three times and incubated with matched secondary antibody for 1 h at room temperature. The membranes were washed with TBST for 10 min each for three more times. The protein was visualized by SuperSignal West Pico Stable Peroxide Solution (Thermo Fisher Scientific). Antibody information can be found in Supplementary Table 2.

### Mass spectrometry analysis

Liquid chromatography‒mass spectrometry (LC‒MS) analysis was performed according to a previously published procedure^47^. To identify ZMYND8 ubiquitination sites, 293 T cells expressing HA-tagged/Flag-tagged ZMYND8, Ub and SPOP were harvest and then lysed. The lysates were purified using anti-HA or anti-Flag-agarose beads. The bound proteins were eluted with 2% SDS and digested overnight with sequencing-grade modified trypsin (Promega, #V5111). The obtained peptides were analyzed using a nanoflow EASY-nLC 1200 system (Thermo Fisher Scientific, Odense, Denmark) coupled to an Orbitrap Exploris480 mass spectrometer (Thermo Fisher Scientific, Bremen, Germany). The data were searched against the UniProt human protein database (75,004 entries, download on 07-01-2020) using Protein Discoverer (version 2.4.1.15, Thermo Fisher Scientific) and Mascot (version 2.7.0, Matrix Science).

### Quantitative real-time polymerase chain reaction (RT-qPCR)

Total RNA was extracted from the cells by utilizing Trizol reagent (Ambion, USA) and reversely transcribed into cDNA by utilizing the GoScript kit (Promega, USA). The SYBR-Green Mix (Bio-Rad, USA) and CFX96 Real-Time System (Bio-Rad, USA) were utilized to conduct the real-time PCR according to the manufacturer’s instructions. Expression of the GAPDH gene was used as inner control. Sequence information for primers used for RT-qPCR is provided in Supplementary Table 3.

### Chromatin immunoprecipitation and quantitative PCR (ChIP-qPCR)

ChIP-qPCR was performed as previously described^48^. Briefly, formaldehyde (10%) solution was utilized to crosslink chromatin in cells for 10 min at room temperature. Crosslinked chromatin was sonicated and immunoprecipitated with Protein G Plus/Protein A agarose beads (Sigma-Aldrich, USA) together with the indicated antibody at 4 °C overnight. The Protein-DNA complexes were precipitated and eluted and cross-linking was reversed at 65 °C for 16 h. DNA fragments were purified and analyzed by real-time PCR. Sequence information for primers used for ChIP-qPCR is provided in Supplementary Table 3.

### MTS cell proliferation assay

Cell proliferation was measured utilizing the MTS assay (Promega, USA) according to the manufacturer’s instructions. Briefly, cells were seeded in a 96-well plate with a density of 1,000 cells per well. At the indicated time points, 20 ml CellTiter 96 R AQueous One Solution reagent (Promega, USA) was added to cells. After incubating at 37 °C incubators for 2 h, cell growth was measured in a microplate reader with absorbance at 490 nm. The values of each time point are normalized with the value of 0 h and shown in fold change.

### Colony formation assay

Cells were re-suspended in a culture medium and seeded in 6-well plates with a density of 1,000 cells/well. Cells were incubated for 2 weeks. The culture medium was aspirated and the colonies were washed twice with PBS and fixed with methanol and acetic acid (1:3) for 2 h. The colonies were further stained by 0.5% crystal violet for 4 h and washed with water twice to remove the crystal violet. The number of colonies (the colonies with cell numbers over 50) in each well was counted.

### Immunofluorescent chemistry (IFC) and quantification

786-O, A-498, Caki-2, or 293 T cells were seeded on 13-mm glass coverslips. After washing once in ice-cold phosphate-buffered saline (PBS), the coverslips were fixed with 4% formaldehyde in PBS for 15 min at room temperature. Before incubation with primary antibodies, the coverslips were permeabilized in 0.5% Triton in PBS for 15 min. The samples were incubated with primary antibodies for 2 h, washed at least three times with 0.01% Tween 20 in PBS (PBS-T), and subsequently incubated with secondary fluorescence-coupled antibodies for 1 h. After three times’ washing, incubations with antibodies were performed in 3% bovine serum albumin in PBS-T. The coverslips were stained with 4′,6-diamidino-2-phenylindole at 10 mg/ml for 5 min before being mounted on glass slides and visualized using a confocal super-resolution microscope. For analyzing puncta numbers, cell images were threshold and processed by the Analyzed Particles function in ImageJ. Puncta with a size larger than 0.1 μm^2^ were counted. For each biological repeat, 20 cells were re-analyzed in each group and the average number of puncta/cells was quantified.

### Mouse xenograft and tumor analysis

NOD-SCID male mice in the age of 6-week-old were utilized in this study. All the mice were housed in standard condition with a 12-h light/12-h dark cycle and access to food and water ad libitum. Mice were randomly divided into indicated groups. A total number of 5 × 10^6^ 786-O cells (WT or NEK7 KO) were injected subcutaneously into the left and right flanks of mice, together with 50 ml Matrigel matrix (BD Bioscience, USA). Growth of xenografts was monitored every three days by calipers externally. Once the tumor size reached 100 mm^3^ (approximately 3 weeks after injection), mice carrying similar type of tumors were randomized into different groups. The treatment groups were administered as follows: DMSO (DMSO dissolved in PBS, ip, every 2 days, *n* = 6); everolimus (5 mg/kg, po, every 2 days, *n* = 6); SJW-8136 (20 mg/kg, ip, every 2 days, *n* = 6); or a combination of everolimus and SJW-8136 (*n* = 6). The allowed maximal tumor size is 2 cm in any direction based on the institutional tumor production policies and none of the tumors exceeded this size at any point. In due day, mice were sacrificed and tumors were excised for analysis of their sizes and their weights. The protocols for these mice experiments were approved by Mayo Clinic IACUC General Information A00005704-20.

### Protein quantification

Protein expression levels were quantified from western blot bands using ImageJ software (https://imagej.nih.gov/ij/). Relative levels for target protein expression were determined by normalizing with the western blot band intensity of control proteins.

### Statistics analysis

All data are expressed as means ± SD. For experiments with only two groups, Student’s *t*-test was used for statistical comparisons. For analysis of correlation between SPOP, NEK7 and p70S6K-p protein expression in human ccRCC specimens, Pearson’s product-moment correlation was used. *P* < 0.05 was considered statistically significant.

### Reporting summary

Further information on research design is available in the Nature Portfolio Reporting Summary linked to this article.

## Supplementary information

Supplementary Information

Reporting Summary

Transparent Peer Review file

## Source data

Source Data

## Data Availability

The mass spectrometry proteomics data have been deposited to the ProteomeXchange Consortium via the PRIDE partner repository^49^ with the dataset identifier PXD058853. The public database GEO code cited in the article is as follows: GSE109953, GSE98015 and GSE108833. Source data are provided with this paper. The remaining data are available within the Article, Supplementary Information or Source Data file. Source data are provided with this paper.
