# Supplementary material for: SPOP-mediated ZMYND8 ubiquitination and phase separation exclusion drives mTOR inhibitor resistance in kidney cancer
Source: Nat Commun. 2026 Aug 25;17:10147. doi: 10.1038/s41467-026-77043-9 (PMC13601609; doi:10.1038/s41467-026-77043-9)
Supplement: Supplementary file 1 [file 41467_2026_77043_MOESM1_ESM.pdf]

Supplementary Information

SPOP-mediated ZMYND8 ubiquitination and phase separation exclusion drives mTOR inhibitor resistance in kidney cancer

Bo Tang, Rui Sun, Jingwei Shao, Qi-You Wu, Yuqian Yan, Dejie Wang, Zhixian Yu, Lianxin Hu, Yicheng Chen, Chengheng Liao, Qiang Wei, Yige Bao and Haojie Huang

Supplementary Information includes:

Supplementary Figures S1 to S8
Supplementary Tables S1 to S4
Supplementary Materials and Methods

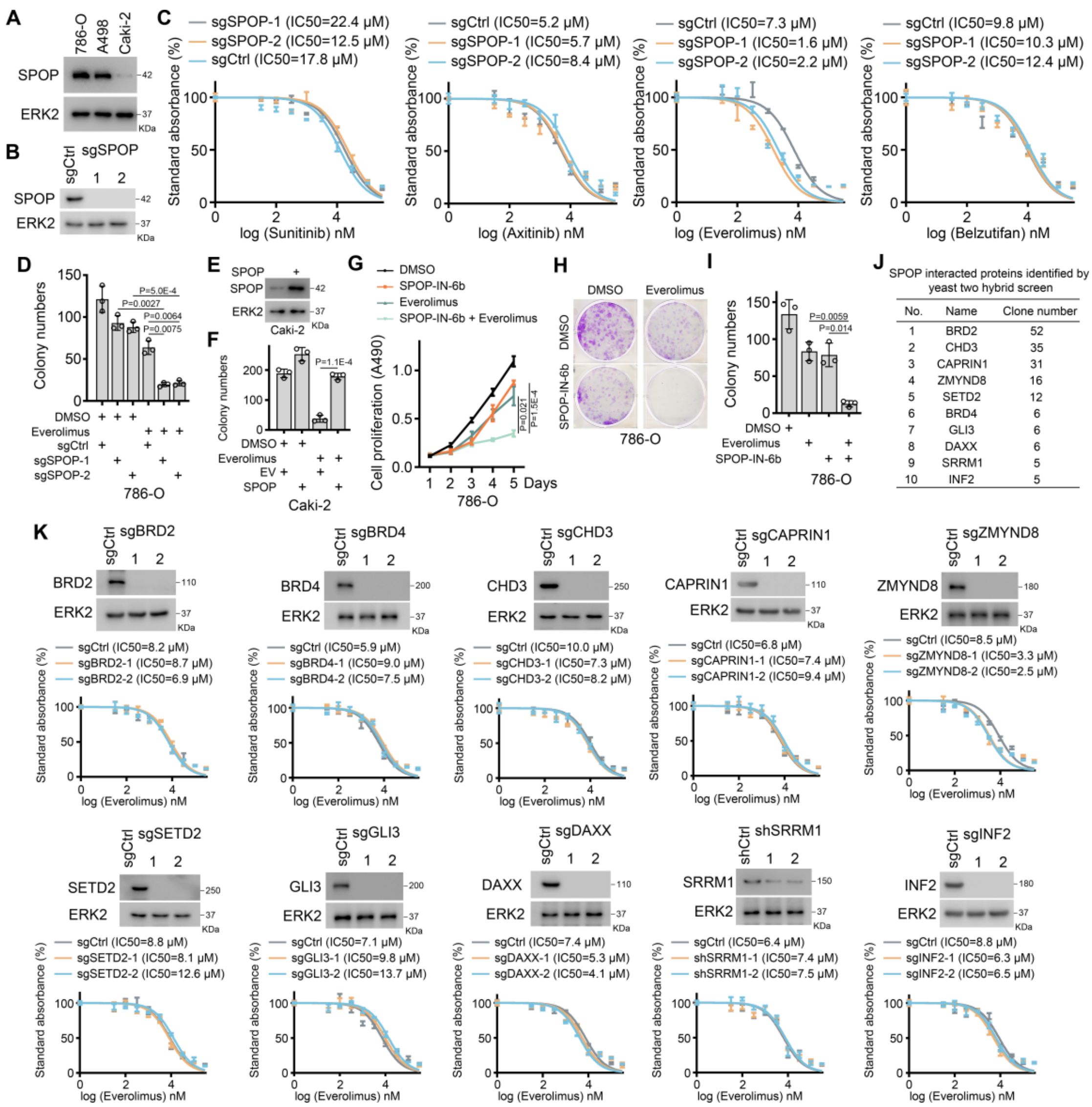

Fig. S1. SPOP Promotes Resistance to mTOR Inhibitors in ccRCC via ZMYND8. (Supp to Fig. 1)

(A and B) WB analysis of SPOP protein level in indicated ccRCC cell lines (A) infected with lentivirus for sgControl or sgSPOP (B).

(C) IC50 of indicated drugs in mock or SPOP KO 786-O cells treated with different concentrations for 72 h.

(D) Quantification of colony formation from Fig. 1 B. *P* values arranged in order: 5.0E-4, 0.0027, 0.0064, 0.0075.

(E) WB analysis of SPOP protein level in Caki-2 cells infected with lentivirus for mock or SPOP plasmids.

(F)Quantification of colony formation from Fig. 1 D. *P*=1.1E-4.

(G)MTS assay of 786-O cells treated with SPOP-IN-6b (2 μM) and/or everolimus (5 μM). Two-tailed unpaired Student's t-test; *P* values arranged in order: 1.5E-4, 0.021.

(H and I) Colony formation assay of 786-O cells treated with SPOP-IN-6b (2 μM) and/or everolimus (5 μM) followed by photograph (H) and quantification (I). *P* values arranged in order: 0.0059, 0.014

(J) Table summarizes top ten vital proteins identified by two-hybrid screens.

(K) WB analysis and 72 h IC50 of everolimus in 786-O cells transfected with indicated plasmids.

WB analyses (A, B, E, K) are representative of three independent experiments with similar results. MTS assays (C, G, K), colony formation assays (D, F, H, I) are obtained in three biological replicates. Data are shown as mean ± SD (D, F, G, I and K). Statistics: Two-tailed unpaired Student's t-test are used in (D, F, G, I). Source data are provided as a Source Data file.

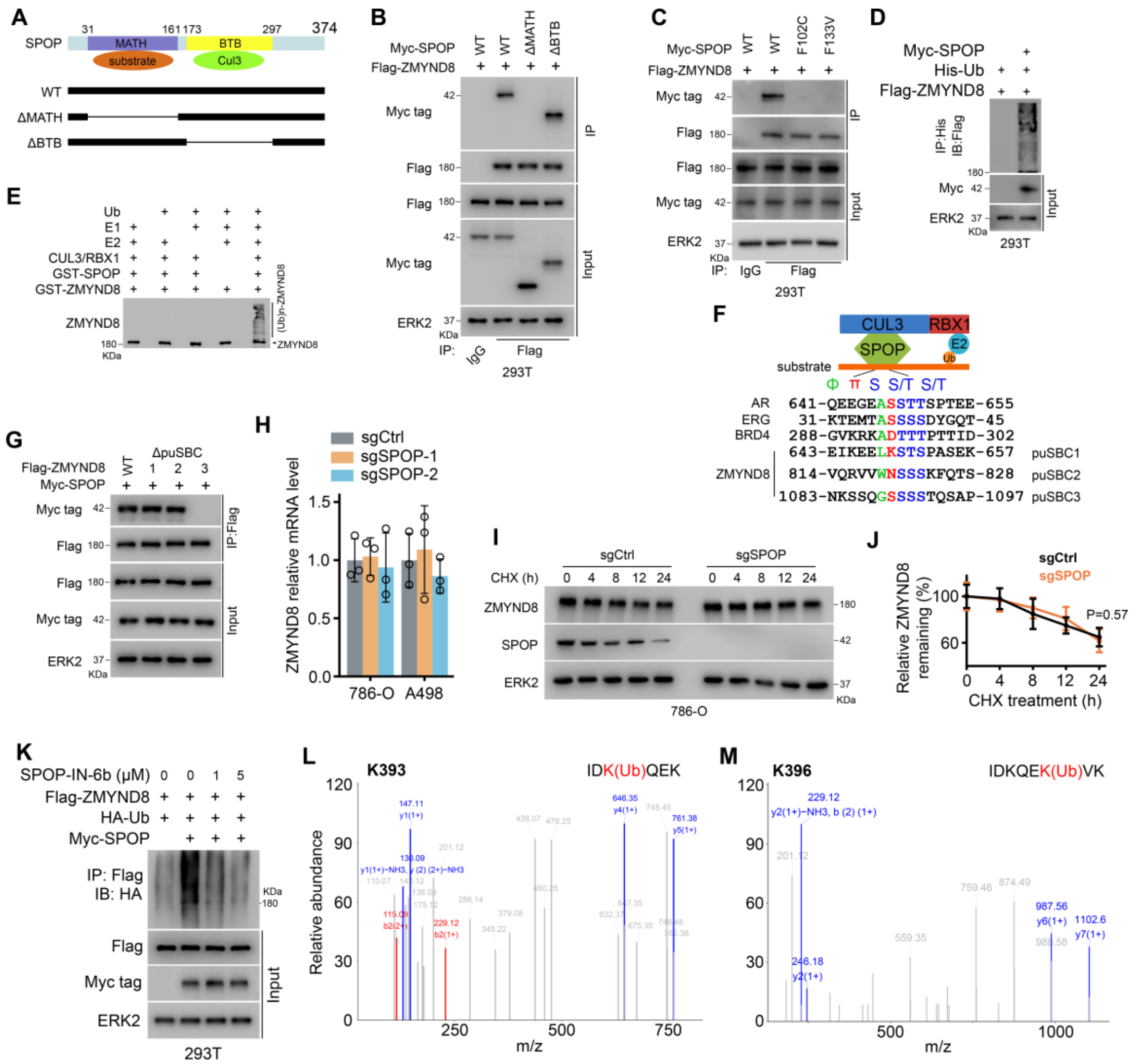

Fig. S2 SPOP Mediates K63-linkage Polyubiquitination of ZMYND8 at Lysine-398. (Supp to Fig. 1)

(A)Schematic diagram showing functional domains in SPOP and deletion mutants of SPOP.

(B-D)WB of co-IP samples of IgG or Flag antibody in whole cell lysate (WCL) of 293T transfected with indicated plasmids.

(E) WB analysis of the products of in vitro ubiquitination assay performed by incubating the reconstituted E3 Ub ligase complex (CUL3-RBX1, GST-SPOP) with E1, E2, Ub, and GST-ZMYND8 at 30℃ for 2 hr.

(F) Schematic showing the SPOP-CUL3-RBX1 E3 Ub ligase complex and SPOP SBC motifs in AR, ERG, BRD4, and three putative SBC motifs in ZMYND8.

(G)WB of co-IP samples of Flag antibody in WCL of 293T transfected with indicated plasmids.

(H) RT-qPCR analysis of ZMYND8 mRNA expression in 786-O and A498 cells stably expressing control (sgControl) or SPOP-specific sgRNAs (sgSPOP-1 or sgSPOP-2). Data are shown as mean±SD derived from three biological replicates.

(I) WB analysis of indicated proteins in mock or SPOP KO 786-O cells treated with DMSO or cycloheximide (CHX) for the indicated periods of time.

(J) WB in (I) were quantified and normalized with the value of the 0-hour time point and are shown as line graphs. Data are shown as mean ± SD (three biological replicates, n=3). Two-tailed unpaired Student's t-test; P =0.57.

(K) WB analysis of co-IP samples in 293T cells transfected with indicated plasmids and treated with SPOP-IN-6b for 12 h at the indicated concentrations.

(L and M) Identification and quantification of ZMYND8 K393 and K396 ubiquitination.

WB analyses (B-E, G, I, K) are representative of three independent experiments with similar results. Source data are provided as a Source Data file.

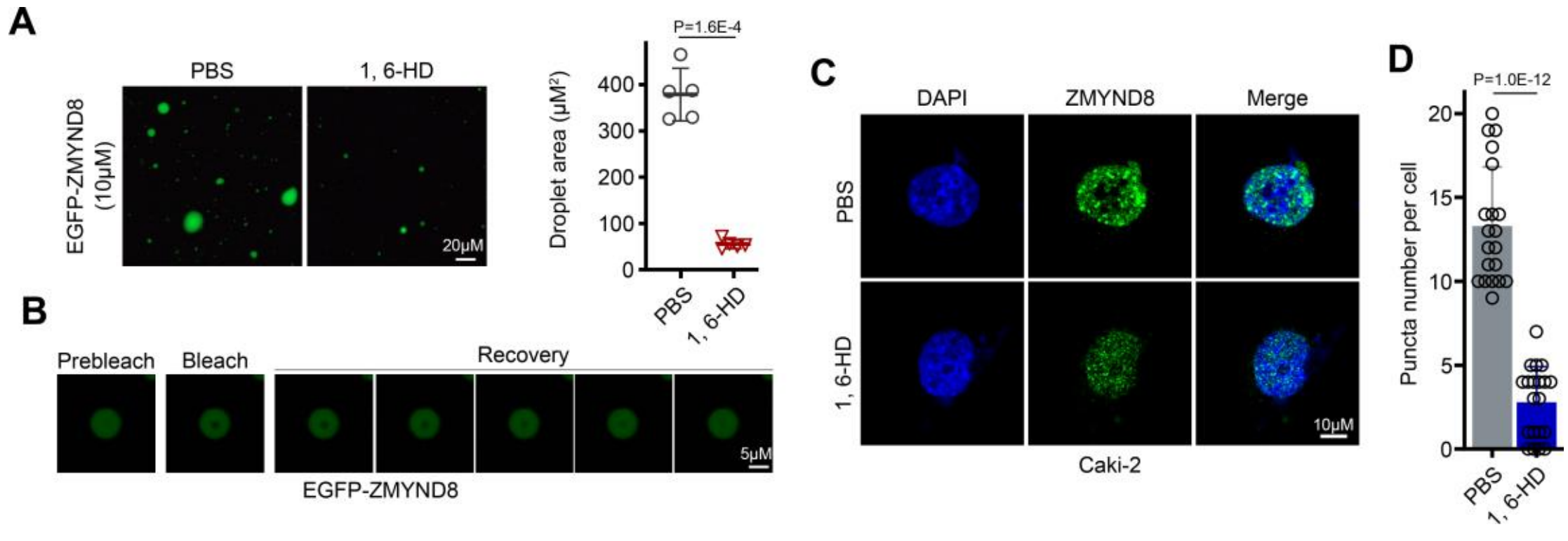

Fig. S3. SPOP Inhibits Liquid Compartment Formation of ZMYND8. (Supp to Fig. 2)

(A) LLPS droplets of EGFP-ZMYND8 (10 μM) are sensitive to 1,6-hexanediol (10%). Dot plots show decreases of droplet area per field in the presence of 1,6-hexanediol. Data are shown as mean ± SD from 5 randomly selected fields from three biological replicates. Two-tailed unpaired Student's t-test; *P* = 1.6E-4

(B) Photo-bleaching analysis of EGFP-ZMYND8 (30 μM) droplets as assessed by fluorescence recovery in a buffer containing 150 mM NaCl and 10% PEG-8000. Confocal images were taken at indicated time points relative to photo bleaching (0 s).

(C) Immunofluorescent cytochemistry (IFC) of Caki-2 cells treated with DMSO or 1,6-hexanediol.

(D) Quantification of ZMYND8 puncta number in cells from (A). Data are shown as mean ± SD from 20 randomly selected fields from three biological replicates. Two-tailed unpaired Student's t-test; *P* = 1.0E-12.

LLPS droplets(A), Photo-bleaching(B), and IFC (C) are representative of three biological replicates. Source data are provided as a Source Data file.

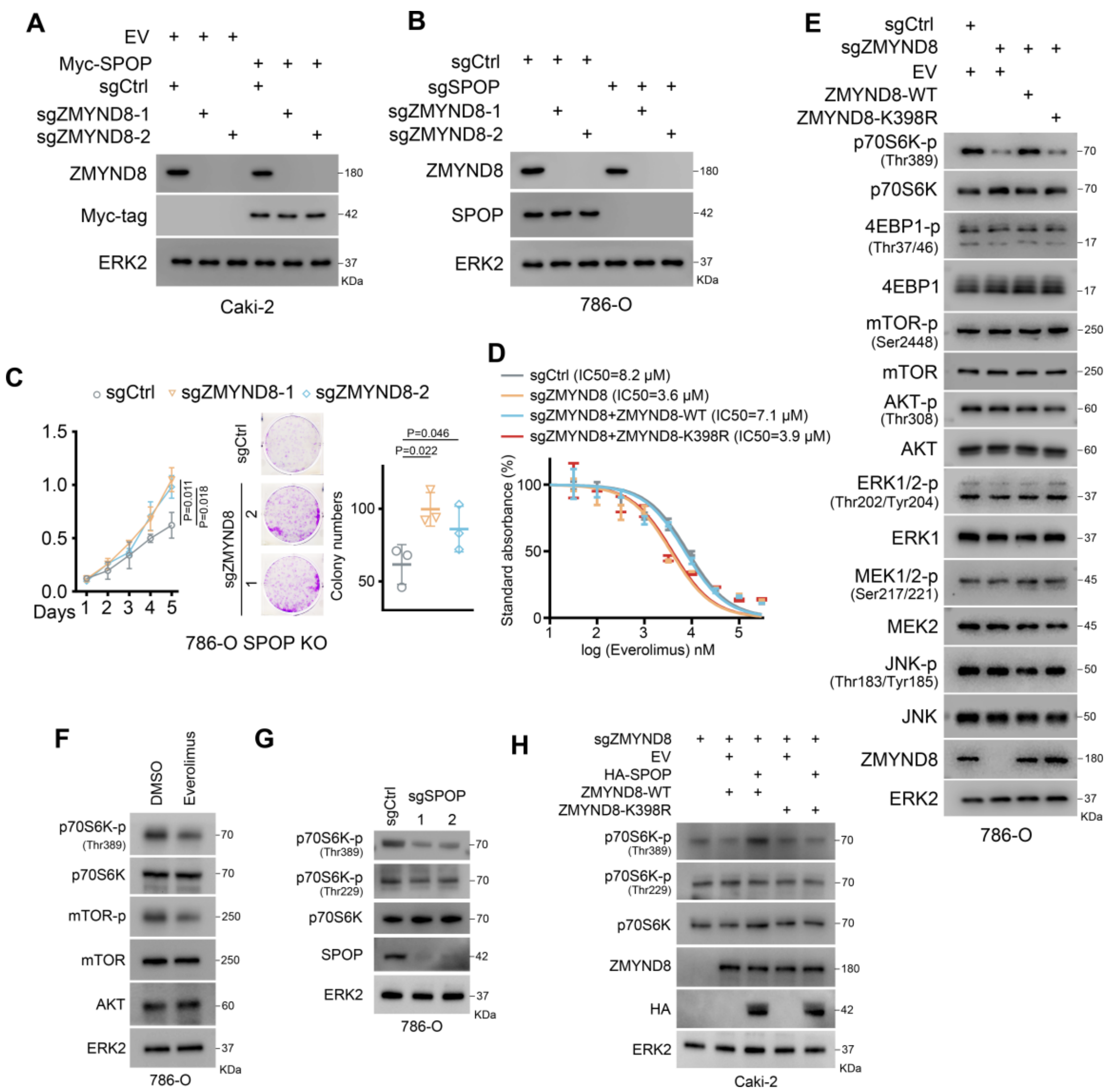

Fig. S4. ZMYND8 Ubiquitination Mediated by SPOP Promotes ccRCC Cell Proliferation and p70S6K Phosphorylation Independently of mTOR. (Supp to Fig. 3)

(A and B) WB analysis of indicated proteins in ccRCC cell lines transfected with the indicated plasmids.

(C) Measurement of proliferation by MTS and colony formation assay in 786-O SPOP KO cells infected with lentivirus expressing the indicated sgRNAs. Two-tailed unpaired Student's t-test; *P* values arranged in order: 0.011, 0.018, 0.046, 0.022.

(D) 72h IC50 of everolimus in 786-O cells transfected with indicated sgZMYND8 and/or rescued ZMYND8.

(E)WB analysis of indicated proteins in 786-O transfected with indicated plasmids.

(F) WB analysis of indicated proteins in 786-O cells treated with DMSO or everolimus (5μM) for 24h.

(G and H) WB analysis of indicated proteins in 786-O(G) and Caki-2(H) transfected with indicated plasmids.

WB analyses (A, B, E-H) are representative of three independent experiments with similar results. MTS assays (C, D) and colony formation assays (C) are obtained in three biological replicates. Data are shown as mean ± SD (C, D). Source data are provided as a Source Data file.

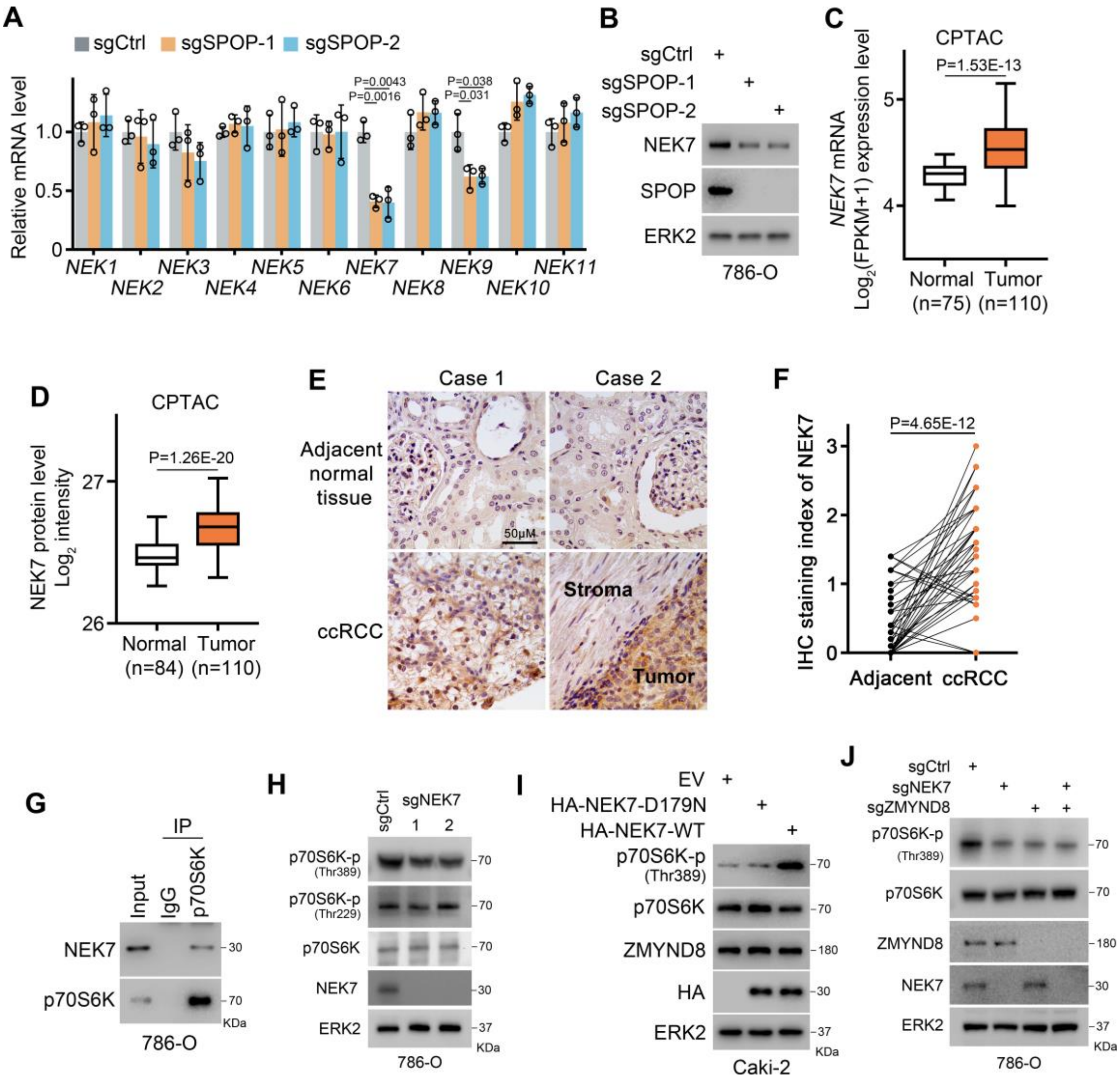

Fig. S5. ZMYND8 Ubiquitination Promotes p70S6K Phosphorylation through NEK7. (Supp to Fig. 4)

(A) RT-qPCR analysis of mRNA expression of NEK family members in mock or SPOP KO 786-O cells. Data are shown as mean±SD derived from three biological replicates. Two-tailed unpaired Student's t-test; *P* values arranged in order: 0.0043, 0.0016, 0.038, 0.031.

(B) WB analysis of indicated proteins in mock or SPOP KO 786-O cells.

(C) Meta-analysis of NEK7 mRNA level in the CPTAC cohort of normal and ccRCC patient specimens. Two-tailed unpaired Student's t-test; *P* = 1.53E-13.

(D) Meta-analysis of NEK7 protein level in the CPTAC cohort of normal and ccRCC patient specimens. Two-tailed unpaired Student's t-test; *P* = 1.26E-20.

(E) Representative ZMYND8 IHC staining images in a paired benign and human ccRCC on TMA.

(F) Quantification of ZMYND8 protein level in benign kidney tissues and ccRCC specimens (n=50 patients paired cores). Paired t test. Two-tailed paired Student's t-test; P = 4.65E-12.

(G) WB analysis of co-IP samples of IgG or anti-p70S6K antibody in WCL of 786-O cells.

(H) WB analysis of indicated protein in sgControl or sgNEK7 786-O cells.

(I) WB analysis of indicated protein in Caki-2 cells transfected with indicated plasmids.

(J) WB analysis of indicated protein in sgZMYND8 and/or sgNEK7 KO 786-O cells.

WB analyses (B, G-J) are representative of three independent experiments with similar results. Source data are provided as a Source Data file.

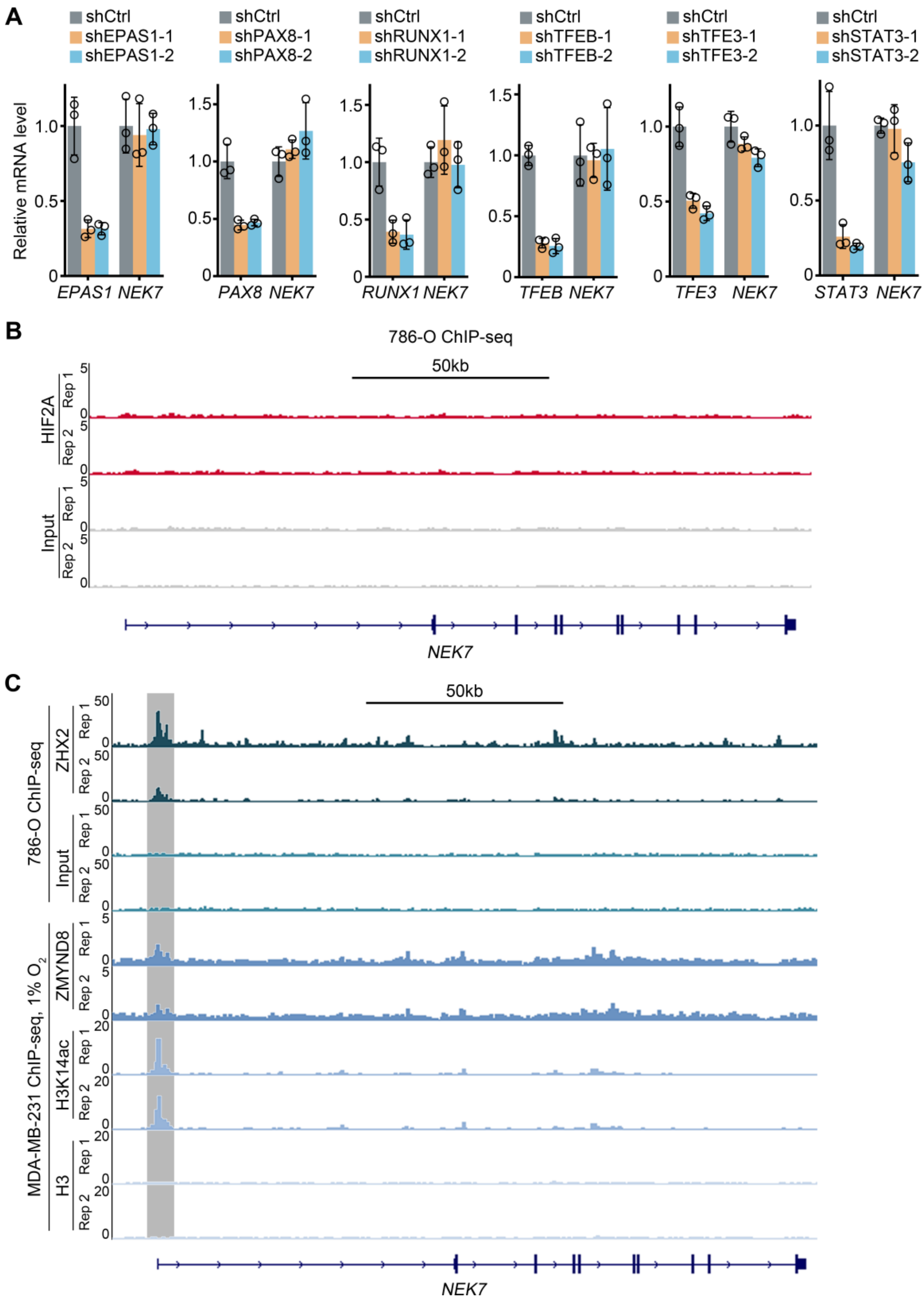

Fig. S6. Ubiquitinated ZMYND8 Promotes NEK7 Transcription by Forming a Protein Complex with ZHX2. (Supp to Fig. 5)

(A) RT-qPCR analysis of indicated genes and NEK7 mRNA expression in mock or gene KD 786-O cells. Data are shown as mean±SD derived from three biological replicates.

(B) UCSC genome browser screen shot of ChIP-seq signal of input control and HIF2A in the *NEK7* gene locus[1].

(C) UCSC genome browser screen shot of ChIP-seq signal of ZHX2, ZMYND8, H3K14ac and H3 (as control) in the *NEK7* gene locus[2, 3].

Source data are provided as a Source Data file.

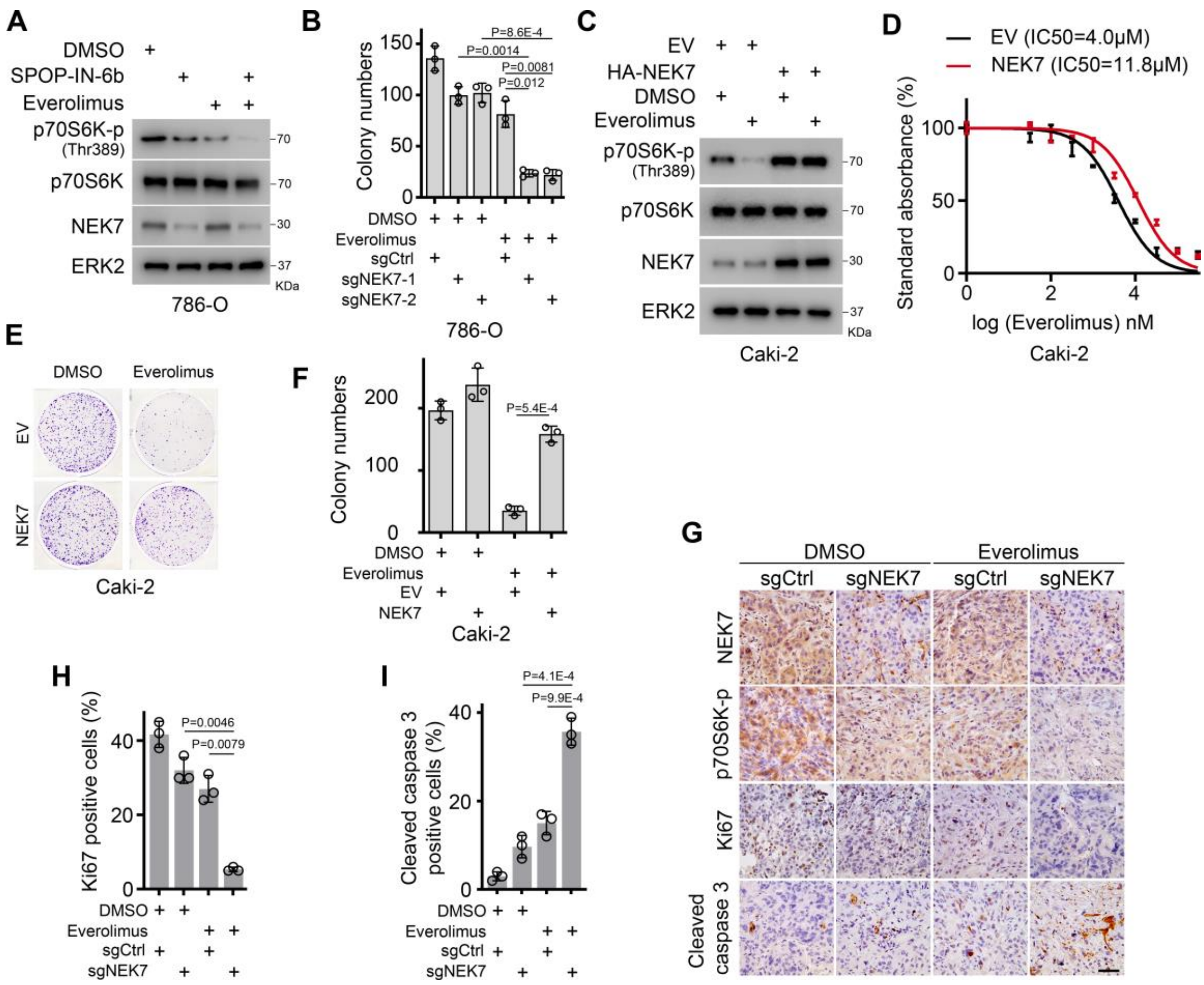

Fig. S7. NEK7 Inhibition Augments the Anti-cancer Efficacy of mTOR Inhibitor. (Supp to Fig. 6)

(A) WB analysis of indicated proteins in 786-O cells treated with SPOP-IN-6b (2 μM) and/or everolimus (5 μM).

(B) Quantification of colony formation from Fig. 6C. *P* values arranged in order: 8.6E-4, 0.0014, 0.0081, 0.012.

(C)WB analysis of indicated proteins in control or NEK7-overexpressing Caki-2 cells treated with DMSO or everolimus (5 μM).

(D) IC50 of everolimus in control or NEK7-overexpressing Caki-2 cells treated with different concentrations of everolimus for 72 h.

(E and F) Colony formation assay of control or NEK7-overexpressing Caki-2 cells treated with DMSO or everolimus (5 μM) followed by photograph (E) and quantification (F). *P* = 5.4E-4.

(G) IHC staining for NEK7, p70S6K-p, Ki67, and cleaved Caspase-3 were performed. Representative images were taken from each group. IHC staining are representative of three independent experiments with similar results. Scale bars are indicated in the images. Scale bar: 50 μm.

(H-I) Ki67 (H) and cleaved Caspase-3 (I) positive cells in tissues obtained from (G) were quantified. The number of positive cells from at least five fields was counted and analyzed. Data are shown as mean±SD (n=3 randomly selected fields per mouse group). *P* values arranged in order: 0.0046, 0.0079, 4.1E-4, 9.9E-4.

WB analyses (A, C) are representative of three independent experiments with similar results. MTS assays (D) and colony formation assays (B, E, F) are obtained in three biological replicates. Data are shown as mean ± SD (B, D, F). Statistics: Two-tailed unpaired Student's t-test are used in (B, F, H, I). Source data are provided as a Source Data file.

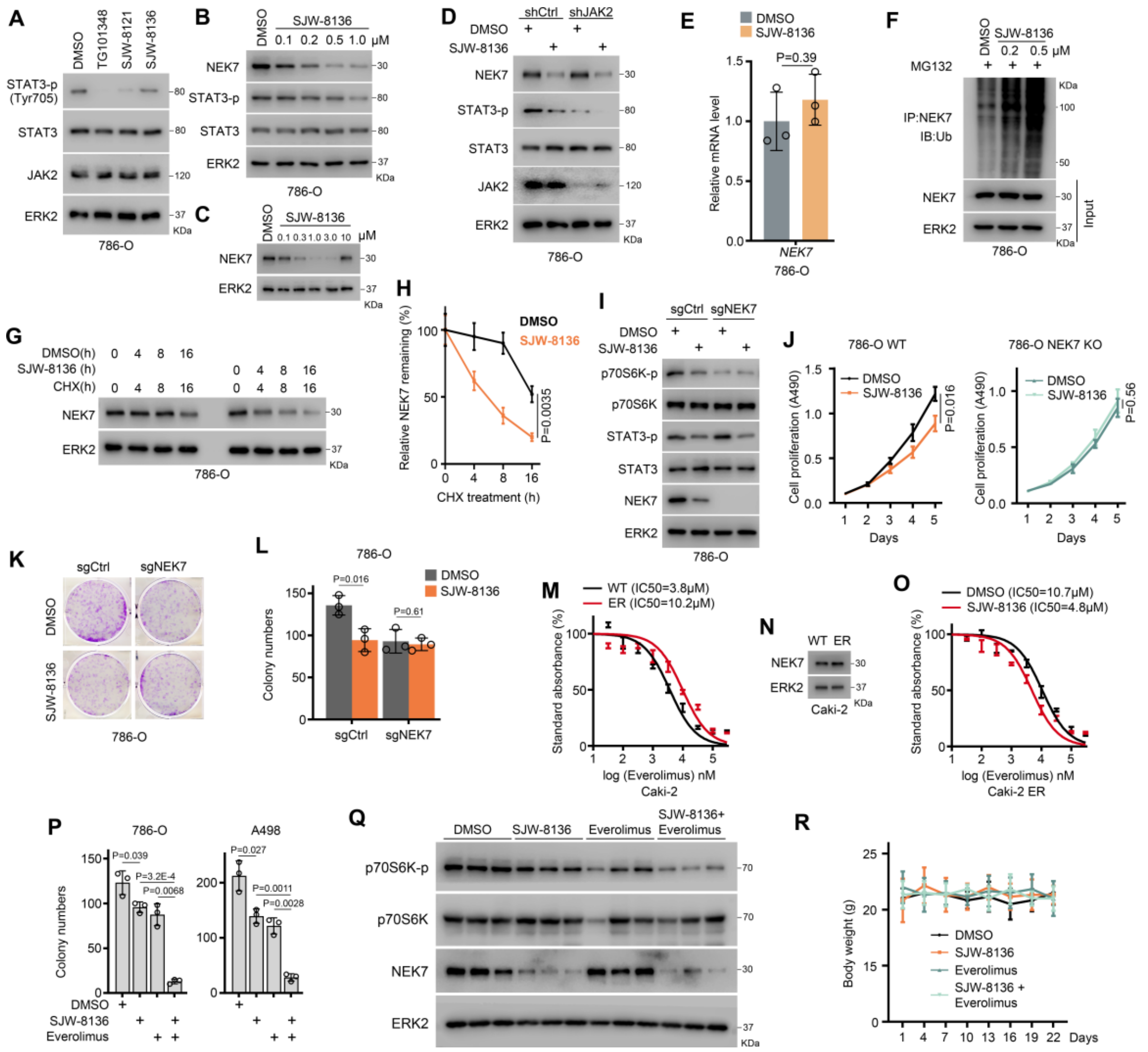

Fig. S8. Co-administration of NEK7 Degrader and mTOR Inhibitor Effectively Inhibits ccRCC Tumor Growth. (Supp to Fig. 6)

(A) WB analysis of STAT3-p protein level in 786-O cells treated with indicated small molecules (1 μM) for 24 h.

(B and C) WB analysis of indicated protein level in 786-O cells treated with a series of concentrations of SJW-8136 for 24 h.

(D) WB analysis of indicated proteins in mock or JAK2 KD 786-O cells treated with DMSO or SJW-8136 (0.5 μM) for 24 h.

(E) RT-qPCR analysis of NEK7 mRNA expression in 786-O cells treated with DMSO or SJW-8136 (0.5 μM) for 24 h. *P* = 0.39.

(F) WB analysis of co-IP samples in 786-O cells treated with DMSO or SJW-8136 (0.2 or 0.5 μM) for 24 h in the presence of MG132 (20 μM) for the last 12h before samples were harvested.

(G and H) WB analysis(G) and NEK7 normalized quantification(H) of indicated proteins in 786-O cells treated with DMSO, SJW-8136 (1 μM) and/or CHX (50 μg/ml) for the indicated periods of time. *P* = 0.0035.

(I) WB analysis of indicated proteins in mock or NEK7 KO 786-O cells treated with SJW-8136 (0.5 μM) for 24 h.

(J) MTS assay in mock (left) and NEK7 KO (right) 786-O cells treated with SJW-8136 (0.5 μM) for the indicated periods of time. *P* values arranged in order: 0.016, 0.56.

(K and L) Colony formation assay of mock or NEK7 KO 786-O cells treated with SJW-8136 (0.5 μM) followed by photograph (K) and quantification (L). *P* values arranged in order: 0.016, 0.61.

(M) IC50 of everolimus in everolimus sensitive (WT) or resistant (ER) Caki-2 cells.

(N) WB analysis of indicated protein in Caki-2 everolimus sensitive (WT) or resistant (ER) cells.

(O) IC50 of everolimus in Caki-2 ER cells treated with DMSO or SJW-8136.

(P) Quantification of colony formation from Fig. 6N. *P* values arranged in order: 0.039, 3,2E-4, 0.0068, 0.027, 0.0011, 0.0028.

(Q)WB analysis of indicated protein in 786-O ccRCC tumors isolate from the end of experiment (n=3 mice randomly selected for each group).

(R) Measurement of body weight of mice bearing 786-O ccRCC tumors after 22 days of indicated treatment. Data are shown as mean±SD (n=5 mice per group).

WB analyses (A-D, F, G, H, I, N) are representative of three independent experiments with similar results. RT-qPCR(E), MTS assays (J, M, O) and colony formation assays (K, L) are obtained in three biological replicates. Data are shown as mean ± SD (E, H, J, L, M, O, P). Statistics: Two-tailed unpaired Student's t-test are used in (E, H, J, L, P). Source data are provided as a Source Data file.

Supplementary Table 1. sgRNA & shRNA Information

sgRNA

Name	Sequence
sgSPOP-1	CAAGCTTACCCTCTTCTGCG
sgSPOP-2	GTCATCAGGGAGAAGCCCGT
sgBRD2-1	CCAGCTGCAATACCTACACA
sgBRD2-2	CTTGTTGTAAATGTAACAGT
sgBRD4-1	CACCAAACTCCTGAGCATCA
sgBRD4-2	AGTCGAACTGTCACTGTCCG
sgCHD3-1	TACAAGTATGAGCGCATCGA
sgCHD3-2	TGATCAGCTCATACGATGTC
sgCAPRIN1-1	CGACAAGAAACTTCGGAACC
sgCAPRIN1-2	GGTTCCGAAGTTTCTTGTCG
sgZMYND8-1	GACTTAGCGTGATAAACCCG
sgZMYND8-2	GGAGCGCGGCATATCCGACA
sgSETD2-1	AGAGTTTAAAGCTCGAGTGA
sgSETD2-2	GGACTGTGAACGGACAACTG
sgDAXX-1	TGCACTATCAGATCCTGTGT
sgDAXX-2	GCGCTGCTCTATGACACGGC
sgINF2-1	TGCGCGCCGTCATGAACTCG
sgINF2-2	GGAGGTCGCTGCTATGATCC
sgGLI3-1	ATCTAGTAGCCCTACGTATC
sgGLI3-2	ACAGAGGGCTCCGCCACGTG
sgNEK7-1	ACAATGGATGAGCAATCACA
sgNEK7-2	TGAAAAACCGACCAAGCCCA

shRNA

Name	Catolog No.	Company
shSRRM1-1	TRCN0000000131	Merck
shSRRM1-2	TRCN0000000132	Merck
shZHX2-1	TRCN0000017743	Merck
shZHX2-2	TRCN0000017745	Merck
shEPAS-1	TRCN0000003803	Merck
shEPAS-2	TRCN0000003804	Merck
shPAX8-1	TRCN0000021275	Merck
shPAX8-2	TRCN0000021278	Merck
shRUNX1-1	TRCN0000013659	Merck
shRUNX1-2	TRCN0000013660	Merck
shTFEB-1	TRCN0000013109	Merck
shTFEB-2	TRCN0000013111	Merck
shTFE3-1	TRCN0000019980	Merck
shTFE3-2	TRCN0000019982	Merck
shSTAT3-1	TRCN0000020840	Merck
shSTAT3-2	TRCN0000020843	Merck
shJAK2-1	TRCN0000003179	Merck
shJAK2-2	TRCN0000003180	Merck

Supplementary Tables 2. Reagent and Resource

Antibodies	Cat No.	Company	Dilution
SPOP	16750-1-AP	Proteintech	1:2000
ZMYND8	A302-089A	Bethyl Lab Inc.	1:1000
BRD2	ab139690	Abcam	1:1000
CHD3	ab109195	Abcam	1:1000
CAPRIN1	15112-1-AP	Proteintech	1:1000
SETD2	55377-1-AP	Proteintech	1:1000
BRD4	ab128874	Abcam	1:1000
GLI3	ab181130	Abcam	1:1000
DAXX	ab32140	Abcam	1:1000
INF2	ab245555	Abcam	1:1000
SRRM1	ab221061	Abcam	1:1000
Phospho-p70S6K (Thr389)	9205	CST	1:500
p70S6K	9202	CST	1:1000
Phospho-4E-BP1 (Thr37/46)	2855	CST	1:500
4E-BP1	9644	CST	1:1000
Phospho-mTOR (Ser2448)	5536	CST	1:500
mTOR	2983	CST	1:1000
Phospho AKT (Thr308)	9275	CST	1:500
AKT	SC-5298	Santa Cruz	1:1000
Phospho ERK1/2 (T202/Y204)	4370S	CST	1:500
ERK2	SC-1647	Santa Cruz	1:5000
ERK1	SC-93	Santa Cruz	1:5000
Phospho MEK1/2 (Ser217/221)	9121	CST	1:500
MEK2	9125	CST	1:1000
Phospho SAPK/JNK (Thr183/Ty185)	9251	CST	1:500
SAPK/JNK	9252	CST	1:1000
Myc tag	SC-40	Santa Cruz	1:2000
FLAG tag	66008-3-IG	Proteintech	1:2000
Ubiquitin	3936	CST	1:1000
GFP tag	50430-2-AP	Proteintech	1:1000
HA tag	66006-2-IG	Proteintech	1:2000
ZHX2	GTX112232	Genetex	1:1000
Pan-Methylation antibody	ab7315	Abcam	1:1000
NEK7	A302-684A	Bethyl Lab Inc.	1:1000
STAT3	9139	CST	1:1000
Phospho STAT3 (Tyr705)	9145	CST	1:500
JAK2	sc-390539	Santa Cruz	1:1000
Ki67	ab15580	Abcam	1:5000
Cleaved Caspase-3	9579	CST	1:1000

Critical Commercial Assays	Cat No.	Company
KOD Plus Mutagenesis Kit	Cat#F0936K	Toyobo
In vitro transcription/translation System	Cat#L1170	Promega

Supplementary Tables 3. Oligonucleotide Sequence

**RT-qPCR primers**		
ZMYND8	Forward	GGGTTTATCACGCTAAGTGTCTG
	Reverse	GGCTTTACTCTGGGTCTCGATG
NEK1	Forward	GGCCAGGGAACAAGGATGGAGGA
	Reverse	TGCCTTTCTGGGAGCCATCGAC
NEK2	Forward	CGGAAGTTCCTGTCTCTGGCA
	Reverse	TTCAGGTCCTTGCACTTGGACT
NEK3	Forward	TGAGAATGATTGGGGAGG
	Reverse	GGGTGTTTCATTTTGGCTA
NEK4	Forward	ACATCATCAAAGTAGGGGACCT
	Reverse	CAGGGCTCATGTAGTAGGGTG
NEK5	Forward	GCCTTCGGGAAAGCATACTTAG
	Reverse	AGGCTACAATGTTGGGATGTT
NEK6	Forward	AGAAGATCGAGCAGTGTGACTAC
	Reverse	TAGCATAAGCCTTGACAGGTCAC
NEK7	Forward	GCCTTACGACCGGATATGGG
	Reverse	CACTAAATTGTCCGCGACCAA
NEK8	Forward	GCCTCAAGAGGGCTTTCGA
	Reverse	AAGGTGCCACTCATGATCTTCAG
NEK9	Forward	TACGAGCGACACTGCGATTC
	Reverse	ACGCGGATGGGGATGTAGT
NEK10	Forward	ATGAGGGATCCATGTTATCAGGA
	Reverse	TGGGGCTCTGCACAAAGTA
NEK11	Forward	AGAGGATGCCACATCTGACC
	Reverse	TTCTGCATGGCTGATCTAGA
**ChIP-qPCR primers**		
NEK7	Forward	AGGCATGGGACTGGTCATTG
	Reverse	GGTTGGAATCCCAGCCAAGA

Supplementary Materials and Methods

In vitro droplet assays and FRAP assays

Recombinant mEGFP-ZMYND8 were stored in 1 × PBS, pH 7.4, supplemented with 360 mM NaCl. For in vitro droplet assays, protein was diluted in filtered distilled water into the indicated protein concentration and NaCl concentration. The protein solution was immediately loaded either into a 384-well plate or a homemade chamber comprising a glass slide with a coverslip attached by two parallel strips of tape.

FRAP assays were performed using a Leica DMI8 confocal microscope. In the presence of 150 mM NaCl and 10% PEG 8000, droplets were formed with 30 μM recombinant mEGFP-ZMYND8. A bleached area with a diameter of approximately 2 μm was selected and bleached three times using 80% of the maximum laser power of the 488-nm laser, and time-shifted images were acquired at 2 second intervals over a 2-minute time course. The fluorescence intensity of the region of interest was corrected by the unbleached control region, the pre-bleached intensity of the region of interest was normalized to 1, and the mean and standard deviation of the recovery time were calculated.

Establishment of everolimus-resistant sublines.

Continuous exposure to everolimus was performed using previously described methods[4]. In brief, Caki-2 cells were cultured in medium containing 1 μM everolimus. Three months later, the everolimus-exposed cells were designated as Caki2-ER. Caki2-ER cells were maintained in a similar manner to Caki-2 cells, respectively, except that the medium contained 1 μM everolimus.

In vitro ubiquitination assay

In vitro ubiquitination assays were carried out using a protocol reported previously[5, 6]. Briefly, 2 μg APP-BP1/Uba3, 2 μg His-UBE2M enzymes and 5 μg NEDD8 were incubated at 30°C for 2 h in the presence of ATP. The thioester loaded His-UBE2M-NEDD8 was further incubated with 3 μg His-DCNL2, 6 μg CUL3/RBX1 at 4°C for 2

h to obtain the NEDDylated CUL3/RBX1. The NEDDylated CUL3/RBX1, 5 µg GST-SPOP, 5 µg Ub, 500 ng E1, 750 ng E2 (UbcH5a and UbcH5b), and 5 µg His-ZMYND8 were incubated with 0.6 µl 100 mM ATP, 1.5 µl 20 µM ubiquitin aldehyde, 2 µl 10 × ubiquitin reaction buffer (500 mM Tris-HCl (pH7.5), 50 mM KCl, 50 mM NaF, 50 mM MgCl2 and 5 mM DTT), 2 µl 10 × energy regeneration mix (200 mM creatine phosphate and 2 µg/µl creatine phosphokinase), 2 µl 10 × protease inhibitor cocktail at 30°C for 2 h, followed by western blot analysis. The Ub, E1, E2 and CUL3/RBX1 were purchased from UBIQUIGENT.

Chemical synthesis.

Compound 22 was synthesized according to the reported procedures[7, 8].

Scheme 1[a].

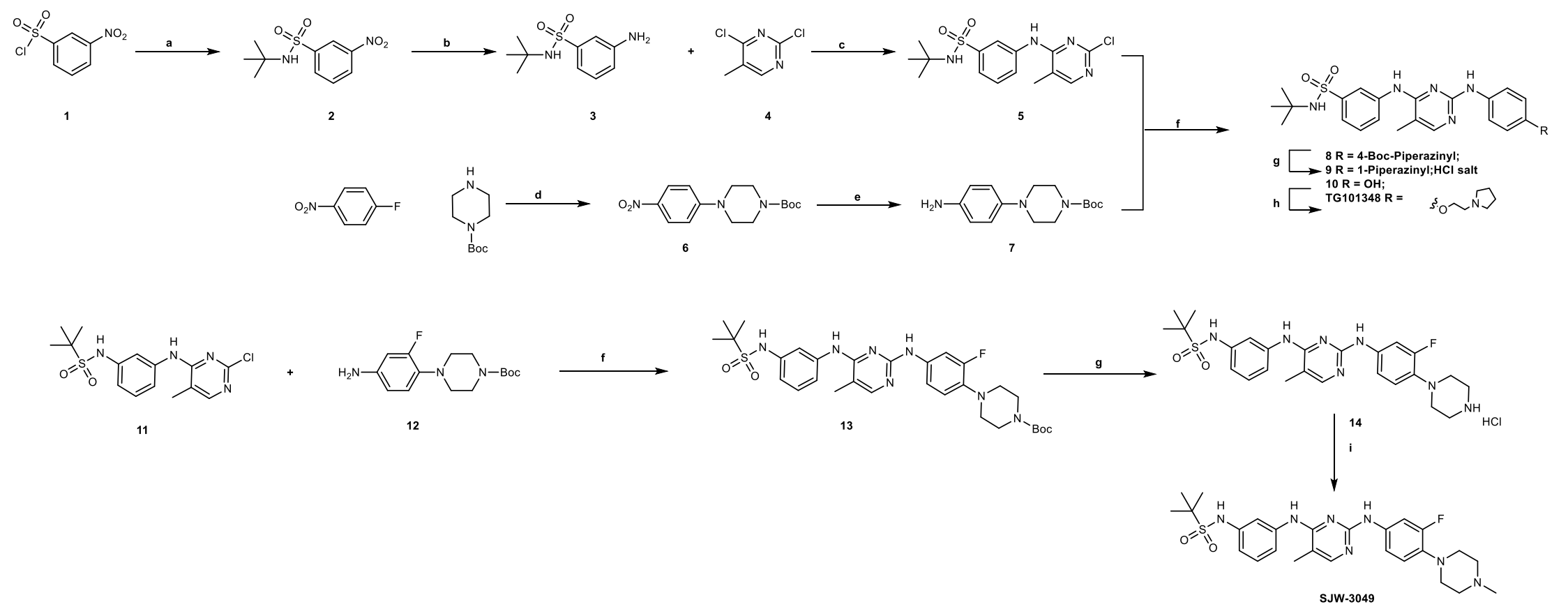

[a]Reagent and conditions: (a) *tert*-Butylamine, TEA, DCM; (b) Zn, NH_4Cl, dioxane/H_2O; (c) MeOH/H_2O, 45ºC; (d) K_2CO_3, DMF; (e) Pd/C, H_2, MeOH; (f) 7, or 4-Aminophenol, $Pd_2(dba_3)_2$, XantPhos, Cs_2CO_3, Dioxane; (g) 4M HCl in dioxane; DCM; (h) 1-(2-chloroethyl)pyrrolidine hydrochloride, KOH, DMF, 70ºC, 15h; (i) HCHO, $NaBH(OAc)_3$, DCM.

N-(*tert*-butyl)-3-nitrobenzenesulfonamide (2). Compound 1 (15.0 g, 65 mmol) was dissolved in DCM (80 mL) and cooled to 0°C, then TEA (27 mL, 195 mmol) and *tert*-butylamine (13.65 mL, 130 mmol) were added. The mixture was stirred at 0°C for 1h, then stirred at room temperature overnight. The reaction solution was washed with $NaHCO_3$ solution. The organic phase was dried with Na_2SO_4, filtered and concentrated, giving desired product (14.3 g, 85%) as yellow solid. ^{1}H NMR (400 MHz, $CDCl_3$) δ 8.74 (t, *J* = 1.8 Hz, 1H), 8.40 (ddd, *J* = 8.2, 2.2, 0.9 Hz, 1H), 8.25 – 8.20 (m, 1H), 7.71 (t, *J* = 8.0 Hz, 1H), 4.74 (s, 1H), 1.27 (s, 9H). 79.

3-amino-N-(*tert*-butyl)benzenesulfonamide (3). Compound 2 (14.0 g, 54.2 mmol) was dissolved in dioxane/H_2O (100 mL, 4:1) and cooled to 0°C, then NH_4Cl (17.36 g, 325.2 mmol) and zinc powder (21.1 g, 325.2 mmol) were added. The mixture was stirred at 0°C for 1h, then stirred at room temperature overnight. Solid was filtered off and washed with dioxane (50 mL). The filtrate was concentrated. The residue was dissolved in EA and washed with $NaHCO_3$ solution. The organic phase was dried with Na_2SO_4, filtered and concentrated, giving desired product (12.4 g, 85%) as yellow solid. ^{1}H NMR (400 MHz, $CDCl_3$) δ 7.70 – 7.61 (m, 4H), 5.05 (s, 1H), 1.62 (d, *J* = 3.8 Hz, 9H).

N-(tert-butyl)-3-((2-chloro-5-methylpyrimidin-4-yl)amino)benzenesulfonamide (5). Compound 3 (3.26 g, 20 mmol) and compound 4 (2.28 g, 10 mmol) were mixed in MeOH/H_2O (30 mL, 1:1) and heated 45 °C overnight. Solid was formed. The

reaction solution was cooled to room temperature, then the solid was filtered, washed with MeOH, and dried, giving desired product (1.3 g, 37%) as white solid. ^{1}H NMR (400 MHz, DMSO-d_6) δ 9.11 (s, 1H), 8.12 (d, J = 7.4 Hz, 2H), 7.87 (m, 1H), 7.58 – 7.54 (m, 2H), 7.51 (s, 1H), 2.20 (s, 3H), 1.14 (s, 9H).

***tert*-Butyl 4-(4-nitrophenyl)piperazine-1-carboxylate (6).** 1-fluoro-4-nitrobenzene (4.23 g, 30 mmol), *tert*-butyl piperazine-1-carboxylate (6.70 g, 36 mmol) and K_2CO_3 (6.20 g, 45 mmol) were mixed and stirred at rt overnight. H_2O was added to the reaction, then yellow solid was formed. The desired product (9.2 g, 100%) was obtained by filtration and washing with H_2O. ^{1}H NMR (400 MHz, $CDCl_3$) δ 8.14 (d, J = 9.4 Hz, 2H), 6.82 (d, J = 9.4 Hz, 2H), 3.60 (m, 4H), 3.45 – 3.38 (m, 4H), 1.49 (s, 9H).

***tert*-Butyl 4-(4-aminophenyl)piperazine-1-carboxylate (7).** To a methanol solution of Compound 6 (5.8 g, 18.87 mmol) was added 10% palladium on carbon (1.0 g). The suspension was kept under hydrogen atmosphere (1 atm) for 15 h and was then filtered through a pad of celite. The filtrate was concentrated in vacuo, giving compound 7 (5.0 g, 96%). ^{1}H NMR (400 MHz, $CDCl_3$) δ 6.82 (d, J = 8.5 Hz, 2H), 6.65 (d, J = 8.5 Hz, 2H), 3.60 – 3.53 (m, 4H), 2.97 (m, 4H), 1.47 (s, 9H).

***tert*-Butyl 4-(4-((4-((3-(N-(tert-butyl)sulfamoyl)phenyl)amino)-5-methylpyrimidin**

-2-yl)amino)phenyl)piperazine-1-carboxylate (8). To a solution of compound 5 (1.06 g, 3.0 mmol), compound 7 (998 mg, 3.6 mmol) in 1, 4-dioxane (20 mL) was added palladium $Pd_2(dba)_3$ (274 mg, 0.3 mmol), XantPhos (347 mg, 0.6 mmol), cesium carbonate (2.44 g, 7.5 mmol). The resulting mixture was stirred at 100 °C for 12 h under nitrogen atmosphere. The mixture was diluted with EA, washed with water. The organic layer was dried over anhydrous sodium sulphate and concentrated. The residue was purified by column chromatography (Hex: EA = 50: 50) to give product (1.1 g, 61%). ^{1}H NMR (400 MHz, DMSO-d_6) δ 8.75 (s, 1H), 8.52 (s, 1H), 8.14 (m, 2H), 7.90 (s, 1H), 7.57 (s, 1H), 7.49 (m, 4H), 6.84 (d, J = 8.7 Hz, 2H), 3.45 (m, 4H), 3.03 – 2.91 (m, 4H), 2.12 (s, 3H), 1.42 (s, 9H), 1.12 (s, 9H).

N-(*tert*-butyl)-3-((5-methyl-2-((4-(piperazin-1-yl)phenyl)amino)pyrimidin-4-yl)amino)benzenesulfonamide (9). Compound 8 (1.0 g, 1.68 mmol) was dissolved in DCM, then HCl (4M in dioxane, 4.2 mL, 16.8 mmol) were added. The mixture was stirred at RT overnight. The solvent was removed, giving product as hydrogen chloride salt (890 mg, 100%). ^{1}H NMR (400 MHz, DMSO-d_6) δ 10.22 (s, 1H), 9.81 (s, 1H), 9.14 (s, 2H), 7.97 (s, 1H), 7.90 (s, 2H), 7.69 (d, J = 8.0 Hz, 1H), 7.63 (s, 1H), 7.56 (t, J = 7.9 Hz, 1H), 7.28 (d, J = 8.8 Hz, 2H), 6.94 (d, J = 8.7 Hz, 2H), 3.32 (m, 4H), 3.21 (m, 4H), 2.17 (s, 3H), 1.10 (s, 9H).

N-(*tert*-butyl)-3-((2-((4-hydroxyphenyl)amino)-5-methylpyrimidin-4-yl)amino)benzenesulfonamide (10). Compound 5 (1.06 g, 3.0 mmol) and 4-

aminophenol (318 mg, 3.0 mmol) were mixed in *n*-butanol (10 mL), and 3 drops of HCl was added. The mixture was heated to 80ºC in sealed tube for overnight. The reaction was cooled to RT, then the solid was filtered and washed with *n*-butanol, giving product (715 mg, 56%). ^{1}H NMR (400 MHz, DMSO-d_6) δ 10.09 (s, 1H), 9.79 (s, 1H), 9.45 (s, 1H), 7.93 (s, 2H), 7.81 (s, 1H), 7.65 (d, *J* = 7.9 Hz, 1H), 7.55 (s, 1H), 7.48 (t, *J* = 8.1 Hz, 1H), 7.12 (d, *J* = 8.8 Hz, 2H), 6.69 (d, *J* = 8.5 Hz, 2H), 2.14 (s, 3H), 1.07 (s, 9H).

N-(tert-butyl)-3-((5-methyl-2-((4-(2-(pyrrolidin-1-yl)ethoxy)phenyl)amino) pyrimidin-4-yl)amino)benzenesulfonamide (TG101348). Compound 10 (85 mg, 0.2 mmol), 1-(2-chloroethyl)pyrrolidine hydrochloride (41 mg, 0.24 mmol) and KOH (23 mg, 0.4 mmol) were mixed and dissolved in DMF (4 mL). the mixture was heated to 70ºC for 15h. The reaction was purified by column chromatography (DCM: MeOH (2% ammonia) = 95:5) to give product (25 mg, 24%). ^{1}H NMR (400 MHz, DMSO-d_6) δ 8.78 (s, 1H), 8.54 (s, 1H), 8.11 (s, 2H), 7.90 (s, 1H), 7.58 (s, 1H), 7.53 (s, 1H), 7.51 (s, 1H), 7.48 (d, *J* = 5.0 Hz, 2H), 6.79 (d, *J* = 8.9 Hz, 2H), 4.00 (t, *J* = 5.9 Hz, 2H), 2.75 (t, *J* = 5.9 Hz, 2H), 2.52 (m, 4H), 2.12 (s, 3H), 1.68 (m, 4H), 1.12 (s, 9H).

***tert*-Butyl 4-(4-((4-((3-((1,1-dimethylethyl)sulfonamido)phenyl)amino)-5-methyl-pyrimidin-2-yl)amino)-2-fluorophenyl)piperazine-1-carboxylate (13).** Compound 13 was synthesized from compound 11 and 12 according to the representative procedure described for the synthesis of 8. ^{1}H NMR (400 MHz, DMSO-d_6) δ 9.59 (s,

1H), 8.94 (s, 1H), 8.40 (s, 1H), 7.89 (s, 1H), 7.65 (d, J = 15.4 Hz, 1H), 7.47 (d, J = 7.2 Hz, 2H), 7.27 (d, J = 8.7 Hz, 1H), 7.22 (t, J = 8.2 Hz, 1H), 7.01 (d, J = 8.0 Hz, 1H), 6.88 (t, J = 9.4 Hz, 1H), 3.45 (m, 4H), 2.84 (m, 4H), 2.10 (s, 3H), 1.42 (s, 9H), 1.28 (s, 9H).

N-(3-((2-((3-fluoro-4-(piperazin-1-yl)phenyl)amino)-5-methylpyrimidin-4-yl) amino)phenyl)-2-methylpropane-2-sulfonamide (14). Compound 14 was synthesized from compound 11 and 12 according to the representative procedure described for the synthesis of 9. ^{1}H NMR (400 MHz, DMSO-d_6) δ 10.70 (s, 1H), 9.89 (s, 1H), 9.83 (s, 1H), 9.29 (s, 2H), 7.94 (s, 1H), 7.39 (d, J = 7.7 Hz, 2H), 7.34 – 7.25 (m, 2H), 7.21 (d, J = 7.1 Hz, 1H), 7.09 (d, J = 8.8 Hz, 1H), 6.99 (t, J = 9.1 Hz, 1H), 3.21 (m, 4H), 3.18 (m, 4H), 2.16 (s, 3H), 1.26 (s, 9H).

N-(3-((2-((3-fluoro-4-(4-methylpiperazin-1-yl)phenyl)amino)-5-methylpyrimidin-4-yl)amino)phenyl)-2-methylpropane-2-sulfonamide (SJW-3049). Compound 14 (55 mg, 0.1 mmol) was dissolved in DCM (4 mL). Formaldehyde solution (20 µL, 0.2 mmol) was added, the mixture was stirred at RT for 30 min, then $NaBH(OAc)_3$ (42 mg, 0.2 mmol) was added. The mixture was stirred at RT overnight. The reaction was diluted with DCM and washed with $NaHCO_3$ solution. The organic phase was concentrated and column chromatography (DCM: MeOH (2% ammonia) = 95:5) to give product (35 mg, 66%). ^{1}H NMR (400 MHz, DMSO-d_6) δ 9.58 (s, 1H), 8.88 (s, 1H), 8.38 (s, 1H), 7.88 (s, 1H), 7.63 (d, J = 15.6 Hz, 1H), 7.48 (d, J = 1.8 Hz, 2H),

7.23 (m, 2H), 7.01 (d, J = 8.0 Hz, 1H), 6.85 (t, J = 9.4 Hz, 1H), 2.90 (m, 4H), 2.45 (m, 4H), 2.21 (s, 3H), 2.10 (s, 3H), 1.28 (s, 9H).

Scheme 2[a].

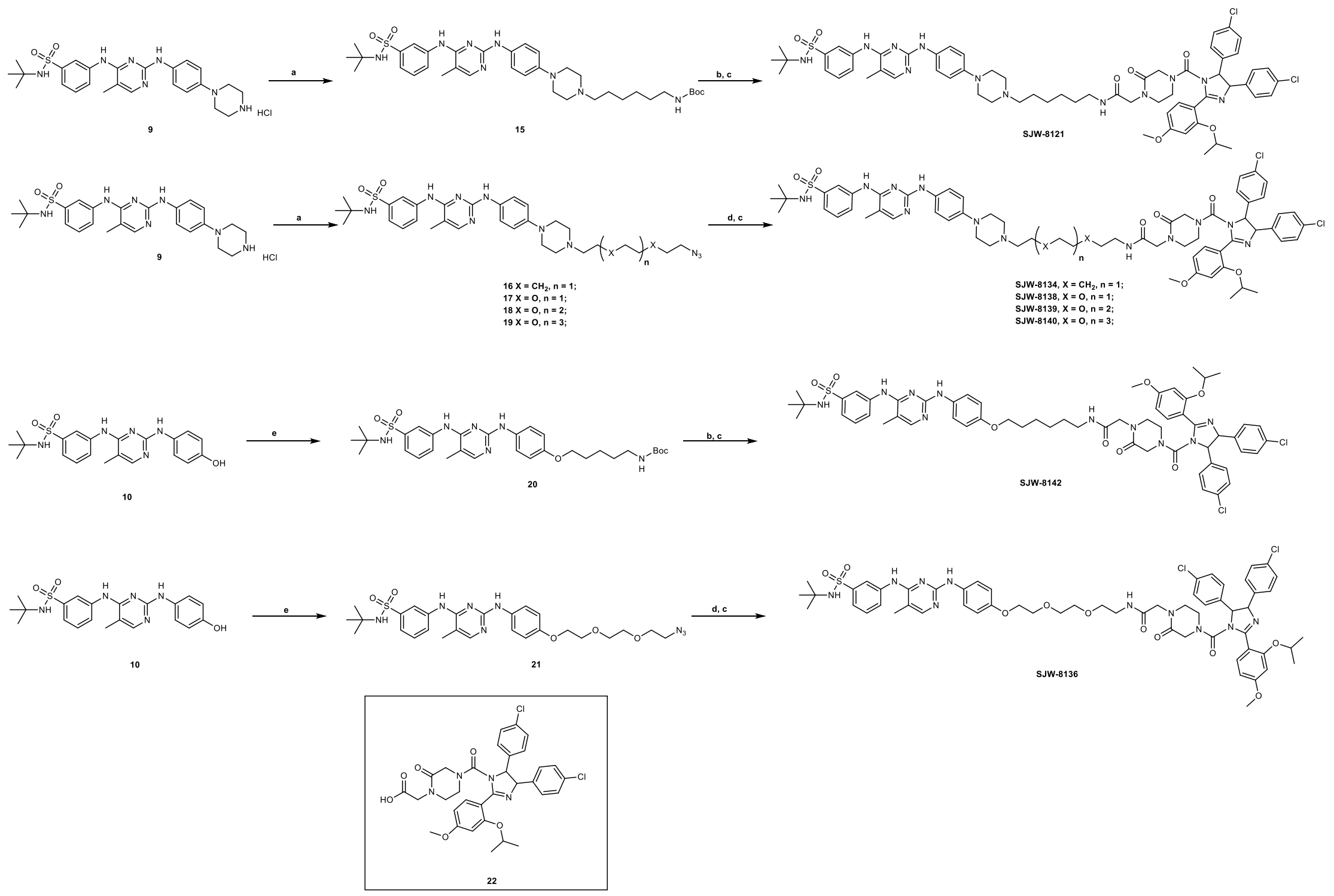

[a]Reagent and conditions: (a) DIPEA, different linkers, DMF, 60°C; (b) HCl, DCM; (c) compound 22, EDCI, HOAt, TEA, DMF; (d) PtO_2, MeOH, H_2; (e) different linkers, Cs_2CO_3, DMF.

***tert*-Butyl (6-(4-(4-((4-((3-((1,1-dimethylethyl)sulfonamido)phenyl)amino)-5-methylpyrimidin-2-yl)amino)phenyl)piperazin-1-yl)hexyl)carbamate (15).**

Compound 9 (106 mg, 0.2 mmol) was dissolved in DMF, then the linker (6-((tert-butoxycarbonyl)amino)hexyl 4-methylbenzenesulfonate, 149 mg, 0.4 mmol), and DIPEA (100 μL, 0.6 mmol) were added. The mixture was heated to 60°C overnight.

The solvent was removed, and the residue was purified by flash chromatography (DCM: MeOH (2% ammonia) = 96:4), giving product (125 mg, 90%). LC-MS (ESI) m/z: $[M+H]^+$695.2

2-(4-(4,5-bis(4-chlorophenyl)-2-(2-isopropoxy-4-methoxyphenyl)-4,5-dihydro-1H-imidazole-1-carbonyl)-2-oxopiperazin-1-yl)-N-(6-(4-(4-((4-((3-(N-(*tert*-butyl) sulfamoyl)phenyl)amino)-5-methylpyrimidin-2-yl)amino)phenyl)piperazin-1-yl)hexyl)acetamide (SJW-8121). Compound 11 (35 mg, 0.05mmol) was dissolved in DCM, then HCl (4M in dioxane, 125 μL, 0.5 mmol) were added. The mixture was stirred at RT for overnight. The solvent was removed, and the residue was dissolved in DMF, then TEA (21 μL, 0.15 mmol), compound 22 (32 mg, 0.05 mmol), EDCI (14 mg, 0.075 mmol) and HOAt (0.6M in DMF, 125 μL, 0.075 mmol) were added. The mixture was stirred at RT overnight. The solvent was removed, and the residue was partitioned between $NaHCO_3$ solution and DCM. The organic layer was separated and concentrated in vacuo. The residue was purified by flash chromatography DCM: MeOH (2% ammonia) = 96:4, giving product (12 mg, 20%). ^1H NMR (400 MHz, $CDCl_3$) δ 8.24 (s, 1H), 7.90 (s, 1H), 7.78 (d, *J* = 8.3 Hz, 1H), 7.59 (d, *J* = 8.5 Hz, 1H), 7.55 (d, *J* = 7.7 Hz, 1H), 7.38 (m, 3H), 7.08 (d, *J* = 8.4 Hz, 2H), 7.02 (d, *J* = 8.4 Hz, 2H), 6.96 – 6.85 (m, 7H), 6.56 – 6.50 (m, 2H), 6.47 (d, *J* = 2.1 Hz, 1H), 6.28 (s, 1H), 5.55 (d, *J* = 9.6 Hz, 1H), 5.47 (d, *J* = 9.6 Hz, 1H), 4.78 (s, 1H), 4.61 (dt, *J* = 12.2, 6.1 Hz, 1H), 3.90 (dd, *J* = 16.3, 10.6 Hz, 2H), 3.85 (s, 3H), 3.68 (dd, *J* = 16.7, 8.9 Hz, 2H), 3.52 (m, 1H), 3.25 (m, 4H), 3.19 (m, 3H), 3.13 (d, *J* = 5.3 Hz, 2H), 2.72 (m, 4H),

2.47 (d, *J* = 7.8 Hz, 2H), 2.12 (s, 3H), 1.58 (m, 2H), 1.51 – 1.44 (m, 2H), 1.38 (d, *J* = 6.0 Hz, 3H), 1.33 (m, 5H), 1.25 (m, 2H), 1.17 (s, 9H). LC-MS (ESI) m/z: $[M+H]^+$1215.3

N-(3-((2-((4-(4-(8-azidooctyl)piperazin-1-yl)phenyl)amino)-5-methylpyrimidin-4-yl)amino)phenyl)-2-methylpropane-2-sulfonamide (16). Compound 16 was synthesized from compound 9 and linker (8-azidooctyl 4-methylbenzenesulfonate) according to the representative procedure described for the synthesis of 15. ^{1}H NMR (400 MHz, $CDCl_3$) δ 8.24 (s, 1H), 7.90 (s, 1H), 7.78 (d, *J* = 8.2 Hz, 1H), 7.57 (d, *J* = 8.0 Hz, 1H), 7.39 (t, *J* = 8.7 Hz, 3H), 6.93 (d, *J* = 8.8 Hz, 2H), 6.87 (s, 1H), 6.47 (s, 1H), 4.56 (s, 1H), 3.31 (m, 4H), 3.26 (t, *J* = 7.0 Hz, 2H), 2.80 (m, 4H), 2.55 (m, 2H), 2.13 (s, 3H), 1.64 – 1.56 (m, 4H), 1.34 (m, 8H), 1.18 (s, 9H). LC-MS (ESI) m/z: $[M+H]^+$649.2

2-(4-(4,5-bis(4-chlorophenyl)-2-(2-isopropoxy-4-methoxyphenyl)-4,5-dihydro-1H-imidazole-1-carbonyl)-2-oxopiperazin-1-yl)-N-(8-(4-(4-((4-((3-(N-(*tert*-butyl)sulfamoyl)phenyl)amino)-5-methylpyrimidin-2-yl)amino)phenyl)piperazin-1-yl)octyl)acetamide (SJW-8134). Compound 16 (40 mg, 0.061 mmol) was dissolved in MeOH. PtO_2 (8 mg) was added. The suspension was kept under hydrogen atmosphere (1 atm) for 2 h and was then filtered through a pad of celite. The filtrate was concentrated in vacuo. The residue was dissolved in DMF, then TEA (26 µL, 0.186 mmol), compound 22 (40 mg, 0.061 mmol), EDCI (17 mg, 0.092 mmol) and

HOAt (0.6M in DMF, 153 μL, 0.092 mmol) were added. The mixture was stirred at RT overnight. The solvent was removed, and the residue was partitioned between $NaHCO_3$ solution and CH_2Cl_2. The organic layer was separated and concentrated in vacuo. The residue was purified by flash chromatography (DCM: MeOH (2% ammonia) = 96:4, giving product (10 mg, 13%). ^{1}H NMR (400 MHz, $CDCl_3$) δ 8.23 (s, 1H), 7.89 (s, 1H), 7.81 – 7.77 (m, 1H), 7.57 (m, 2H), 7.43 – 7.35 (m, 3H), 7.08 (d, *J* = 8.5 Hz, 2H), 7.02 (d, *J* = 8.6 Hz, 2H), 6.96 – 6.90 (m, 4H), 6.87 (d, *J* = 8.4 Hz, 2H), 6.53 (m, 2H), 6.47 (d, *J* = 2.2 Hz, 1H), 6.18 (m, 1H), 5.55 (d, *J* = 9.6 Hz, 1H), 5.47 (d, *J* = 9.7 Hz, 1H), 4.79 (s, 1H), 4.61 (dt, *J* = 12.2, 6.1 Hz, 1H), 3.90 (dd, *J* = 16.6, 9.8 Hz, 2H), 3.85 (s, 3H), 3.68 (dd, *J* = 16.4, 10.9 Hz, 2H), 3.51 (m, 1H), 3.25 (m, 4H), 3.18 (m, 3H), 3.12 (t, *J* = 5.5 Hz, 2H), 2.74 (m, 4H), 2.52 – 2.43 (m, 2H), 2.12 (s, 3H), 1.59 (m, 2H), 1.48 – 1.41 (m, 2H), 1.37 (d, *J* = 6.0 Hz, 3H), 1.33 (d, *J* = 6.0 Hz, 3H), 1.27 (m, 8H), 1.18 (s, 9H). LC-MS (ESI) m/z: $[M+H]^+$1243.3

N-(3-((2-((4-(4-(2-(2-(2-azidoethoxy)ethoxy)ethyl)piperazin-1-yl)phenyl)amino)-5-methylpyrimidin-4-yl)amino)phenyl)-2-methylpropane-2-sulfonamide (17). Compound 17 was synthesized from compound 9 and linker (2-(2-(2-azidoethoxy)ethoxy)ethyl 4-methylbenzenesulfonate) according to the representative procedure described for the synthesis of 15. ^{1}H NMR (400 MHz, $CDCl_3$) δ 8.21 (s, 1H), 7.89 (s, 1H), 7.81 (d, *J* = 8.1 Hz, 1H), 7.56 (d, *J* = 8.1 Hz, 1H), 7.38 (m, 3H), 6.93 (d, *J* = 8.8 Hz, 2H), 6.88 (s, 1H), 6.49 (s, 1H), 4.60 (s, 1H), 3.70 – 3.63 (m, 8H),

3.44 – 3.36 (m, 2H), 3.22 (m, 4H), 2.74 (m, 6H), 2.12 (s, 3H), 1.18 (s, 9H). LC-MS (ESI) m/z: $[M+H]^+$653.1

2-(4-(4,5-bis(4-chlorophenyl)-2-(2-isopropoxy-4-methoxyphenyl)-4,5-dihydro-1H-imidazole-1-carbonyl)-2-oxopiperazin-1-yl)-N-(2-(2-(2-(4-(4-((4-((3-(N-(tert-butyl)sulfamoyl)phenyl)amino)-5-methylpyrimidin-2-yl)amino)phenyl)piperazin-1-yl)ethoxy)ethoxy)ethyl)acetamide (SJW-8138). Compound SJW-8138 was synthesized from compound 17 and 22 according to the representative two-step procedure described for the synthesis of SJW-8134. ^{1}H NMR (400 MHz, $CDCl_3$) δ 8.21 (t, *J* = 1.8 Hz, 1H), 7.88 (s, 1H), 7.77 (dd, *J* = 8.1, 1.4 Hz, 1H), 7.58 (d, *J* = 8.5 Hz, 1H), 7.53 (t, *J* = 6.1 Hz, 1H), 7.41 – 7.33 (m, 3H), 7.10 – 7.04 (m, 3H), 7.01 (d, *J* = 8.6 Hz, 2H), 6.97 – 6.85 (m, 7H), 6.57 (s, 1H), 6.52 (dd, *J* = 8.5, 2.2 Hz, 1H), 6.46 (d, *J* = 2.2 Hz, 1H), 5.54 (d, *J* = 9.6 Hz, 1H), 5.46 (d, *J* = 9.6 Hz, 1H), 4.96 (s, 1H), 4.59 (dt, *J* = 12.1, 6.0 Hz, 1H), 3.96 (d, *J* = 15.7 Hz, 1H), 3.86 (m, 4H), 3.78 (d, *J* = 15.6 Hz, 1H), 3.71 – 3.65 (m, 3H), 3.60 (m, 4H), 3.53 (t, *J* = 5.0 Hz, 2H), 3.47 (m, 1H), 3.41 (m, 2H), 3.25 (m, 1H), 3.21 (m, 4H), 3.13 – 3.05 (m, 2H), 2.76 (m, 4H), 2.71 (t, *J* = 5.2 Hz, 2H), 2.11 (s, 3H), 1.36 (d, *J* = 6.0 Hz, 3H), 1.31 (d, *J* = 6.0 Hz, 3H), 1.15 (s, 9H). LC-MS (ESI) m/z: $[M+H]^+$1247.5

N-(3-((2-((4-(4-(2-(2-(2-(2-azidoethoxy)ethoxy)ethoxy)ethyl)piperazin-1-yl)phenyl)amino)-5-methylpyrimidin-4-yl)amino)phenyl)-2-methylpropane-2-sulfonamide (18). Compound 18 was synthesized from compound 9 and linker (2-(2-

(2-(2-azidoethoxy)ethoxy)ethoxy)ethyl 4-methylbenzenesulfonate) according to the representative procedure described for the synthesis of 15. ^{1}H NMR (400 MHz, $CDCl_3$) δ 8.23 (s, 1H), 7.86 (s, 1H), 7.82 (d, *J* = 8.0 Hz, 1H), 7.57 (d, *J* = 7.8 Hz, 1H), 7.37 (m, 3H), 7.07 (s, 1H), 6.90 (d, *J* = 8.7 Hz, 2H), 6.66 (s, 1H), 3.73 (t, *J* = 5.5 Hz, 2H), 3.70 – 3.61 (m, 10H), 3.39 (t, *J* = 4.8 Hz, 2H), 3.25 (m, 4H), 2.82 (m, 4H), 2.78 (m, 2H), 2.12 (s, 3H), 1.18 (s, 9H). LC-MS (ESI) m/z: $[M+H]^+$697.2

2-(4-(4,5-bis(4-chlorophenyl)-2-(2-isopropoxy-4-methoxyphenyl)-4,5-dihydro-1H-imidazole-1-carbonyl)-2-oxopiperazin-1-yl)-N-(2-(2-(2-(2-(4-(4-((4-((3-(N-(tert-butyl)sulfamoyl)phenyl)amino)-5-methylpyrimidin-2-yl)amino)phenyl)piperazin-1-yl)ethoxy)ethoxy)ethoxy)ethyl)acetamide (SJW-8139). Compound SJW-8139 was synthesized from compound 18 and 22 according to the representative two-step procedure described for the synthesis of SJW-8134. ^{1}H NMR (400 MHz, $CDCl_3$) δ 8.23 (s, 1H), 7.87 (s, 1H), 7.74 (d, *J* = 8.1 Hz, 1H), 7.56 (t, *J* = 8.0 Hz, 2H), 7.36 (t, *J* = 7.9 Hz, 2H), 7.28 (d, *J* = 8.7 Hz, 2H), 7.11 (s, 1H), 7.05 (d, *J* = 8.4 Hz, 2H), 7.00 (d, *J* = 8.6 Hz, 2H), 6.95 – 6.81 (m, 6H), 6.60 (s, 1H), 6.51 (dd, *J* = 8.5, 2.1 Hz, 1H), 6.46 (d, *J* = 1.9 Hz, 1H), 5.56 (d, *J* = 9.7 Hz, 1H), 5.46 (d, *J* = 9.7 Hz, 1H), 4.99 (s, 1H), 4.58 (m, 1H), 3.84 (s, 3H), 3.81 (s, 2H), 3.74 (m, 1H), 3.69 – 3.56 (m, 11H), 3.52 – 3.47 (m, 2H), 3.44 – 3.32 (m, 3H), 3.20 (s, 5H), 3.00 (s, 1H), 2.90 (d, *J* = 7.7 Hz, 1H), 2.75 (m, 4H), 2.71 (m, 2H), 2.10 (s, 3H), 1.36 (d, *J* = 6.0 Hz, 3H), 1.31 (d, *J* = 6.0 Hz, 3H), 1.15 (s, 9H). LC-MS (ESI) m/z: $[M+H]^+$1291.2

N-(3-((2-((4-(4-(14-azido-3,6,9,12-tetraoxatetradecyl)piperazin-1-yl)phenyl)amino)-5-methylpyrimidin-4-yl)amino)phenyl)-2-methylpropane-2-sulfonamide (19). Compound 19 was synthesized from compound 9 and linker (14-azido-3,6,9,12-tetraoxatetradecyl 4-methylbenzenesulfonate) according to the representative procedure described for the synthesis of 15. ^{1}H NMR (400 MHz, $CDCl_3$) δ 8.25 (s, 1H), 7.77 (d, *J* = 8.7 Hz, 1H), 7.71 (s, 1H), 7.64 (d, *J* = 7.7 Hz, 1H), 7.40 – 7.32 (m, 3H), 6.81 (d, *J* = 8.8 Hz, 2H), 5.17 (s, 1H), 3.92 (m, 2H), 3.68 – 3.58 (m, 14H), 3.38 (m, 6H), 3.21 (m, 4H), 3.13 – 3.03 (m, 2H), 2.16 (s, 3H), 1.19 (s, 9H). LC-MS (ESI) m/z: $[M+H]^+$741.2

2-(4-(4,5-bis(4-chlorophenyl)-2-(2-isopropoxy-4-methoxyphenyl)-4,5-dihydro-1H-imidazole-1-carbonyl)-2-oxopiperazin-1-yl)-N-(14-(4-(4-((4-((3-((1,1-dimethylethyl)sulfonamido)phenyl)amino)-5-methylpyrimidin-2-yl)amino)phenyl)piperazin-1-yl)-3,6,9,12-tetraoxatetradecyl)acetamide (SJW-8140). Compound SJW-8140 was synthesized from compound 19 and 22 according to the representative two-step procedure described for the synthesis of SJW-8134. ^{1}H NMR (400 MHz, $CDCl_3$) δ 8.22 (s, 1H), 7.88 (s, 1H), 7.76 (d, *J* = 9.0 Hz, 1H), 7.57 (t, *J* = 8.6 Hz, 2H), 7.41 – 7.33 (m, 3H), 7.06 (d, *J* = 8.4 Hz, 2H), 7.02 (d, *J* = 8.5 Hz, 2H), 6.97 – 6.83 (m, 7H), 6.58 (s, 1H), 6.54 – 6.49 (m, 1H), 6.46 (s, 1H), 5.54 (d, *J* = 9.7 Hz, 1H), 5.47 (d, *J* = 9.6 Hz, 1H), 4.92 (s, 1H), 4.60 (dt, *J* = 12.0, 6.0 Hz, 1H), 3.95 (d, *J* = 15.8 Hz, 1H), 3.86 (d, *J* = 15.9 Hz, 4H), 3.78 (d, *J* = 15.7 Hz, 1H), 3.72 –

3.55 (m, 15H), 3.53 – 3.46 (m, 3H), 3.40 – 3.34 (m, 2H), 3.22 (s, 5H), 3.07 (m, 2H), 2.85 – 2.71 (m, 6H), 2.11 (s, 3H), 1.36 (d, J = 6.0 Hz, 3H), 1.32 (d, J = 6.0 Hz, 3H), 1.15 (s, 9H). LC-MS (ESI) m/z: $[M+H]^+$1335.2

***tert*-Butyl (5-(4-((4-((3-(N-(tert-butyl)sulfamoyl)phenyl)amino)-5-methyl pyrimidin-2-yl)amino)phenoxy)pentyl)carbamate (20).** Compound 10 (128 mg, 0.3 mmol) was dissolved in DMF, then the linker (6-((tert-butoxycarbonyl)amino)hexyl 4-methylbenzenesulfonate, 280 mg, 0.75 mmol), and Cs_2CO_3 (292 mg, 0.9 mmol) were added. The mixture was stirred at RT for overnight. The reaction was diluted with EA and washed with water for three times. The organic phase was concentrated, and the residue was purified by flash chromatography DCM: MeOH (2% ammonia) = 98:2, giving product (140 mg, 74%). ^{1}H NMR (400 MHz, $CDCl_3$) δ 8.11 (s, 1H), 7.86 (s, 2H), 7.58 (d, J = 7.7 Hz, 1H), 7.38 (m, 3H), 7.14 (s, 1H), 6.86 (d, J = 8.9 Hz, 2H), 6.57 (s, 1H), 4.71 (s, 1H), 4.56 (s, 1H), 3.96 (t, J = 6.5 Hz, 2H), 3.13 (m, 2H), 2.12 (s, 3H), 1.82 – 1.74 (m, 2H), 1.54 – 1.47 (m, 4H), 1.44 (s, 9H), 1.42 – 1.36 (m, 2H), 1.20 (s, 9H). LC-MS (ESI) m/z: $[M+H]^+$627.0

2-(4-(4,5-bis(4-chlorophenyl)-2-(2-isopropoxy-4-methoxyphenyl)-4,5-dihydro-1H-imidazole-1-carbonyl)-2-oxopiperazin-1-yl)-N-(6-(4-((4-((3-(N-(tert-butyl) sulfamoyl)phenyl)amino)-5-methylpyrimidin-2-yl)amino)phenoxy)hexyl) acetamide (SJW-8142). Compound SJW-8142 was synthesized from compound 20 and 22 according to the representative two-step procedure described for the synthesis

of SJW-8121. ^{1}H NMR (400 MHz, $CDCl_3$) δ 8.18 (s, 1H), 7.87 (s, 1H), 7.78 (dd, *J* = 8.1, 1.4 Hz, 1H), 7.58 (d, *J* = 8.5 Hz, 1H), 7.54 (d, *J* = 8.0 Hz, 1H), 7.35 (m, 3H), 7.12 (s, 1H), 7.06 (d, *J* = 8.5 Hz, 2H), 7.01 (d, *J* = 8.5 Hz, 2H), 6.92 (d, *J* = 8.5 Hz, 2H), 6.86 (dd, *J* = 8.6, 6.5 Hz, 4H), 6.64 (s, 1H), 6.52 (dd, *J* = 8.3, 1.9 Hz, 1H), 6.46 (d, *J* = 2.2 Hz, 1H), 6.29 (t, *J* = 5.8 Hz, 1H), 5.53 (d, *J* = 9.6 Hz, 1H), 5.45 (d, *J* = 9.6 Hz, 1H), 5.16 (s, 1H), 4.65 – 4.54 (m, 1H), 3.94 (m, 3H), 3.87 (d, *J* = 4.2 Hz, 1H), 3.83 (s, 3H), 3.72 (d, *J* = 15.2 Hz, 1H), 3.68 – 3.61 (m, 1H), 3.51 (m, 1H), 3.25 – 3.07 (m, 5H), 2.10 (s, 3H), 1.78 – 1.70 (m, 2H), 1.45 (m, 4H), 1.36 (m, 4H), 1.32 (m, 4H), 1.17 (s, 9H). LC-MS (ESI) m/z: $[M+H]^+$1147.2

3-((2-((4-(2-(2-(2-azidoethoxy)ethoxy)ethoxy)phenyl)amino)-5-methylpyrimidin-4-yl)amino)-N-(tert-butyl)benzenesulfonamide (21). Compound 21 was synthesized from compound 10 and linker (2-(2-(2-azidoethoxy)ethoxy)ethyl 4-methylbenzenesulfonate) according to the representative procedure described for the synthesis of 20. ^{1}H NMR (400 MHz, $CDCl_3$) δ 8.12 (s, 1H), 7.90 – 7.81 (m, 2H), 7.54 (dd, *J* = 15.0, 7.0 Hz, 1H), 7.42 – 7.34 (m, 3H), 7.04 (s, 1H), 6.87 (t, *J* = 8.1 Hz, 2H), 6.53 (s, 1H), 4.86 (s, 1H), 4.13 (m, 2H), 3.89 – 3.85 (m, 2H), 3.77 – 3.73 (m, 2H), 3.72 – 3.67 (m, 4H), 3.42 – 3.35 (m, 2H), 2.11 (s, 3H), 1.21 (s, 9H). LC-MS (ESI) m/z: $[M+H]^+$585.1

2-(4-(4,5-bis(4-chlorophenyl)-2-(2-isopropoxy-4-methoxyphenyl)-4,5-dihydro-1H-imidazole-1-carbonyl)-2-oxopiperazin-1-yl)-N-(2-(2-(2-(4-((4-((3-(N-(tert-

butyl) sulfamoyl)phenyl)amino)-5-methylpyrimidin-2-yl)amino)phenoxy)ethoxy)ethoxy) ethyl)acetamide (SJW-8136). Compound SJW-8136 was synthesized from compound 21 and 22 according to the representative two-step procedure described for the synthesis of SJW-8134. ^{1}H NMR (400 MHz, $CDCl_3$) δ 8.18 (s, 1H), 7.85 (s, 1H), 7.75 (d, *J* = 8.0 Hz, 1H), 7.56 (t, *J* = 8.3 Hz, 2H), 7.36 (m, 3H), 7.27 (s, 1H), 7.05 (d, *J* = 8.4 Hz, 2H), 7.00 (d, *J* = 8.4 Hz, 2H), 6.91 (d, *J* = 8.4 Hz, 2H), 6.87 (m, 4H), 6.70 (s, 2H), 6.55 – 6.48 (m, 1H), 6.45 (d, *J* = 1.9 Hz, 1H), 5.53 (d, *J* = 9.6 Hz, 1H), 5.45 (d, *J* = 9.6 Hz, 1H), 5.20 (s, 1H), 4.59 (dt, *J* = 12.1, 6.1 Hz, 1H), 4.16 – 4.09 (m, 2H), 3.90 (d, *J* = 15.6 Hz, 1H), 3.83 (m, 6H), 3.73 (d, *J* = 15.2 Hz, 1H), 3.70 – 3.59 (m, 5H), 3.53 (t, *J* = 5.0 Hz, 2H), 3.46 (m, 1H), 3.43 – 3.37 (m, 2H), 3.22 (m, 1H), 3.12 – 3.01 (m, 2H), 2.10 (s, 3H), 1.36 (d, *J* = 6.0 Hz, 3H), 1.31 (d, *J* = 6.0 Hz, 3H), 1.18 (s, 9H). LC-MS (ESI) m/z: $[M+H]^+$1179.2

Scheme 3[a].

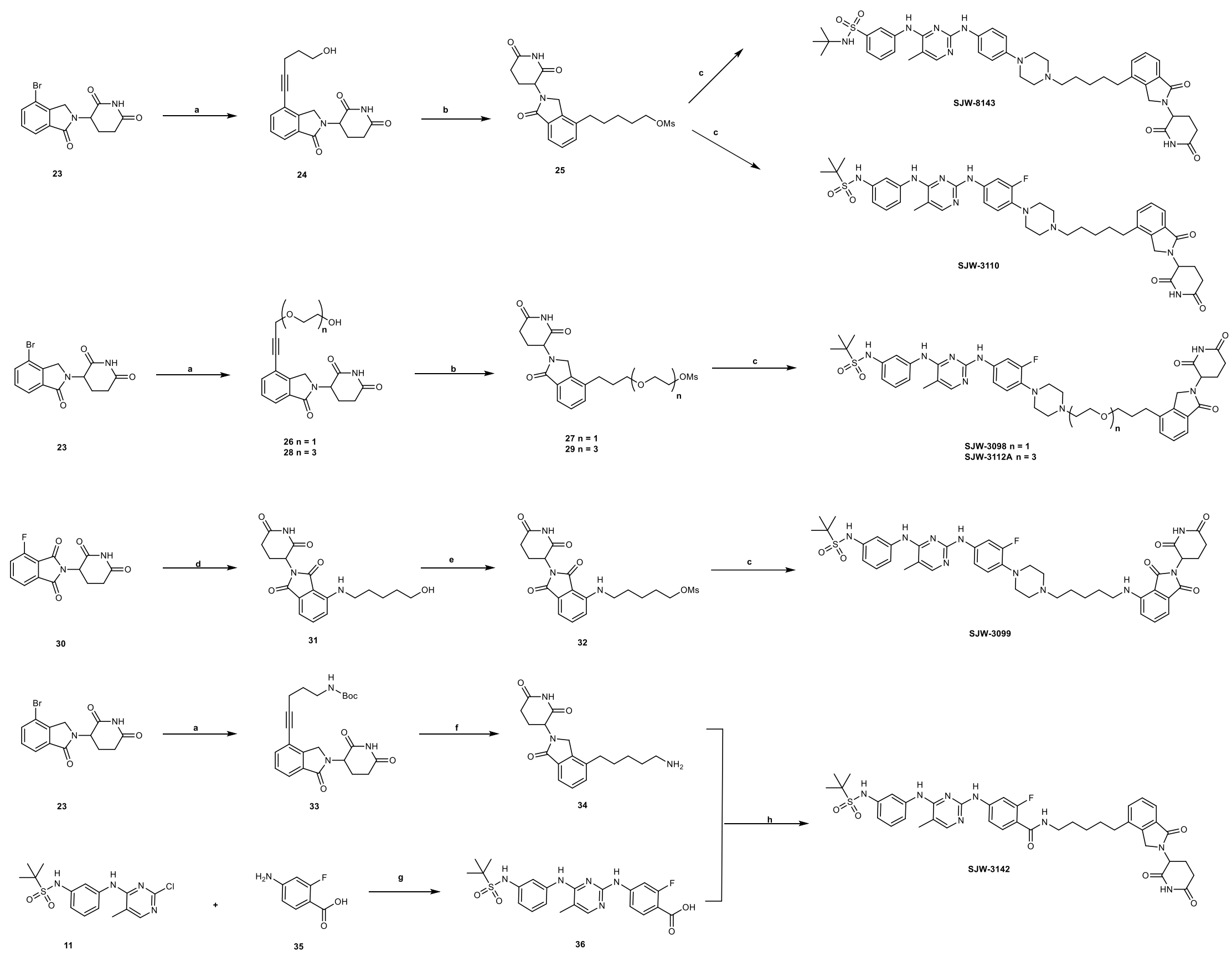

[a]Reagent and conditions: (a) linker, $Pd(PPh_3)_2Cl_2$, CuI, TEA, DMF, 70 ºC, 15h; (b) 1) Pd/C, H_2, MeOH; 2) MsCl, TEA, DCM; (c) 9 or 14, DIPEA, KI, DMF, 60 ºC; (d) 5-aminopentan-1-ol, DIPEA, DMA, 90ºC; (e) MsCl, TEA, DCM; (f) 1) Pd/C, H_2, MeOH; 2) TFA, DCM; (g) 4M HCl in dioxane; BuOH, 80 ºC; (h) HATU, TEA, DMF.

3-(4-(5-hydroxypent-1-yn-1-yl)-1-oxoisoindolin-2-yl)piperidine-2,6-dione (24).

Compound 23 (969 mg, 3 mmol), pent-4-yn-1-ol (505 mg, 6 mmol), CuI (114 mg, 0.6 mmol) and $Pd(PPh_3)_2Cl_2$ (210 mg, 0.3 mmol) were mixed and dissolved in TEA (6 mL) and DMF (20 mL). the mixture was flushed with N_2 and heated to 70 ºC under N_2 atmosphere overnight. The solvent was removed, and the residue was by flash chromatography (DCM: MeOH = 98:2), giving product (720 mg, 74%). ^{1}H NMR

(400 MHz, DMSO-d6) δ 11.00 (s, 1H), 7.70 (d, *J* = 7.3 Hz, 1H), 7.63 (d, *J* = 7.4 Hz, 1H), 7.51 (t, *J* = 7.6 Hz, 1H), 5.14 (dd, *J* = 13.1, 4.9 Hz, 1H), 4.57 (t, *J* = 5.0 Hz, 1H), 4.45 (d, *J* = 17.8 Hz, 1H), 4.30 (d, *J* = 17.8 Hz, 1H), 3.53 (m, 2H), 3.00 – 2.83 (m, 1H), 2.59 (m, 1H), 2.53 (m, 2H), 2.44 (m, 1H), 2.06 – 1.93 (m, 1H), 1.71 (m, 2H).

5-(2-(2,6-dioxopiperidin-3-yl)-1-oxoisoindolin-4-yl)pentyl methanesulfonate (25). Compound 24 (326 mg, 1 mmol) was dissolved in MeOH. 10% palladium on carbon (60 mg) was added. The suspension was kept under hydrogen atmosphere (1 atm) for 15 h and was then filtered through a pad of celite. The filtrate was concentrated in vacuo. The residue was dissolved in DCM, then TEA (416 µL, 3.0 mmol) and MsCl (164 µL, 1.5 mmol) were added. The mixture was stirred at RT overnight. The solvent was removed, and the residue was partitioned between water and CH_2Cl_2. The organic layer was separated and concentrated in vacuo. The residue was purified by flash chromatography DCM: MeOH = 98:2, giving product (253 mg, 62%). ^{1}H NMR (400 MHz, DMSO-d_6) δ 10.99 (s, 1H), 7.57 (m, 1H), 7.51 – 7.41 (m, 2H), 5.13 (dd, *J* = 13.2, 5.1 Hz, 1H), 4.47 (d, *J* = 17.2 Hz, 1H), 4.31 (d, *J* = 17.2 Hz, 1H), 4.19 (t, *J* = 6.4 Hz, 2H), 3.15 (s, 3H), 2.98 – 2.86 (m, 1H), 2.64 (m, 3H), 2.48 – 2.36 (m, 1H), 2.06 – 1.95 (m, 1H), 1.76 – 1.58 (m, 4H), 1.41 (m, 2H).

N-(tert-butyl)-3-((2-((4-(4-(5-(2-(2,6-dioxopiperidin-3-yl)-1-oxoisoindolin-4-yl)pentyl)piperazin-1-yl)phenyl)amino)-5-methylpyrimidin-4-yl)amino)

benzenesulfonamide (SJW-8143). Compound 9 (50 mg, 0.1 mmol), compound 25 (60 mg, 0.15 mmol), KI (25 mg, 0.15 mmol) and DIPEA (50 µL, 0.3 mmol) were mixed and dissolved in DMF. The reaction was heated to 70°C overnight. The solvent was removed, and the residue was purified by flash chromatography (DCM: MeOH = 97:3), giving product (24 mg, 31%). ^{1}H NMR (400 MHz, $CDCl_3$) δ 8.19 (s, 1H), 7.90 (s, 1H), 7.78 (d, *J* = 8.1 Hz, 1H), 7.73 (d, *J* = 7.3 Hz, 1H), 7.54 (d, *J* = 8.4 Hz, 1H), 7.44 (t, *J* = 7.4 Hz, 1H), 7.39 (d, *J* = 3.5 Hz, 2H), 7.37 (t, *J* = 5.7 Hz, 2H), 7.32 (m, 2H), 6.88 (d, *J* = 8.9 Hz, 2H), 6.57 (s, 1H), 5.27 (dd, *J* = 13.0, 4.8 Hz, 1H), 4.90 (s, 1H), 4.48 (d, *J* = 16.0 Hz, 1H), 4.32 (d, *J* = 16.0 Hz, 1H), 3.20 (m, 4H), 2.95 – 2.76 (m, 1H), 2.65 (m, 7H), 2.42 (m, 3H), 2.21 (m, 1H), 2.10 (s, 3H), 1.68 (m, 3H), 1.61 (m, 2H), 1.41 (m, 2H), 1.16 (s, 9H). LC-MS (ESI) m/z: $[M+H]^+$ 808.4

N-(3-((2-((4-(4-(5-(2-(2,6-dioxopiperidin-3-yl)-1-oxoisoindolin-4-yl)pentyl) piperazin-1-yl)-3-fluorophenyl)amino)-5-methylpyrimidin-4-yl)amino)phenyl)-2-methylpropane-2-sulfonamide (SJW-3110). Compound SJW-3110 was synthesized from compound 14 and 25 according to the representative procedure described for the synthesis of SJW-8143. ^{1}H NMR (400 MHz, DMSO-d_6) δ 11.00 (s, 1H), 9.57 (s, 1H), 8.88 (s, 1H), 8.38 (s, 1H), 7.88 (s, 1H), 7.62 (d, *J* = 15.5 Hz, 1H), 7.58 – 7.54 (m, 1H), 7.46 (m, 4H), 7.23 (m, 2H), 7.01 (d, *J* = 7.3 Hz, 1H), 6.85 (t, *J* = 9.4 Hz, 1H), 5.13 (dd, *J* = 13.0, 4.9 Hz, 1H), 4.47 (d, *J* = 17.2 Hz, 1H), 4.31 (d, *J* = 17.2 Hz, 1H), 3.00 – 2.81 (m, 5H), 2.64 (m, 3H), 2.49 – 2.44 (m, 4H), 2.44 – 2.37 (m, 1H), 2.28 (d, *J* =

18.9 Hz, 2H), 2.09 (s, 3H), 2.05 – 1.96 (m, 1H), 1.69 – 1.58 (m, 2H), 1.54 – 1.43 (m, 2H), 1.41 – 1.32 (m, 2H), 1.25 (s, 9H).

3-(4-(3-(2-hydroxyethoxy)prop-1-yn-1-yl)-1-oxoisoindolin-2-yl)piperidine-2,6-dione (26). Compound 26 was synthesized from compound 23 and 2-(prop-2-yn-1-yloxy)ethan-1-ol according to the representative procedure described for the synthesis of 24. ^{1}H NMR (400 MHz, DMSO-d_6) δ 11.01 (s, 1H), 7.79 – 7.75 (m, 1H), 7.71 (dt, *J* = 7.1, 3.6 Hz, 1H), 7.56 (t, *J* = 6.2 Hz, 1H), 5.14 (dd, *J* = 13.3, 5.1 Hz, 1H), 4.71 – 4.68 (m, 1H), 4.50 (d, *J* = 17.8 Hz, 1H), 4.46 (m, 2H), 4.38 – 4.30 (d, *J* = 17.8 Hz, 1H), 3.59 – 3.51 (m, 4H), 2.96 – 2.82 (m, 1H), 2.59 (m, 1H), 2.48 – 2.39 (m, 1H), 2.08 – 1.96 (m, 1H).

2-(3-(2-(2,6-dioxopiperidin-3-yl)-1-oxoisoindolin-4-yl)propoxy)ethyl methane sulfonate (27). Compound 27 was synthesized from compound 26 according to the representative two-step procedure described for the synthesis of 25. ^{1}H NMR (400 MHz, DMSO-d_6) δ 11.00 (s, 1H), 7.57 (dd, *J* = 6.1, 2.5 Hz, 1H), 7.48 – 7.45 (m, 2H), 5.13 (dd, *J* = 13.3, 5.1 Hz, 1H), 4.46 (d, *J* = 17.2 Hz, 1H), 4.34 – 4.27 (m, 3H), 3.65 – 3.61 (m, 2H), 3.46 – 3.41 (m, 2H), 3.17 (s, 3H), 2.97 – 2.86 (m, 1H), 2.70 (t, *J* = 7.7 Hz, 2H), 2.64 – 2.56 (m, 1H), 2.42 (m, 1H), 2.05 – 1.97 (m, 1H), 1.91 – 1.80 (m, 2H).

N-(3-((2-((4-(4-(2-(3-(2-(2,6-dioxopiperidin-3-yl)-1-oxoisoindolin-4-yl)propoxy) ethyl)piperazin-1-yl)-3-fluorophenyl)amino)-5-methylpyrimidin-4-yl)amino)

phenyl)-2-methylpropane-2-sulfonamide (SJW-3098). Compound SJW-3098 was synthesized from compound 14 and 27 according to the representative procedure described for the synthesis of SJW-8143. ^{1}H NMR (400 MHz, DMSO-d_6) δ 11.00 (s, 1H), 9.58 (s, 1H), 8.89 (s, 1H), 8.39 (s, 1H), 7.88 (s, 1H), 7.63 (d, J = 15.7 Hz, 1H), 7.58 (dd, J = 6.1, 2.4 Hz, 1H), 7.52 – 7.43 (m, 4H), 7.26 (m, 1H), 7.21 (t, J = 8.0 Hz, 1H), 7.01 (d, J = 8.7 Hz, 1H), 6.85 (t, J = 9.5 Hz, 1H), 5.14 (dd, J = 13.3, 5.1 Hz, 1H), 4.47 (d, J = 17.1 Hz, 1H), 4.31 (d, J = 17.1 Hz, 1H), 3.62 (m, 1H), 3.50 (m, 2H), 3.40 (m, 2H), 3.13 (m, 1H), 2.93 (m, 5H), 2.70 (t, J = 7.6 Hz, 2H), 2.59 (m, 5H), 2.47 – 2.36 (m, 1H), 2.09 (s, 3H), 2.05 – 1.96 (m, 1H), 1.86 (m, 2H), 1.27 (s, 9H).

3-(4-(3-(2-(2-(2-hydroxyethoxy)ethoxy)ethoxy)prop-1-yn-1-yl)-1-oxoisoindolin-2-yl)piperidine-2,6-dione (28). Compound 28 was synthesized from compound 23 and 2-(2-(2-(prop-2-yn-1-yloxy)ethoxy)ethoxy)ethan-1-ol according to the representative procedure described for the synthesis of 24. ^{1}H NMR (400 MHz, DMSO-d_6) δ 11.01 (s, 1H), 7.75 (m, 1H), 7.67 – 7.61 (m, 1H), 7.57 (m, 1H), 5.14 (m, 1H), 4.59 (m, 1H), 4.50 (d, J = 17.8 Hz, 1H), 4.46 (m, 2H), 4.34 (d, J = 17.8 Hz, 1H), 3.62 (m, 2H), 3.50 (m, 6H), 3.40 (m, 2H), 2.90 (m 1H), 2.59 (m, 1H), 2.44 (m, 1H), 2.02 (m, 1H).

2-(2-(2-((3-(2-(2,6-dioxopiperidin-3-yl)-1-oxoisoindolin-4-yl)prop-2-yn-1-yl)oxy)

ethoxy)ethoxy)ethyl methanesulfonate (29). Compound 29 was synthesized from compound 28 according to the representative procedure described for the synthesis of 25.

N-(3-((2-((4-(4-(2-(2-(2-(3-(2-(2,6-dioxopiperidin-3-yl)-1-oxoisoindolin-4-yl)propoxy)ethoxy)ethoxy)ethyl)piperazin-1-yl)-3-fluorophenyl)amino)-5-methylpyrimidin-4-yl)amino)phenyl)-2-methylpropane-2-sulfonamide (SJW-3112A). ^{1}H NMR (400 MHz, CDCl3) δ 7.92 (s, 1H), 7.88 (s, 1H), 7.72 (d, *J* = 6.6 Hz, 1H), 7.67 (s, 1H), 7.47 (d, *J* = 15.3 Hz, 1H), 7.39 (m, 2H), 7.21 (m, 2H), 7.07 (m, 2H), 6.75 (t, *J* = 9.1 Hz, 1H), 6.46 (s, 1H), 5.28 (dd, *J* = 13.2, 5.0 Hz, 1H), 4.45 (d, *J* = 16.4 Hz, 1H), 4.31 (d, *J* = 16.4 Hz, 1H), 3.61 (m, 10H), 3.43 (s, 2H), 3.05 (m, 4H), 2.89 – 2.65 (m, 10H), 2.39 – 2.25 (m, 1H), 2.18 (m, 1H), 2.09 (s, 3H), 1.94 – 1.87 (m, 2H), 1.38 (s, 9H).

2-(2,6-dioxopiperidin-3-yl)-4-((5-hydroxypentyl)amino)isoindoline-1,3-dione (31). Compound 30 (552 mg, 2.0 mmol) was dissolved in DMA, DIPEA (650 μL, 4.0 mmol) and 5-aminopentan-1-ol (309 mg, 3.0 mmol) were added, the mixture was heated to 90 ºC for 15h. Then the mixture was absorbed on diatomite and purified by reversed-phase flash chromatography (H_2O: MeOH = 90:10 to 50:50), giving compounds 31 (460 mg, 64%). ^{1}H NMR (400 MHz, $CDCl_3$) δ 8.16 (s, 1H), 7.49 (dd, *J* = 8.5, 7.1 Hz, 1H), 7.09 (d, *J* = 7.1 Hz, 1H), 6.88 (d, *J* = 8.5 Hz, 1H), 4.91 (dd, *J* =

12.1, 5.4 Hz, 1H), 3.66 (q, J = 6.3 Hz, 2H), 3.28 (t, J = 7.0 Hz, 2H), 2.93 – 2.67 (m, 3H), 2.12 (m, 1H), 1.75 – 1.66 (m, 2H), 1.64 – 1.59 (m, 2H), 1.54 – 1.46 (m, 2H).

5-((2-(2,6-dioxopiperidin-3-yl)-1,3-dioxoisoindolin-4-yl)amino)pentyl methane sulfonate (32). Compound 31 (120 mg, 0.33 mmol) was dissolved in DCM, then TEA (141 μL, 1.32 mmol) and MsCl (72 μL, 0.66 mmol) were added. The mixture was stirred at RT for overnight. The solvent was removed, and the residue was partitioned between water and CH_2Cl_2. The organic layer was separated and concentrated in vacuo. The residue was used for next step without purification.

N-(3-((2-((4-(4-(5-((2-(2,6-dioxopiperidin-3-yl)-1,3-dioxoisoindolin-4-yl)amino) pentyl)piperazin-1-yl)-3-fluorophenyl)amino)-5-methylpyrimidin-4-yl)amino) phenyl)-2-methylpropane-2-sulfonamide (SJW-3099). Compound SJW-3099 was synthesized from compound 14 and 32 according to the representative procedure described for the synthesis of SJW-8143. ^{1}H NMR (400 MHz, DMSO-d_6) δ 11.10 (s, 1H), 9.59 (s, 1H), 8.96 (s, 1H), 8.41 (s, 1H), 7.89 (s, 1H), 7.67 (s, 1H), 7.62 – 7.57 (m, 1H), 7.48 (d, J = 8.4 Hz, 2H), 7.27 (d, J = 9.1 Hz, 1H), 7.22 (t, J = 8.1 Hz, 1H), 7.12 (d, J = 8.6 Hz, 1H), 7.02 (t, J = 7.0 Hz, 2H), 6.57 (s, 1H), 5.05 (dd, J = 12.9, 5.3 Hz, 1H), 3.18 – 3.11 (m, 4H), 3.01 – 2.82 (m, 5H), 2.62 – 2.51 (m, 5H), 2.47 (m, 1H), 2.10 (s, 3H), 2.06 – 1.97 (m, 1H), 1.62 (m, 2H), 1.39 (m, 2H), 1.28 (m, 11H).

***tert*-butyl (5-(2-(2,6-dioxopiperidin-3-yl)-1-oxoisoindolin-4-yl) pent-4-yn-1-yl)**

carbamate (33). Compound 33 was synthesized from compound 23 and *tert*-butyl pent-4-yn-1-ylcarbamate according to the representative procedure described for the synthesis of 24.

3-(4-(5-aminopentyl)-1-oxoisoindolin-2-yl) piperidine-2,6-dione (34). Compound 24 (500 mg, 1.18 mmol) was dissolved in MeOH. 10% palladium on carbon (100 mg) was added. The suspension was kept under hydrogen atmosphere (1 atm) for 15 h and was then filtered through a pad of celite. The filtrate was concentrated in vacuo. The residue was dissolved in DCM, then TFA (1 mL) were added. The mixture was stirred at RT for overnight. The reaction was concentrated, giving product. ^{1}H NMR (400 MHz, DMSO-d_6) δ 11.01 (s, 1H), 7.57 (dd, *J* = 8.3, 4.0 Hz, 1H), 7.46 (d, *J* = 4.3 Hz, 2H), 5.14 (dd, *J* = 13.3, 5.1 Hz, 1H), 4.46 (d, *J* = 17.1 Hz, 1H), 4.30 (d, *J* = 17.1 Hz, 1H), 2.99 – 2.86 (m, 1H), 2.83 – 2.73 (m, 2H), 2.63 (m, 3H), 2.45 – 2.31 (m, 1H), 2.08 – 1.94 (m, 1H), 1.59 (m, 4H), 1.42 – 1.30 (m, 2H). LC-MS (ESI) m/z: $[M+H]^+$

4-((4-((3-((1,1-dimethylethyl) sulfonamido) phenyl)amino)-5-methylpyrimidin-2-yl)amino)-2-fluorobenzoic acid (36). Compound 11 (177 mg, 0.5 mmol) and compound 35 (93 mg, 0.6 mmol) were mixed and suspended in BuOH. HCl solution (4M in dioxane, 0.1 mL) was added. The mixture was heated to 80°C in sealed tube for 15 h. the reaction was cooled to RT, then the solid was filtered and washed with BuOH, giving product (140 mg, 59%). ^{1}H NMR (400 MHz, DMSO-d_6) δ 10.97 (s, 1H), 9.95 (s, 1H), 9.84 (s, 1H), 8.02 (d, *J* = 0.9 Hz, 1H), 7.70 (t, *J* = 8.6 Hz, 1H), 7.50

(dd, *J* = 13.6, 2.0 Hz, 1H), 7.40 – 7.33 (m, 2H), 7.27 – 7.17 (m, 3H), 2.18 (s, 3H), 1.26 – 1.22 (s, 9H).

4-((4-((3-((1,1-dimethylethyl)sulfonamido)phenyl)amino)-5-methylpyrimidin-2-yl)amino)-N-(5-(2-(2,6-dioxopiperidin-3-yl)-1-oxoisoindolin-4-yl)pentyl)-2-fluoro benzamide (SJW-3142). Compound 35 (47 mg, 0.1 mmol), compound 34 (40 mg, 0.12 mmol) and HATU (57 mg, 0.15 mmol) were dissolved in DMF, then TEA (68 μL, 0.5 mmol) was added. The mixture was stirred at RT for 4h. the reaction solution was concentrated and purified by flash chromatography (DCM: MeOH = 93:7), giving product (30 mg, 23%). ^{1}H NMR (400 MHz, DMSO-d_6) δ 11.09 (s, 1H), 9.71 (s, 1H), 9.51 (s, 1H), 8.62 (s, 1H), 8.06 (d, *J* = 0.7 Hz, 1H), 7.97 (m, 1H), 7.87 (dd, *J* = 14.7, 1.8 Hz, 1H), 7.66 (dd, *J* = 6.3, 2.2 Hz, 1H), 7.60 – 7.51 (m, 4H), 7.47 (dd, *J* = 8.7, 1.9 Hz, 1H), 7.36 (t, *J* = 8.1 Hz, 1H), 7.14 (dd, *J* = 8.1, 1.2 Hz, 1H), 5.23 (dd, *J* = 13.3, 5.1 Hz, 1H), 4.57 (d, *J* = 17.2 Hz, 1H), 4.41 (d, *J* = 17.2 Hz, 1H), 3.32 (m, 2H), 3.07 – 2.96 (m, 1H), 2.79 – 2.72 (m, 2H), 2.67 (m, 1H), 2.58 – 2.47 (m, 1H), 2.22 (s, 3H), 2.15 – 2.06 (m, 1H), 1.72 (m, 2H), 1.64 (m, 2H), 1.47 (m, 2H), 1.37 (s, 9H).

Supplementary References

1. Rodrigues P*, et al.* NF-kappaB-Dependent Lymphoid Enhancer Co-option Promotes Renal Carcinoma Metastasis. *Cancer Discov* **8**, 850-865 (2018).

2. Chen Y*, et al.* ZMYND8 acetylation mediates HIF-dependent breast cancer progression and metastasis. *J Clin Invest* **128**, 1937-1955 (2018).

3. Zhang J*, et al.* VHL substrate transcription factor ZHX2 as an oncogenic driver in clear cell renal cell carcinoma. *Science* **361**, 290-295 (2018).

4. Nakayama Y, Enomoto D, Yamamoto K, Takara K. Molecular Characteristics of Everolimus-resistant Renal Cell Carcinoma Cells Generated by Continuous Exposure to Everolimus. *Anticancer Res* **43**, 4349-4357 (2023).

5. An J*, et al.* Truncated ERG Oncoproteins from TMPRSS2-ERG Fusions Are Resistant to SPOP-Mediated Proteasome Degradation. *Mol Cell* **59**, 904-916 (2015).

6. Zhang P*, et al.* Intrinsic BET inhibitor resistance in SPOP-mutated prostate cancer is mediated by BET protein stabilization and AKT-mTORC1 activation. *Nat Med* **23**, 1055-1062 (2017).

7. Hu C*, et al.* Design, synthesis, and biological evaluation of imidazoline derivatives as p53-MDM2 binding inhibitors. *Bioorg Med Chem* **19**, 5454-5461 (2011).

8. Schneekloth AR, Pucheault M, Tae HS, Crews CM. Targeted intracellular protein degradation induced by a small molecule: En route to chemical proteomics. *Bioorg Med Chem Lett* **18**, 5904-5908 (2008).
