# Supplementary material for: SPOP-mediated ZMYND8 ubiquitination and phase separation exclusion drives mTOR inhibitor resistance in kidney cancer
Source: Nat Commun. 2026 Aug 25;17:10147. doi: 10.1038/s41467-026-77043-9 (PMC13601609; doi:10.1038/s41467-026-77043-9)
Supplement: Supplementary file 2 [file 41467_2026_77043_MOESM2_ESM.pdf]

nature portfolio

Corresponding author(s): Haojie Huang, Yige Bao, Chengheng Liao, Qiang Wei

Last updated by author(s): Dec 31, 2025

Reporting Summary

Nature Portfolio wishes to improve the reproducibility of the work that we publish. This form provides structure for consistency and transparency in reporting. For further information on Nature Portfolio policies, see our Editorial Policies and the Editorial Policy Checklist.

Statistics

For all statistical analyses, confirm that the following items are present in the figure legend, table legend, main text, or Methods section.

n/a	Confirmed	
☐	☒	The exact sample size (*n*) for each experimental group/condition, given as a discrete number and unit of measurement
☐	☒	A statement on whether measurements were taken from distinct samples or whether the same sample was measured repeatedly
☐	☒	The statistical test(s) used AND whether they are one- or two-sided<br>*Only common tests should be described solely by name; describe more complex techniques in the Methods section.*
☒	☐	A description of all covariates tested
☒	☐	A description of any assumptions or corrections, such as tests of normality and adjustment for multiple comparisons
☐	☒	A full description of the statistical parameters including central tendency (e.g. means) or other basic estimates (e.g. regression coefficient) AND variation (e.g. standard deviation) or associated estimates of uncertainty (e.g. confidence intervals)
☐	☒	For null hypothesis testing, the test statistic (e.g. *F*, *t*, *r*) with confidence intervals, effect sizes, degrees of freedom and *P* value noted<br>*Give P values as exact values whenever suitable.*
☒	☐	For Bayesian analysis, information on the choice of priors and Markov chain Monte Carlo settings
☒	☐	For hierarchical and complex designs, identification of the appropriate level for tests and full reporting of outcomes
☒	☐	Estimates of effect sizes (e.g. Cohen's *d*, Pearson's *r*), indicating how they were calculated

Our web collection on statistics for biologists contains articles on many of the points above.

Software and code

Policy information about availability of computer code

Data collection	CFX96 Real-Time System (Bio-Rad, USA) were utilized to conduct the quantitave PCR. Leica Application Suit V4 was used to take IHC pictures. Olympus SpinSR Rotary Confocal Microscope was used to take fluorescent pictures.Microsoft Excel version 2019 was used to process the data.
Data analysis	For graphing, we used Graphpad prism 10. For statistical analysis, we used IBM SPSS Statistics 20 and Microsoft Excel version 2019. For phase separation foci count, we used Image-Pro software version 10.

For manuscripts utilizing custom algorithms or software that are central to the research but not yet described in published literature, software must be made available to editors and reviewers. We strongly encourage code deposition in a community repository (e.g. GitHub). See the Nature Portfolio guidelines for submitting code & software for further information.

Data

Policy information about availability of data

All manuscripts must include a data availability statement. This statement should provide the following information, where applicable:

- Accession codes, unique identifiers, or web links for publicly available datasets
- A description of any restrictions on data availability
- For clinical datasets or third party data, please ensure that the statement adheres to our policy

Proteomics raw data are available in the PRIDE archive and can be accessed under ProteomeXchange accession number: PXD058853. All data are included in the main figure, Supplementary Information.

Research involving human participants, their data, or biological material

Policy information about studies with human participants or human data. See also policy information about sex, gender (identity/presentation), and sexual orientation and race, ethnicity and racism.

Reporting on sex and gender	Sex was not limited in this study. The kidney cancer tissue microarray (TMA) slides were generated from both male and female patients.
Reporting on race, ethnicity, or other socially relevant groupings	This study was not involved with the constructs of race and/or ethnicity
Population characteristics	Archive tumor tissue collected at the Mayo Clinic (Rochester, MN, U.S.A.). 50 cases (age from 45-80) were selected solely based on their histopathology diagnosis and tumor tissue availability.
Recruitment	Samples were collected retrospectively from the Mayo Clinic and annotated for major clinicopathologic variables through review of pathology reports and clinical records by trained personnel. Archive tumor tissue were selected solely based on their histopathology diagnosis and tumor tissue availability.
Ethics oversight	The studies were approved by the Ethics Committee of the Mayo Clinic (Rochester, MN, U.S.A.).

Note that full information on the approval of the study protocol must also be provided in the manuscript.

Field-specific reporting

Please select the one below that is the best fit for your research. If you are not sure, read the appropriate sections before making your selection.

[x] Life sciences [ ] Behavioural & social sciences [ ] Ecological, evolutionary & environmental sciences

For a reference copy of the document with all sections, see nature.com/documents/nr-reporting-summary-flat.pdf

Life sciences study design

All studies must disclose on these points even when the disclosure is negative.

Sample size	No sample size calculation was performed. Sample size was determined from similar experiments in the literature and for all experiments a minimum of 3 biological replicates were analyzed per sample.
Data exclusions	No data were excluded from the analyses.
Replication	Data are presented as the mean + SD. Replicates of experiments are specified in figure legends
Randomization	In mouse experiments, all samples and animals are randomly divided into different experimental groups as indicated. For otherstudies, experiments were performed in large celline populations, and randomization was therefore not appropriate, but the treated groupswere attributed randomly.
Blinding	For mouse study, investigators were blinded for group allocation during data colection and analysis. For other studies, experiments wereperformed blinded.

Reporting for specific materials, systems and methods

We require information from authors about some types of materials, experimental systems and methods used in many studies. Here, indicate whether each material, system or method listed is relevant to your study. If you are not sure if a list item applies to your research, read the appropriate section before selecting a response.

Materials & experimental systems

n/a	Involved in the study	
[ ]	[x]	Antibodies
[ ]	[x]	Eukaryotic cell lines
[x]	[ ]	Palaeontology and archaeology
[ ]	[x]	Animals and other organisms
[x]	[ ]	Clinical data
[x]	[ ]	Dual use research of concern
[x]	[ ]	Plants

Methods

n/a	Involved in the study	
[x]	[ ]	ChIP-seq
[x]	[ ]	Flow cytometry
[x]	[ ]	MRI-based neuroimaging

Antibodies

Antibodies used

SPOP(Proteintech,#16750-1-AP), ZMYND8(Bethyl Lab Inc.,#A302-089A), BRD2(Abcam,#ab139690), CHD3(Abcam,#ab109195), CAPRIN1(Proteintech,#15112-1-AP), SETD2(Proteintech,#55377-1-AP), BRD4(Abcam,#ab128874), GLI3(Abcam,#ab181130), DAXX (Abcam,#ab32140), INF2(Abcam,#ab245555), SRRM1(Abcam,#ab221061), Phospho-p70S6K (Thr389)(Cell Signaling Technology,#9205), p70S6K (Cell Signaling Technology,#9202), Phospho-4E-BP1 (Thr37/46)(Cell Signaling Technology,#2855), 4E-BP1 (Cell Signaling Technology,#9644), Phospho-mTOR (Ser2448)(Cell Signaling Technology,#5536), mTOR(Cell Signaling Technology,#2983), Phospho AKT (Thr308)(Cell Signaling Technology,#9275), AKT(Santa Cruz,#SC-5298), Phospho ERK1/2 (T202/Y204) (Cell Signaling Technology,#4370S), ERK2(Santa Cruz,#SC-1647), ERK1(Santa Cruz,#SC-93), Phospho MEK1/2 (Ser217/221)(Cell Signaling Technology,#9121), MEK2(Cell Signaling Technology,#9125), Phospho SAPK/JNK (Thr183/Ty185)(Cell Signaling Technology,#9251), SAPK/JNK (Cell Signaling Technology,#9252), Myc tag(Santa Cruz,#SC-40), FLAG tag(Proteintech,#66008-3-IG), Ubiquitin(Cell Signaling Technology,#3936), GFP tag(Proteintech,#50430-2-AP), HA tag(Proteintech,#66006-2-IG), ZHX2 (Genetex,#GTX112232), NEK7 (Bethyl Lab Inc.,#A302-684A), STAT3(Cell Signaling Technology,#9139), Phospho STAT3 (Tyr705)(Cell Signaling Technology,#9145), JAK2(Santa Cruz,#sc-390539), Ki67(Abcam,#ab15580), Cleaved Caspase-3(Cell Signaling Technology,#9579)

Validation

SPOP(Proteintech,#16750-1-AP), https://www.ptglab.com/products/SPOP-Antibody-16750-1-AP.htm;
ZMYND8(Bethyl Lab Inc.,#A302-089A), https://www.fortislife.com/products/primary-antibodies/rabbit-anti-prkcbp1-antibody/BETHYL-A302-089 ;
BRD2(Abcam,#ab139690), https://www.abcam.cn/products/primary-antibodies/brd2-antibody-epr7642-chip-grade-ab139690.html;
CHD3(Abcam,#ab109195), https://www.abcam.cn/products/primary-antibodies/chd3-antibody-epncir110a-ab109195.html;
CAPRIN1(Proteintech,#15112-1-AP), https://www.ptglab.com/products/CAPRIN1-Antibody-15112-1-AP.htm;
SETD2(Proteintech,#55377-1-AP), https://www.ptglab.com/products/SETD2-Antibody-55377-1-AP.htm;
BRD4(Abcam,#ab128874), https://www.abcam.cn/products/primary-antibodies/brd4-antibody-epr51502-ab128874.html;
GLI3(Abcam,#ab181130), https://www.abcam.cn/products/primary-antibodies/gli3-antibody-epr4594-ab181130.html;
DAXX(Abcam,#ab32140), https://www.abcam.cn/products/primary-antibodies/daxx-antibody-e94-ab32140.html;
INF2(Abcam,#ab245555),https://www.abcam.cn/products/primary-antibodies/inf2-antibody-ab245555.html;
SRRM1(Abcam,#ab221061), https://www.abcam.cn/products/primary-antibodies/srrm1-antibody-ab221061.html;
Phospho-p70S6K (Thr389)(Cell Signaling Technology,#9205), https://www.cellsignal.cn/products/primary-antibodies/phospho-p70-s6-kinase-thr389-antibody/9205;
p70S6K (Cell Signaling Technology,#9202), https://www.cellsignal.cn/products/primary-antibodies/p70-s6-kinase-antibody/9202;
Phospho-4E-BP1 (Thr37/46)(Cell Signaling Technology,#2855), https://www.cellsignal.cn/products/primary-antibodies/phospho-4e-bp1-thr37-46-236b4-rabbit-mab/2855;
4E-BP1(Cell Signaling Technology,#9644), https://www.cellsignal.cn/products/primary-antibodies/4e-bp1-53h11-rabbit-mab/9644;
Phospho-mTOR (Ser2448)(Cell Signaling Technology,#5536), https://www.cellsignal.cn/products/primary-antibodies/phospho-mtor-ser2448-d9c2-xp-rabbit-mab/5536;
mTOR(Cell Signaling Technology,#2983), https://www.cellsignal.cn/products/primary-antibodies/mtor-7c10-rabbit-mab/2983;
Phospho AKT (Thr308)(Cell Signaling Technology,#9275), https://www.cellsignal.cn/products/primary-antibodies/phospho-akt-thr308-antibody/9275;
AKT(Santa Cruz,#SC-5298), https://www.scbt.com/zh/p/akt1-antibody-b-1;
Phospho ERK1/2 (T202/Y204) (Cell Signaling Technology,#4370S), https://www.cellsignal.cn/products/primary-antibodies/phospho-p44-42-mapk-erk1-2-thr202-tyr204-d13-14-4e-xp-rabbit-mab/4370
ERK2(Santa Cruz,#SC-1647), https://www.scbt.com/zh/p/erk-2-antibody-d-2;
ERK1(Santa Cruz,#SC-93), https://www.scbt.com/zh/p/erk-1-antibody-c-16;
Phospho MEK1/2 (Ser217/221)(Cell Signaling Technology,#9121), https://www.cellsignal.cn/products/primary-antibodies/phospho-mek1-2-ser217-221-antibody/9121
MEK2(Cell Signaling Technology,#9125), https://www.cellsignal.cn/products/primary-antibodies/mek2-antibody/9125;
Phospho SAPK/JNK (Thr183/Ty185)(Cell Signaling Technology,#9251), https://www.cellsignal.cn/products/primary-antibodies/phospho-sapk-jnk-thr183-tyr185-antibody/9251;
SAPK/JNK (Cell Signaling Technology,#9252), https://www.cellsignal.cn/products/primary-antibodies/sapk-jnk-antibody/9252
Myc tag(Santa Cruz,#SC-40), https://www.scbt.com/zh/p/c-myc-antibody-9e10;
FLAG tag(Proteintech,#66008-3-IG), https://www.ptglab.com/products/Flag-tag-Antibody-66008-3-Ig.htm;
Ubiquitin(Cell Signaling Technology,#3936), https://www.cellsignal.cn/products/primary-antibodies/ubiquitin-p4d1-mouse-mab/3936;
GFP tag(Proteintech,#50430-2-AP), https://www.ptglab.com/products/eGFP-Antibody-50430-2-AP.htm
HA tag(Proteintech,#66006-2-IG), https://www.ptglab.com/products/HA-Tag-Antibody-66006-2-Ig.htm
ZHX2(Genetex,#GTX112232), https://www.genetex.cn/Product/Detail/ZHX2-antibody-C1C3/GTX112232;
NEK7 (Bethyl Lab Inc.,#A302-684A), https://www.fortislife.com/products/primary-antibodies/rabbit-anti-nek7-antibody/BETHYL-A302-684
STAT3(Cell Signaling Technology,#9139), https://www.cellsignal.cn/products/primary-antibodies/stat3-124h6-mouse-mab/9139;
Phospho STAT3 (Tyr705)(Cell Signaling Technology,#9145), https://www.cellsignal.cn/products/primary-antibodies/phospho-stat3-tyr705-d3a7-xp-rabbit-mab/9145;
JAK2(Santa Cruz,#sc-390539), https://www.scbt.com/zh/p/jak2-antibody-c-10;
Ki67(Abcam,#ab15580), https://www.abcam.cn/products/primary-antibodies/ki67-antibody-ab15580.html;
Cleaved Caspase-3(Cell Signaling Technology,#9579), https://www.cellsignal.cn/products/primary-antibodies/cleaved-caspase-3-asp175-d3e9-rabbit-mab/9579;

Eukaryotic cell lines

Policy information about cell lines and Sex and Gender in Research

Cell line source(s)	786-O, A498, Caki-2 and 293T cell lines were purchased from ATCC (Manassas, VA).
Authentication	NO
Mycoplasma contamination	All cell lines were tested negative of mycoplasma contamination.
Commonly misidentified lines (See ICLAC register)	NO

Animals and other research organisms

Policy information about studies involving animals; ARRIVE guidelines recommended for reporting animal research, and Sex and Gender in Research

Laboratory animals	6-week-old SCID mice were used for kidney cancer xenograft study as described in the Methods section. All mice were housed under standard conditions at room temperature with a 12 h light/dark cycle with access to food and water ad libitum and maintained under pathogen-free conditions. The animal study was approved by the Institutional Animal Care and Use Committee (IACUC) at the Mayo Clinic.
Wild animals	No
Reporting on sex	Both male and female mice were used in this study.
Field-collected samples	No
Ethics oversight	The animal study was approved by the Institutional Animal Care and Use Committee (IACUC) at the Mayo Clinic.

Note that full information on the approval of the study protocol must also be provided in the manuscript.

Plants

Seed stocks	N/A
Novel plant genotypes	N/A
Authentication	N/A
