# Supplementary material for: SPOP-mediated ZMYND8 ubiquitination and phase separation exclusion drives mTOR inhibitor resistance in kidney cancer
Source: Nat Commun. 2026 Aug 25;17:10147. doi: 10.1038/s41467-026-77043-9 (PMC13601609; doi:10.1038/s41467-026-77043-9)
Supplement: Supplementary file 3 [file 41467_2026_77043_MOESM3_ESM.pdf]

nature portfolio

Peer Review File

SPOP-mediated ZMYND8 ubiquitination and phase separation exclusion drives mTOR inhibitor resistance in kidney cancer

Corresponding Author: Professor Haojie Huang

This file contains all reviewer reports in order by version, followed by all author rebuttals in order by version.

Version 0:

Reviewer comments:

Reviewer #1

(Remarks to the Author)
In this paper, the authors revealed a mechanism that SPOP recognizes and binds to ZMYND8 through its substrate-binding MATH domain, and induces non-degradable K63-linked polyubiquitination of ZMYND8 at K398, which inhibits ZMYND8 phase separation and promotes the formation of transactivation ZMYND8-ZHX2 complex, leading to transcriptional activation of NEK7. And, the authors identified NEK7 as an alternative kinase for phosphorylation of p70S6K independent of the Akt-mTOR signaling. Aberrant upregulation and activation of NEK7 results in ccRCC cell resistance to mTOR inhibitors. Further, they designed PROTAC targeting NEK7 and revealed SJW-8136 as the most efficient degrader. Combination of SJW-8136 and mTOR inhibitors showed increased tumor sensitivity to mTOR inhibitors and efficient inhibition of tumor growth in ccRCC. Overall, these findings are both interesting and significant for understanding oncogene transcription and drug resistance, and provide a new treatment strategy for ccRCC. The paper is extremely thorough and warrants publication upon resolution of the follow concerns.

Major points:
1) Fig 1: the authors claim that “SPOP overexpression promotes mTOR inhibitor resistance in ccRCC via K63-linked polyubiquitination of ZMYND8 at lysine-398”. To really understand the effect of K63-linked polyubiquitination of ZMYND8 at lysine-398 on mTOR inhibitor resistance, authors should use this K398R mutant that disrupts ZMYND8 K63-linked polyubiquitination to show the effect on mTOR inhibitor resistance. These can be assessed after ZMYND8 WT/ K398R rescue in the KO cells. Furthermore, the authors should show the polyubiquitination of ZMYND8 is indeed directly modified by SPOP.
2) Fig 2: the authors reveal that SPOP inhibits ZMYND8 to form liquid compartments. What is the functional consequence of this regulatory mechanism? As ZMYND8 recruits KDM5C histone demethylase to restrict enhancer overactivation and suppress tumorigenesis, while promotes tumorigenesis by recruiting BRD4 or EZH2 to positively regulate gene expression, whether SPOP preventing ZMYND8 from forming phase separation can convert this corepressor (e.g., by restricting enhancer overactivation) into a transcriptional coactivator (e.g. by interaction with BRD4 or EZH2)?
3) Fig 4: the authors reveal that the protein expression of NEK7 is closely associated with p70S6K phosphorylation, but failed to show NEK7 is indeed a direct kinase responsible for phosphorylation of p70S6K.

Minor points:
1) Line 454-455, why the authors discussed STAT3, please describe clearly.

Reviewer #2

(Remarks to the Author)
Review Statement:
This paper demonstrates both biological and mechanistic novelty, addressing the problem with the use of recent techniques. It is packed with a wealth of relevant information, with every sentence serving a purpose without redundancy. The experiments conducted to address the questions are precise, comprehensive, and carried out with exceptional attention to detail. This study began with the novel observation that, despite SPOP being involved in ZMYND8 degradation, it does not alter ZMYND8 mRNA levels, protein expression, or lifetime. Building on this finding, the authors revealed that SPOP-

mediated K63-linked polyubiquitination of ZMYND8 drives aberrant NEK7 transcription and mTOR inhibitor-resistant growth, identifying NEK7 as a promising therapeutic target. The successful application of a NEK7-targeting PROTAC to overcome everolimus resistance further underscores the translational potential of these findings.
Several years ago, many reports on biomolecular condensates remained focused on observational phenomena and theoretical hypotheses at the highest conceptual level. Only recently has emerging evidence started to reveal the highly complex regulatory nature of these so-called LLPS bodies, demonstrating that some condensates are functionally active while others are inhibitory. This paper goes beyond merely reporting the LLPS phenomenon of ZMYND8 and instead establishes underlying principles. The authors identify the key amino acid residue critical for ZMYND8 condensate formation, elucidating the dynamics of LLPS and verifying the downstream effects driven by its inhibitory function.
Overall, I am positive about the publication of this paper in Nature Communications. My major comments are limited to biophysical and technical aspects related to LLPS, and I would like to clarify that these suggestions are made to support and strengthen the study.

Major comments:
1. In Figures 2C, 2E, 2G, and Fig. S3B, the procedure for quantifying the number of puncta is not explained. While the “Immunofluorescent chemistry (IFC) and quantification” section in the Materials and Methods describes the experimental method, it lacks the image data quantification process. If this omission was unintentional, the authors should provide a detailed explanation. If the puncta were simply counted manually based on prominent features observed visually, I recommend using free software such as ImageJ (Fiji) to quantify blobs with an arbitrary threshold intensity above the background for more rigorous and reproducible analysis.

2. The paper “ZMYND8 mediated liquid condensates spatiotemporally decommission the latent super-enhancers during macrophage polarization, Pan Jia et al., Nat Comm 2021” has already established the LLPS properties of ZMYND8, so it is reasonable to assume that ZMYND8 blobs possess liquid-like characteristics. However, methods to verify LLPS in live cells, such as capturing fusion events, FRAP (fluorescence recovery after photobleaching), in vitro droplet assays, or treatment with 1,6-hexanediol, have become well-established. To confirm the LLPS nature, I suggest including experimental evidence from at least two of these methods. While it may be challenging to perform these experiments using fixed samples with IF, an alternative approach could involve using an overexpression vector for live-cell experiments. If ZMYND8 overexpression is not feasible, the authors could cite and elaborate on Pan Jia et al.'s 2021 paper to clarify the LLPS properties of ZMYND8 instead.

Minor comments:
1. While the results section describes the relationship between mTOR and NEK in ccRCC development and drug resistance, adding a paragraph in the introduction to explain this connection would help readers better understand the context of the study.

2. In the sentence, “We found that knockout of ZMYND8, a chromatin reader but not other nine top hits largely increased everolimus sensitivity in 786-O (Fig. 1E and Supplementary Fig. 1J),” it is unclear why ZMYND8, as a chromatin reader, makes cells more sensitive to everolimus. Clarification on this point would strengthen the interpretation.

3. The statement “Treatment with 1,6-hexanediol, a potent inhibitor of LLPS, …” is not entirely accurate. It would be more precise to specify that 1,6-hexanediol is particularly effective against LLPS driven by hydrophobic interactions. Adding this clarification would improve the scientific accuracy of the text.

Reviewer #3

(Remarks to the Author)
In this study, the authors reported that SPOP promotes K63-linked polyubiquitination of ZMYND8 for inhibiting ZMYND8 to form phase separation compartments and drives the formation of ZMYND8-ZHX2 transactivation complex, resulting in aberrant NEK7 kinase gene transcription, alternative activation of p70S6K, and mTOR inhibitor-resistant cell growth. The authors further demonstrated that inhibition of SPOP or NEK7 increased ccRCC cell sensitivity to mTOR inhibitor. Treatment with a NEK7 PROTAC inhibited aberrant p70S6K activation and overcame everolimus resistance in ccRCC cells in vitro and in vivo. This study identified a function switch of ZMYND8 driven by SPOP as a key mechanism that causes mTOR inhibitor resistance and suggested NEK7 as a potential target of increasing mTOR inhibitor sensitivity in ccRCC. Overall, this is a well-designed and thoroughly conducted study to demonstrate the crucial function of SPOP in promoting mTOR inhibitor resistance of ccRCC tumor. However, several minor concerns remain to be investigated before publication.
1. The authors claimed that NEK7 degradation mitigated mTOR inhibitor resistance of ccRCC tumor cells, a mTOR inhibitor-resistant cell line should be used to validate this conclusion.
2. The NEK7 PROTAC was used for combination with mTOR inhibitor in vivo, the administration procedure should be provided in the method section, PK values of the NEK7 PROTAC should be evaluated.
3. The maximal degradation concentration of the NEK7 PROTACs should be quantified.
4. The advantage of the NEK7 PROTACs over the inhibitors should be discussed.
5. NEK7 expression in the tumor tissue upon NEK7 PROTAC treatment should be evaluated to validate NEK7 eradication in vivo.

Reviewer #4

(Remarks to the Author)
Tang et al. show that SPOP promotes non-degradable K63-linked ubiquitination of ZMYND8 at lysine 398 in human ccRCC cell lines. The ubiquitinated ZMYND8 inhibits the formation of phase separation compartments, but promotes the transcription of NEK7 by forming a transactivation complex with ZHX, leading to activation of p70S6K and growth resistance to the mTOR inhibitor everolimus. Inhibition of either SPOP or NEK7 enhances the growth inhibitory effect of everolimus. Also, both in vitro and in vivo studies show that inhibition of NEK7 can suppress aberrant p70S6K activation and overcome everolimus resistance. The research reveals that the SPOP-ZMYND8-NEK7-p70S6K axis plays a critical role in ccRCC resistance to mTOR inhibitors. The finding is novel and interesting. However, before the manuscript is suggested for publication in the journal, some concerns need to be addressed.

Major concerns:
1) Full activation of p70S6K is determined by its phosphorylation on multiple sites within the catalytic, linker, and pseudosubstrate domains. While the phosphorylation of Thr229 (primarily by PDK1) in the catalytic domain and Thr389 (primarily by mTOR) in the linker domain is most crucial for the kinase activity, the phosphorylation on Ser411, Thr421, and Ser424 in the pseudosubstrate domain is also important for the activation of p70S6K. Fig. 3 and Fig. S4 show that SPOP-mediated ZMYND8 ubiquitination promotes ccRCC cell proliferation and Thr389 phosphorylation of p70S6K independently of mTOR. Given the availability of antibodies to phospho-p70S6K on Thr412 and Thr229, it is good to determine whether the phosphorylation of p70S6K on Thr412 and Thr229 is altered. The result may advance our understanding of why SPOP-promoted cell proliferation is mTOR-independent.
2) It has been shown that NEK6 and NEK7 can phosphorylate p70S6K on Thr412 (PMID: 11516946; PMID: 12589797). Fig. 4 and Fig. S5 show that the SPOP-mediated polyubiquitination of ZMYND8 upregulates the transcription of NEK7, consequently increasing Thr389 phosphorylation of p70S6K. It is unclear whether NEK7 impacts the phosphorylation of p70S6K on Thr412 and Thr229 in ccRCC cells. This piece of data may help understand why inhibition of NEK7 can enhance the anticancer activity of everolimus (mTOR inhibitor).
3) It has been shown that treatment with everolimus at low micromolar concentrations (~1 μM) can potently inhibit the phosphorylation of p70S6K in 786-O cells (PMID: 23571736; PMID: 30771617). However, Fig. 6A and Fig S7A show that treatment with everolimus at a higher concentration (5 μM) only moderately inhibits the phosphorylation of p70S6K (Thr389) in the cells. Please have a discussion on the discrepancy.
4) Fig. 6J shows that treatment with SJW-8136 (NEK 7 degrader) moderately reduces the level of NEK7 in 786-O cells, compared to treatment with sgNEK7 (Fig. 6A). However, SJW-8136 and sgNEK7 can enhance the anticancer activity of everolimus at a similar level, comparing Fig. 6B, C, E, F and G to Fig. M, N, O, P and Q. The results suggest that SJW-8136 be not a selective degrader of NEK7. In addition to inhibiting the phosphorylation of Stat3 (Tyr705), SJW-8136 may target more proteins critical for cell proliferation and tumor growth. If there were more convincing data available, it would be better to soften the tone, regarding the specific effect of SJW-8136 on NEK7 degradation.
5) It is suggested to have semi-quantification analyses for all blots, which can be shown as “Supplemental data”.

Minor Concerns:
1) Please define all abbreviations at the first appearance, such as BRD4, SPOP, AR, ERG, ZHX2, p70S6K, etc.
2) Please provide more detailed information about how each experiment was performed in the figure legends. For example, how long time were the cells treated with compounds for the MTS assay, colony formation assay, and Western blotting?
3) Please carefully check whether the descriptions in the figure legends are correct. For instance, “WB analysis of SPOP protein level in 786-O cells transfected with indicated plasmids” (Fig. S1B) vs. “786-O cells infected with lentivirus for sgControl or sgSPOP…” (Fig. 1A).
4) It is good to provide uncropped western blots (raw data) for related figures, which can be shown as “Source data”.

Reviewer #5

(Remarks to the Author)
Cullin-RING ligases (CRL) play broad and essential roles in gene expression and tissue homeostasis. Not surprisingly, CRL dysfunction can lead to a wide range of disease pathologies including cancer. In particular, the CRL CUL3SPOP has been shown to function as a tumor suppressor in many solid tumor types, including cancer of the prostate, endometrium, and breast (Yang and Zhu, 2022). On the other hand, SPOP is highly expressed in clear cell renal cell carcinomas (ccRCC), the most prevalent type of kidney cancer, where it is believed to function as an oncogene (Zhang et al., 2023). Thus, determining the molecular mechanisms by CUL3SPOP regulates tumor formation is important for developing new cancer treatments as well as overcoming resistance to existing chemotherapeutics.

Tang et al. seek to elucidate the role of the CRL, CUL3SPOP, in driving ccRCC and promoting its resistance to current treatments, namely mTOR inhibitors everolimus and temsirolimus. The authors show that SPOP ubiquitinates ZMYND8 within its intrinsically disordered region at lysine 398, inhibiting phase separation of ZMYND8. Ubiquitinated ZMYND8 forms a complex with ZHX2 to induce the expression of NEK7, which phosphorylates p70S6K in an mTOR-independent manner. This ultimately leads to cancer cell proliferation and explains why mTOR inhibition would have no effect. The authors have also made the novel finding that the small molecule SJW8136 is a NEK7 inhibitor and have shown that SJW8136 reduces the phosphorylation levels of p70S6K and enhances the sensitivity of cancer cells towards everolimus.

The manuscript can be accepted for publication after the following points have been addressed:

Major issues:

1. Several areas of the manuscript need to be revised to prevent over interpretation of the results:
a. The last sentence of the introduction, "This ubiquitination event acts as a molecular switch...mTOR inhibitor resistance in ccRCC." is a very direct statement and can be revised to be more descriptive instead.
b. In the third section of Results (SPOP-mediated ZMYND8 ubiquitination..), first paragraph, last sentence - "…suggesting a functional switch of ZMYND8.." seems like an oversimplification of the authors' findings.
c. Same section as above, last paragraph, last sentence, "These data suggest that SPOP-mediated ubiquitination of ZMYND8 promotes p70S6K phosphorylation…". Perhaps this could be adjusted to "ZMYND8 is needed/required for p70S6K phosphorylation"?
d. Discussion section, first paragraph, last sentence - "assembly of the ZMYND8-ZHX2 transactivation complex that promotes expression of oncogenes...". The authors have only found the altered expression of NEK7, hence, this sentence seems to be an overstatement.

2. In Fig. 1, the Flag-ZMYND8 immunoprecipitations performed with HA-Ub are done under native conditions. Thus, the detected polyubiquitin signal could arise from SPOP substrates that may be associated with ZMYND8 rather than ZMYND8 itself. To distinguish this, can the authors either: (1) perform a denaturing His-Ub pulldown and blot for Flag-ZMYND8 or (2) look for the presence of a high-molecular weight smear using anti-FLAG antibody in Fig. 1I or 1J?

3. For Figure 3H, can the authors include a positive control for mTOR inhibition to ensure that repression of this pathway in 786-O cells indeed leads to loss of p70S6K phosphorylation?

4. Comparing Figures 1B and S1G, it seems that SPOP inhibitor is causing more cell death as compared to knockdown of SPOP. Please include appropriate controls (i.e. treatment of CAKI2 cells with SPOP inhibitor) to account for any pleiotropic effects.

5. In vitro reconstitution assays are needed to support the claim that ZMYND8 is a substrate of SPOP. At a minimum, the authors need to show direct binding between purified recombinant ZMYND8 and SPOP. However, an in vitro ubiquitination reaction would work best to address this point.

6. In Figure S2A, failure to detect interaction between MYC-SPOPΔMATH and Flag-ZMYND8 could arise due to gross misfolding of the deletion mutant. Can the authors either provide a reference that supports the structural integrity of the ΔMATH mutant, or, perform the same assay via point mutation of the MATH domain?

7. The potential reason for C-terminal 1088-GSSSS-1092 motif of ZMYND8 being critical for SPOP binding should be included in the discussion. Can this motif be targeted for therapeutic purposes?

8. In Figures 2D and E, the image shown does not appear to match the quantification.

9. Addition of 1,6-hexanediol seems to cause significant changes in nuclear morphology (Fig. 2H). A negative control (sgCtrl) cell line treated with 1,6-HD should be included to ensure these effects are not specific to sgSPOP cells. A discussion should be included if there are any known intranuclear effects of 1,6-HD.

10. Figure 4 – Can the authors assess the level of phosphorylated p70S6k in NEK7 and ZMYND8 double knockouts, to confirm that their relationship is epistatic? If the double knockout reveals a synergistic effect, this would not necessarily invalidate the authors' model, but it would be important to know the relative magnitude of each gene's contribution.

11. In addition to Figures S5 C, D it would be useful to know how levels of phosphorylated p70S6K compare in ccRCC versus normal tissue (if such data are available). I believe this would strengthen the claim of the authors of using SJW-8136 as a potential therapeutic molecule.

12. Are there any known roles of SPOP in mediating nondegradative ubiquitination of proteins other than ZMYN8 and, if so, can the authors comment on how their observations compare with these other findings?

Minor issues:

1. Cited references are not matching with the text in certain places. For example, in the second paragraph of the Introduction ("…serves as a reader of the dual histone mark H3K4me1/H3K14ac4."), this reference should be "Li N et al., Mol Cell, 2016".

2. The authors have used multiple cell lines throughout their work. To help the reader and avoid confusion, all figures should be labeled with its respective cell line (not just in the figure legend). For example, none of the blots in Fig. 1 using 293T cells are labeled as such.

3. In Fig. 1C and D, a Western blot showing SPOP overexpression for these experiments is missing.

4. Please include the molecular weight standard for western blot panels. This is particularly important for polyubiquitin signals that should be larger in size compared to FLAG-ZMYND8 (Fig. 1I, 1J, 1L, 1M, 1P, S2H, S8E).

5. Figure labeling needs to be checked. For example, Fig. S7E should be labeled "NEK7" instead of "SPOP".

6. Methods section does not include information on protocol followed for quantification of ZMYND8 puncta. Are puncta defined based on size or fluorescence intensity?

Tang et al. seek to uncover the molecular basis by which CUL3SPOP promotes prostate cancer and its resistance to mTOR inhibitors, a class of small molecule that is used as a second-line treatment for this cancer type. The authors' data are generally of high quality and their claims supported by experimental evidence. One of the authors' major findings, that ZMYND8 may be a novel substrate of CUL3SPOP, needs to be substantiated with additional experiments to rule out the possibility that its ubiquitylation might occur indirectly (i.e. possible contribution of other E3, coadaptors, or substrates). If this cannot be reconciled, then the text will need to be adjusted accordingly to allow consideration for alternative explanations. Overall, this manuscript would be of significant interest to the scientific community and I would support publication provided the above corrections can be incorporated.

Version 1:

Reviewer comments:

Reviewer #1

(Remarks to the Author)
The authors have done extensive revisions to address the prior comments and I would recommend the publication of the paper.

Reviewer #2

(Remarks to the Author)
The authors have fully addressed the concerns I previously raised. They now provide rigorous evidence and clear explanations regarding the liquid properties of ZMYND8 under various conditions, leaving no remaining ambiguities. The revision has markedly improved the overall clarity of the article. I recommend publication of this manuscript in Nature Communications.

Reviewer #3

(Remarks to the Author)
The authors had sufficiently and correctly responded to all my comments, I prefer to recommend acceptance for publication at current stage.

I checked the procedure for PROTAC synthesis in the supporting information file, all the synthesis protocol and the reaction condition are reasonable. Therefore I recommend acceptance for publication.

Reviewer #4

(Remarks to the Author)
The manuscript has been well revised.

Reviewer #5

(Remarks to the Author)
Cullin-RING ligases (CRLs) are critical regulators of gene expression and tissue homeostasis, and their dysfunction is implicated in diverse disease pathologies, including cancer. Among them, the CUL3-SPOP complex has been shown to act as a tumor suppressor in prostate, endometrial, and breast cancers, but intriguingly functions as an oncogene in clear cell renal cell carcinoma (ccRCC). In this study, Tang et al. uncover a novel mechanism by which CUL3-SPOP drives ccRCC progression and resistance to mTOR inhibitors, demonstrating that SPOP ubiquitinates ZMYND8 at lysine 398, thereby disrupting its phase separation and enabling complex formation with ZHX2 to induce NEK7 expression. This leads to mTOR-independent phosphorylation of p70S6K, providing a compelling explanation for the failure of mTOR-targeted therapies, while the identification of the NEK7 inhibitor SJW8136 highlights a potential therapeutic strategy to restore sensitivity to everolimus. Overall, this work is of high quality, its claims well supported by the data, and the findings will be of broad interest to the cancer biology community.

The authors have satisfactorily responded to all of our queries and concerns. One error that should be addressed is in Figures 2D and E where in the text the authors mention using Caki-2 ZMYND8-KO cells. However, the figure is labeled as 293T cells. A thorough proofreading will be critical to avoid these errors. We recommend this manuscript to be accepted for publication following this minor revision.

Reviewer #6

(Remarks to the Author)

I co-reviewed this manuscript with one of the reviewers who provided the listed reports. This is part of the Nature Communications initiative to facilitate training in peer review and to provide appropriate recognition for Early Career Researchers who co-review manuscripts.

Open Access This Peer Review File is licensed under a Creative Commons Attribution 4.0 International License, which permits use, sharing, adaptation, distribution and reproduction in any medium or format, as long as you give appropriate credit to the original author(s) and the source, provide a link to the Creative Commons license, and indicate if changes were made.
In cases where reviewers are anonymous, credit should be given to 'Anonymous Referee' and the source.
The images or other third party material in this Peer Review File are included in the article's Creative Commons license, unless indicated otherwise in a credit line to the material. If material is not included in the article's Creative Commons license and your intended use is not permitted by statutory regulation or exceeds the permitted use, you will need to obtain permission directly from the copyright holder.
To view a copy of this license, visit https://creativecommons.org/licenses/by/4.0/

Authors' Response to Reviewers' Comments on NCOMMS-24-78902

We very much thank the Editor for giving us the opportunity to revise the manuscript.

REVIEWER COMMENTS

Reviewer #1 (Remarks to the Author):

In this paper, the authors revealed a mechanism that SPOP recognizes and binds to ZMYND8 through its substrate-binding MATH domain, and induces non-degradable K63-linked polyubiquitination of ZMYND8 at K398, which inhibits ZMYND8 phase separation and promotes the formation of transactivation ZMYND8-ZHX2 complex, leading to transcriptional activation of NEK7. And, the authors identified NEK7 as an alternative kinase for phosphorylation of p70S6K independent of the Akt-mTOR signaling. Aberrant upregulation and activation of NEK7 results in ccRCC cell resistance to mTOR inhibitors. Further, they designed PROTAC targeting NEK7 and revealed SJW-8136 as the most efficient degrader. Combination of SJW-8136 and mTOR inhibitors showed increased tumor sensitivity to mTOR inhibitors and efficient inhibition of tumor growth in ccRCC. Overall, these findings are both interesting and significant for understanding oncogene transcription and drug resistance, and provide a new treatment strategy for ccRCC. The paper is extremely thorough and warrants publication upon resolution of the follow concerns.

Reply: We thank the Reviewer for the positivity and support of publication of our manuscript in Nature Communications. All the suggestions and concerns of the Reviewer have been fully addressed as outlined below.

Major points:

1) Fig 1: the authors claim that "SPOP overexpression promotes mTOR inhibitor resistance in ccRCC via K63-linked polyubiquitination of ZMYND8 at lysine-398". To really understand the effect of K63-linked polyubiquitination of ZMYND8 at lysine-398 on mTOR inhibitor resistance, authors should use this K398R mutant that disrupts ZMYND8 K63-linked polyubiquitination to show the effect on mTOR inhibitor resistance. These can be assessed after ZMYND8 WT/ K398R rescue in the KO cells. Furthermore, the authors should show the polyubiquitination of ZMYND8 is indeed directly modified by SPOP.

Reply: We thank the Reviewer for the excellent point. We rescued ZMYND8 WT and ZMYND8 K398R in ZMYND8-KO 786-O cells. Our new data showed that restoration of ZMYND8 WT, but not K398R mutant reversed ZMYND8 KO-induced increased response to everolimus in 786-O cells (**Review Figure 1 and Figure S4D**).

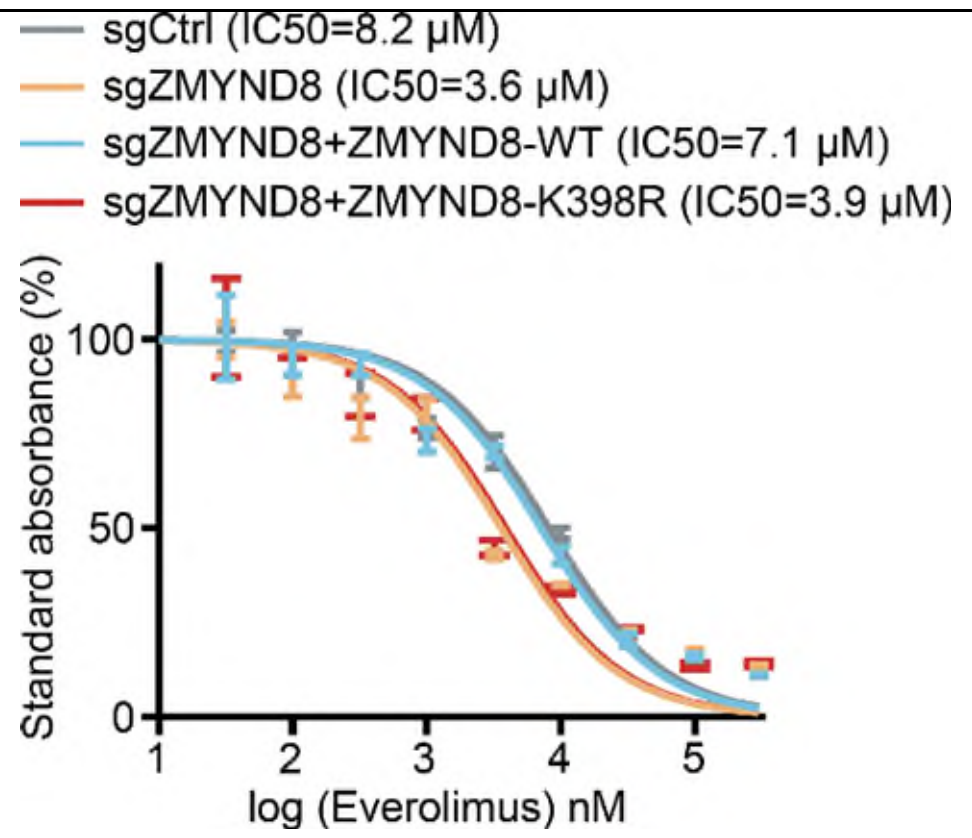

Review Figure 1. 72h IC50 of everolimus in 786-O cells transfected with indicated sgZMYND8 and/or rescued ZMYND8. Data are shown as mean ± SD (n=3).

To determine whether the polyubiquitination of ZMYND8 is indeed directly modified by SPOP, we performed in vitro polyubiquitination assay using purified proteins and the method as we reported previously (Mol Cell 2015, PMID 26344096). Our new data showed that only in the presence of SPOP, CUL3-RBX1 protein complex was able to promote polyubiquitination of purified ZMYND8 in vitro (**Review Figure 2 and Figure S2E**).

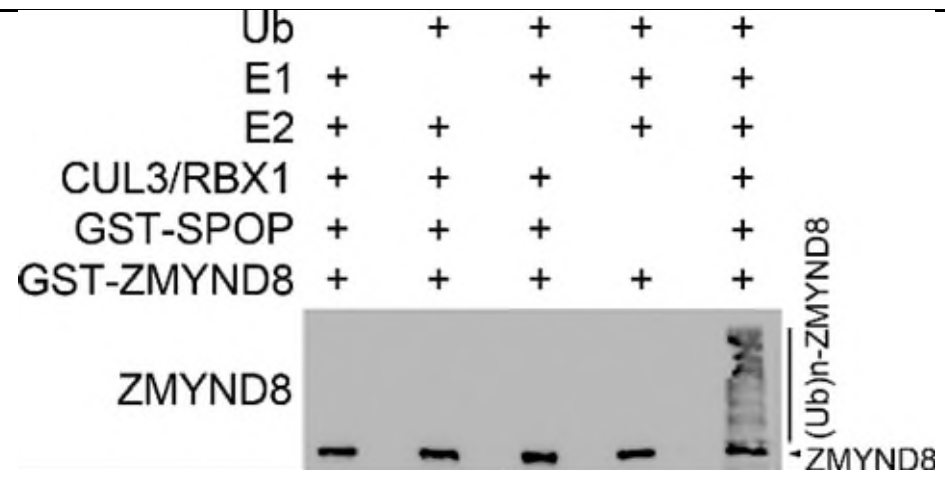

Review Figure 2. WB analysis of the products of in vitro ubiquitination assay performed by incubating the reconstituted E3 Ub ligase complex (CUL3-RBX1, GST-SPOP) with E1, E2, Ub, and GST-ZMYND8 at 30°C for 2 hr.

2) Fig 2: the authors reveal that SPOP inhibits ZMYND8 to form liquid compartments. What is the functional consequence of this regulatory mechanism? As ZMYND8 recruits KDM5C histone demethylase to restrict enhancer overactivation and suppress tumorigenesis, while promotes tumorigenesis by recruiting BRD4 or EZH2 to positively regulate gene expression, whether SPOP preventing ZMYND8 from forming phase separation can convert this corepressor (e.g., by restricting enhancer overactivation) into a transcriptional coactivator (e.g. by interaction with BRD4 or EZH2)?

Reply: To address these questions, we used co-IP experiments to clarify the interactions between ZMYND8, KDM5C, and EZH2. We found that overexpression of SPOP inhibited the interaction between ZMYND8 and KDM5C (**Review Figure 3A**) but slightly enhanced the interaction between ZMYND8 and EZH2 (**Review Figure 3B**).

These new data supported the hypothesis that SPOP inhibition of ZMYND8 phase separation results in a shift in the protein profile associated with ZMYND8, shifting its binding from corepressor to coactivator. However, we have to admit that a large-scale profiling, including mass spectrometry-based protein profiling or ChIP-seq are warranted in the future to systematically dissect whether SPOP prevention of ZMYND8 from forming phase separation can convert the corepressor (e.g., by restricting enhancer overactivation) into a transcriptional coactivator (e.g. by interaction with BRD4 or EZH2).

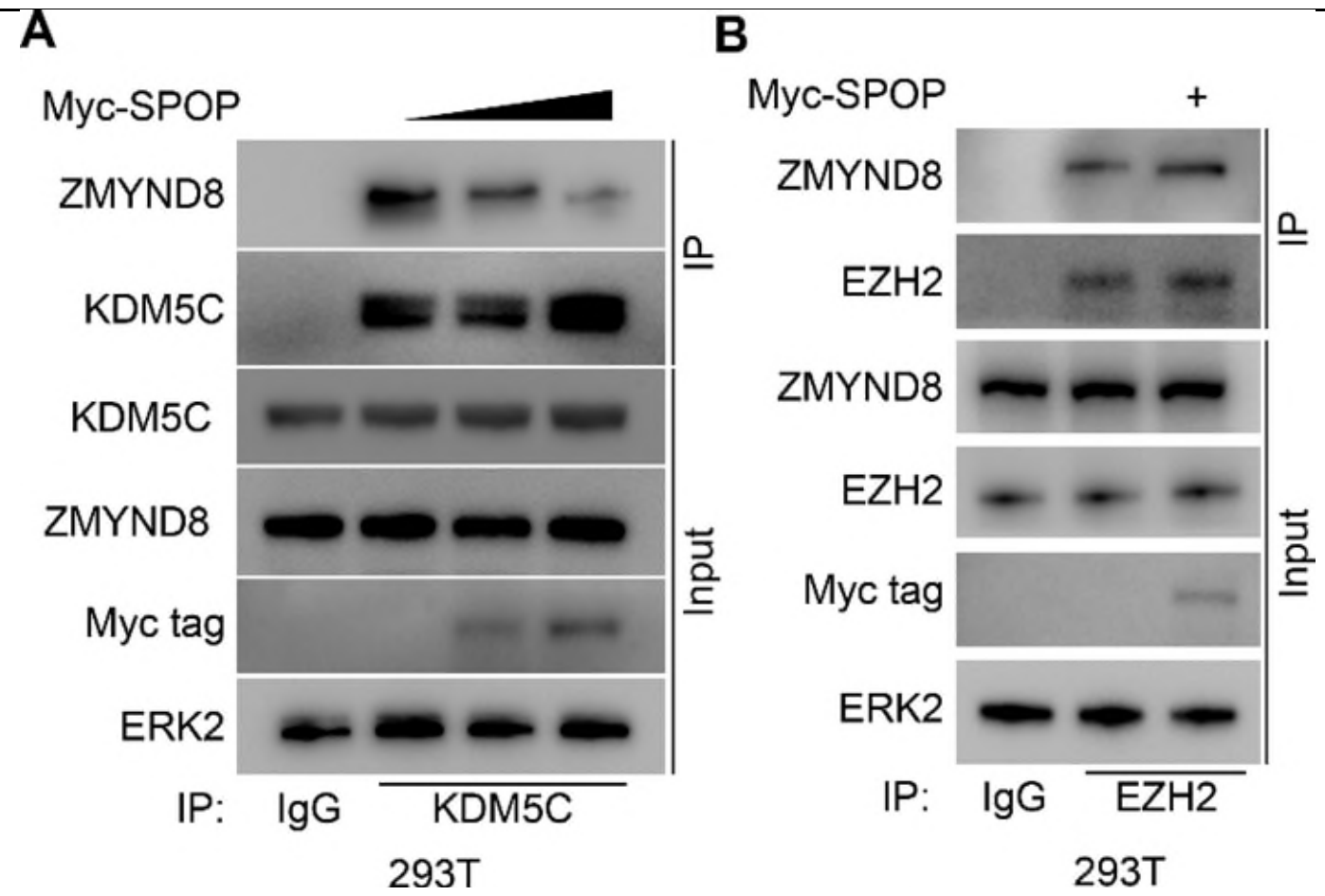

Review Figure 3. WB analysis of ZMYND8-KDM5C interaction (A) and ZMYND8-EZH2 interaction (B) in co-IP samples from 293T cells transfected with indicated plasmids.

3) Fig 4: the authors reveal that the protein expression of NEK7 is closely associated with p70S6K phosphorylation, but failed to show NEK7 is indeed a direct kinase responsible for phosphorylation of p70S6K.

Reply: Thank the Reviewer for the great point. We agree with the Reviewer that direct phosphorylation of P70S6K by NEK7 is undoubtedly crucial for our study. In previously published articles, Edmarcia Elisa de Souza et. al. has described in very details that NEK7 directly regulates P70S6K phosphorylation in vitro (J Proteome Res. 2014, PMID 25093993). Given that it is known in literature that NEK7 can directly phosphorylate P70S6K in vitro, we did not repeat the in vitro kinase assay. However, to directly show the evidence, we included the result from the published article demonstrating NEK7 directly phosphorylates P70S6K (**Review Figure 4**).

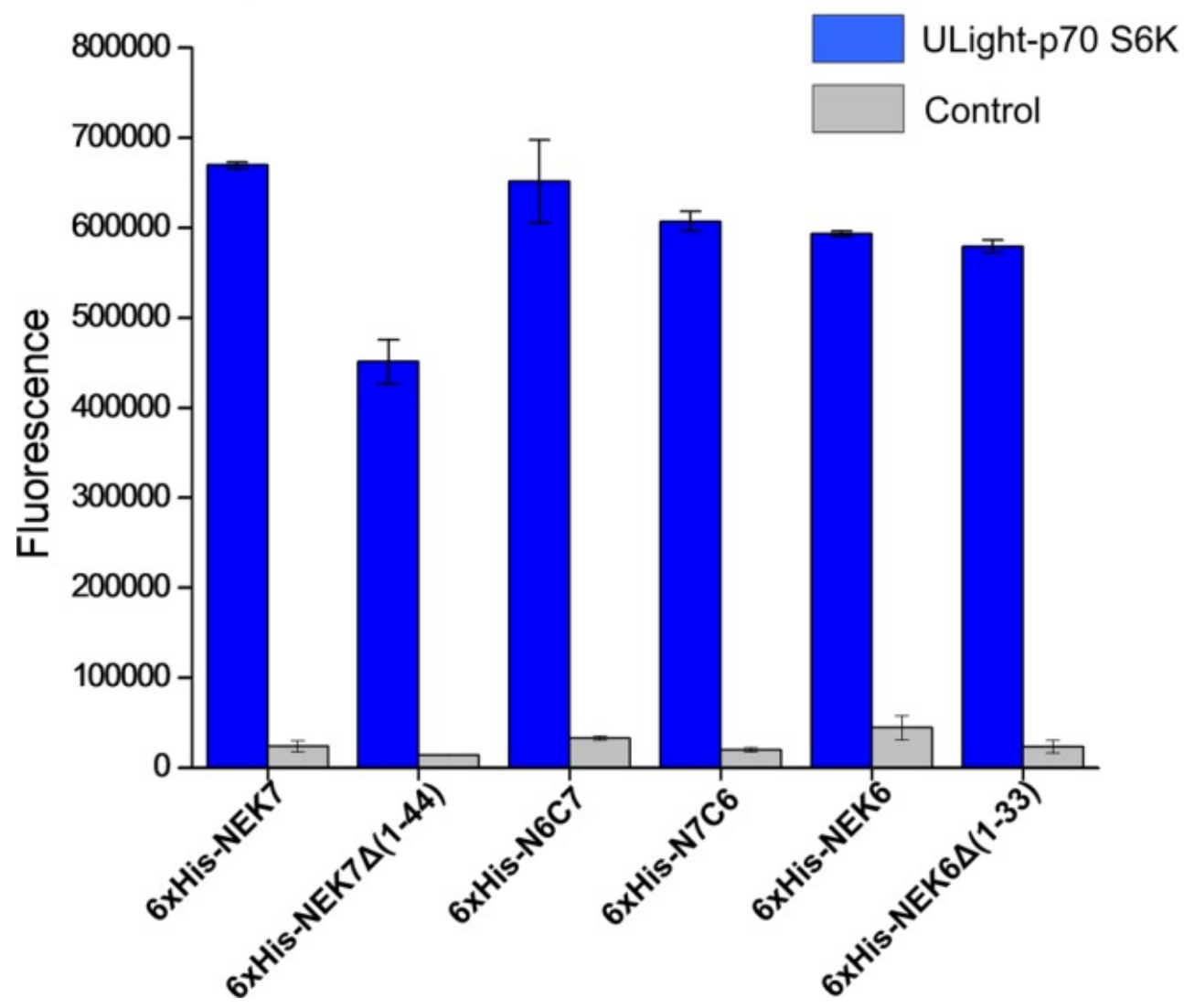

Review Figure 4. Fig 7 in J Proteome Res. 2014 Aug 5;13(9):4074-4090. In vitro kinase activity assay of full-length NEK7 and other truncations.

Minor points:

1) Line 454-455, why the authors discussed STAT3, please describe clearly.

Reply: This is an excellent point. The reason we mentioned STAT3 in the manuscript is that TG101348, a compound reported to bind to NEK7 (Med chem comm 2017, PMID 30108900), has also been reported to inhibit the enzyme activity of JAK2, thereby indirectly inhibiting STAT3 phosphorylation (J Clin Oncol 2011, PMID 21220608). Therefore, when we utilized TG101348 as a binding ligand of NEK7 to synthesize NEK7-targeting PROTACs, we aimed to define PROTACs that can specifically target NEK7, but not JAK2 for protein degradation. To this end, we ultimately identified NEK7-targeting PROTAC SJW-8136, which only degraded NEK7, but had little or no activity on JAK2 degradation given that it did not interference with STAT3 phosphorylation. This is why we ultimately selected this compound for subsequent experiments. We have added some statements in the manuscript to make our intentions clearer to readers. Of course, the work on NEK7 PROTACs in this paper is still preliminary, and extensive medicinal chemistry experiments is warranted to optimize this compound.

Reviewer #2 (Remarks to the Author):

Review Statement:

This paper demonstrates both biological and mechanistic novelty, addressing the problem with the use of recent techniques. It is packed with a wealth of relevant information, with every sentence serving a purpose without redundancy. The experiments conducted to address the questions are precise, comprehensive, and carried

out with exceptional attention to detail. This study began with the novel observation that, despite SPOP being involved in ZMYND8 degradation, it does not alter ZMYND8 mRNA levels, protein expression, or lifetime. Building on this finding, the authors revealed that SPOP-mediated K63-linked polyubiquitination of ZMYND8 drives aberrant NEK7 transcription and mTOR inhibitor-resistant growth, identifying NEK7 as a promising therapeutic target. The successful application of a NEK7-targeting PROTAC to overcome everolimus resistance further underscores the translational potential of these findings.

Several years ago, many reports on biomolecular condensates remained focused on observational phenomena and theoretical hypotheses at the highest conceptual level. Only recently has emerging evidence started to reveal the highly complex regulatory nature of these so-called LLPS bodies, demonstrating that some condensates are functionally active while others are inhibitory. This paper goes beyond merely reporting the LLPS phenomenon of ZMYND8 and instead establishes underlying principles. The authors identify the key amino acid residue critical for ZMYND8 condensate formation, elucidating the dynamics of LLPS and verifying the downstream effects driven by its inhibitory function.

Overall, I am positive about the publication of this paper in Nature Communications. My major comments are limited to biophysical and technical aspects related to LLPS, and I would like to clarify that these suggestions are made to support and strengthen the study.

Reply: We thank the Reviewer for the positivity and support of publication of our manuscript in Nature Communications. All the suggestions and concerns of the Reviewer have been addressed fully as outlined below.

Major comments:

1. In Figures 2C, 2E, 2G, and Fig. S3B, the procedure for quantifying the number of puncta is not explained. While the “Immunofluorescent chemistry (IFC) and quantification” section in the Materials and Methods describes the experimental method, it lacks the image data quantification process. If this omission was unintentional, the authors should provide a detailed explanation. If the puncta were simply counted manually based on prominent features observed visually, I recommend using free software such as ImageJ (Fiji) to quantify blobs with an arbitrary threshold intensity above the background for more rigorous and reproducible analysis.

Reply: We thank the Reviewer for their rigorous attention to experimental and statistical methods. We are very sorry for our oversight of not including the quantification process in the Materials and Methods section of the manuscript. Specifically, to analyze the puncta numbers, cell images were analyzed using the Analyzed Particles function in ImageJ. Puncta with a size larger than 0.1 μm^2 were counted. For each biological repeat, 20 cells were re-analyzed in each group and the average number of puncta/cells was quantified. We have added the quantitative data in **Review Figure 5, 2C, 2E, 2G, and Fig. S3B**. We have also added the quantification

method to Materials and Methods.

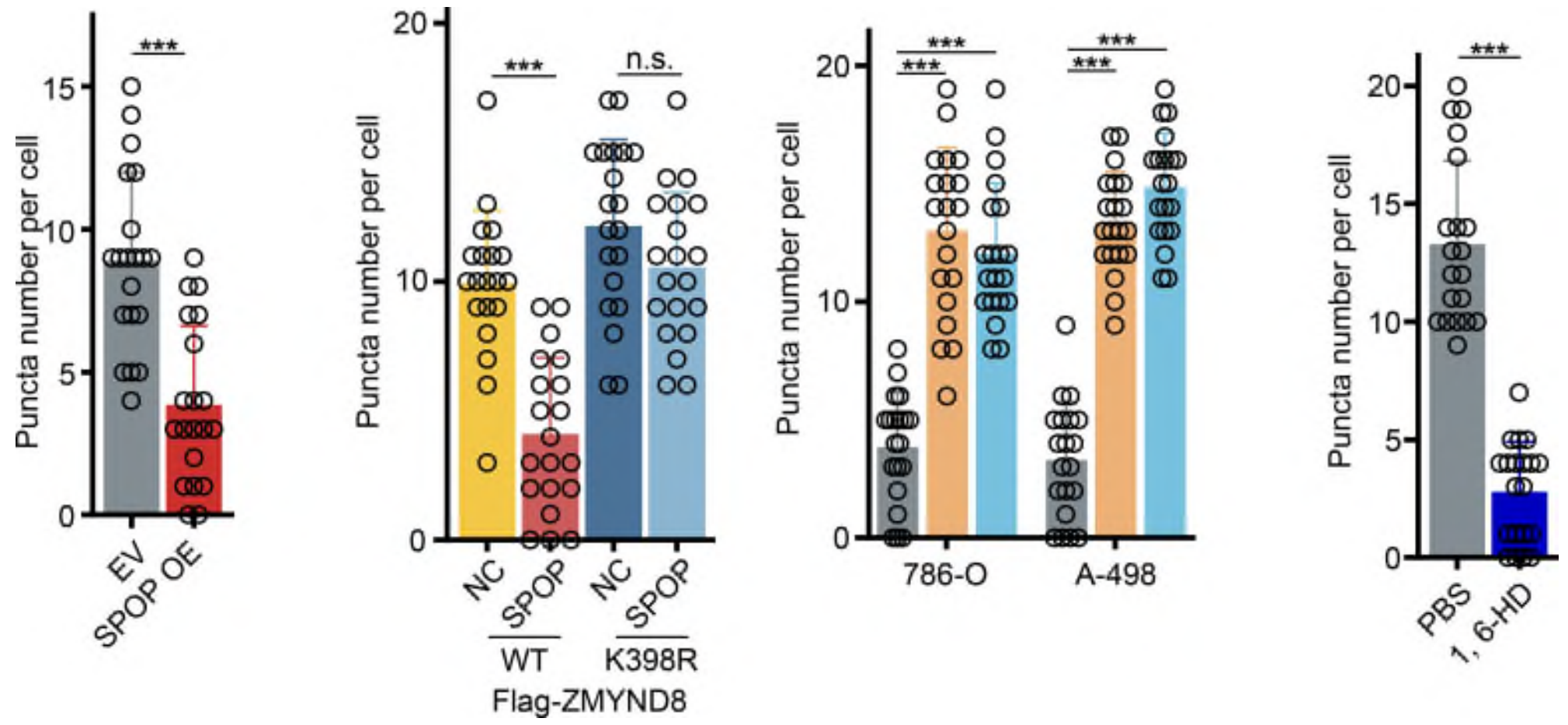

Review Figure 5. Revised Quantification of ZMYND8 puncta number in cells the corresponding experiment.

2. The paper "ZMYND8 mediated liquid condensates spatiotemporally decommission the latent super-enhancers during macrophage polarization, Pan Jia et al., Nat Comm 2021" has already established the LLPS properties of ZMYND8, so it is reasonable to assume that ZMYND8 blobs possess liquid-like characteristics. However, methods to verify LLPS in live cells, such as capturing fusion events, FRAP (fluorescence recovery after photobleaching), in vitro droplet assays, or treatment with 1,6-hexanediol, have become well-established. To confirm the LLPS nature, I suggest including experimental evidence from at least two of these methods. While it may be challenging to perform these experiments using fixed samples with IF, an alternative approach could involve using an overexpression vector for live-cell experiments. If ZMYND8 overexpression is not feasible, the authors could cite and elaborate on Pan Jia et al.'s 2021 paper to clarify the LLPS properties of ZMYND8 instead.

Reply: We thank the Reviewer for the great suggestions of determining the LLPS of ZMYND8 using additional methods. As suggested by the Reviewer, we performed fluorescence recovery experiments after photo-bleaching (FRAP) and in vitro droplet assays to determine the LLPS properties of ZMYND8. As shown in **Review Figure 6 and Fig. S3** our new data further confirmed that ZMYND8 is able to for LLPS both in vitro and in vivo. We have also cited Pan Jia et al.'s 2021 paper 34764296 4764296) in our manuscript.

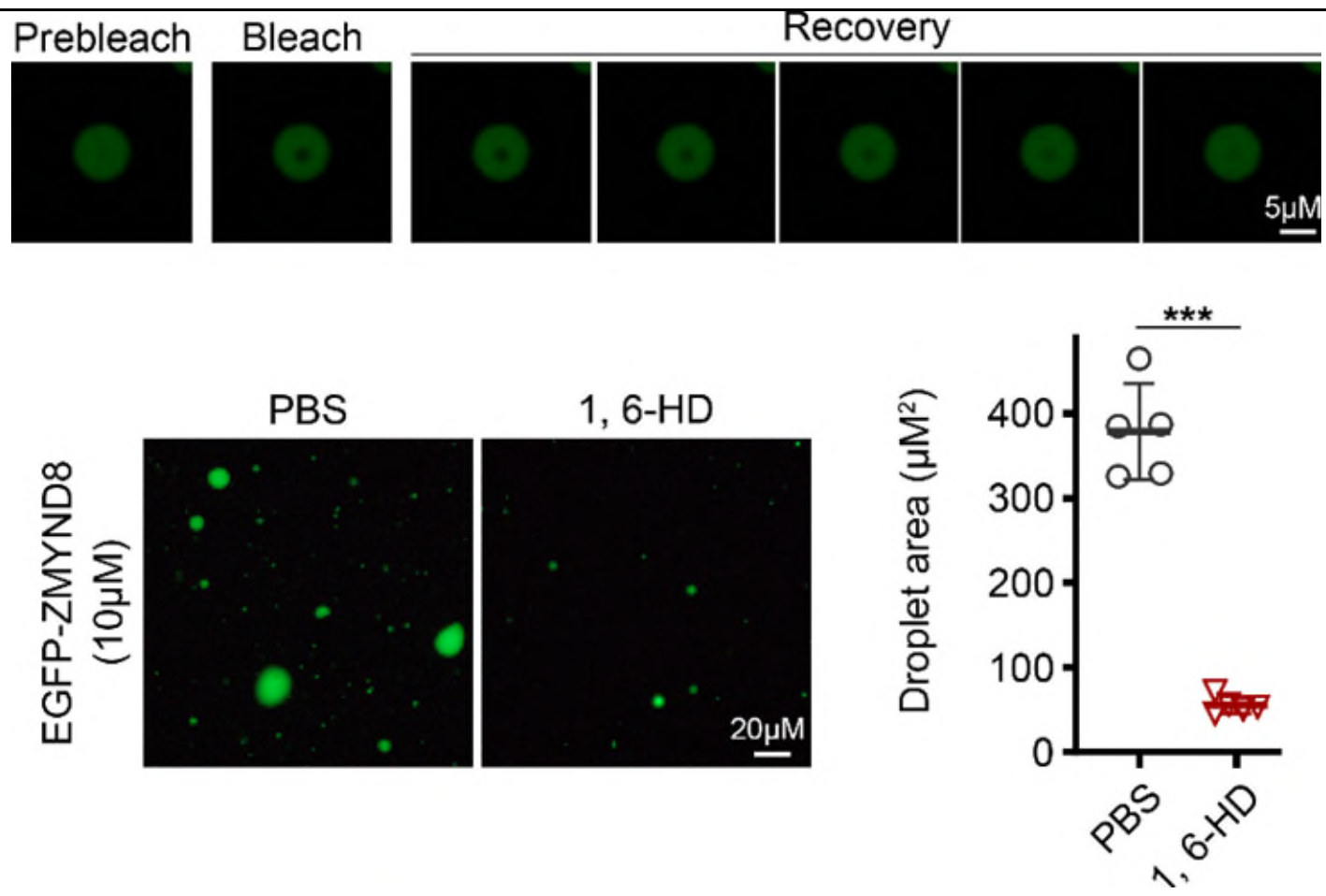

Review Figure 6. (Up) Photo-bleaching analysis of EGFP-ZMYND8 (30 µM) droplets as assessed by fluorescence recovery in a buffer containing 150 mM NaCl and 10% PEG-8000. Confocal images were taken at indicated time points relative to photo bleaching (0 s). (Down) LLPS droplets of EGFP-ZMYND8 (10 µM) are sensitive to 1,6-hexanediol (10%). Dot plots show decreases of droplet area per field in the presence of 1,6-hexanediol. Data represent mean ± s.d. (n = 5 independent experiments).

Minor comments:

1. While the results section describes the relationship between mTOR and NEK in ccRCC development and drug resistance, adding a paragraph in the introduction to explain this connection would help readers better understand the context of the study.

Reply: As suggested, we have added a paragraph in the Introduction to explain the connection of mTOR activation to drug resistance in ccRCC on page 2.

2. In the sentence, "We found that knockout of ZMYND8, a chromatin reader but not other nine top hits largely increased everolimus sensitivity in 786-O (Fig. 1E and Supplementary Fig. 1J)," it is unclear why ZMYND8, as a chromatin reader, makes cells more sensitive to everolimus. Clarification on this point would strengthen the interpretation.

Reply: We performed an unbiased screening in our study and ultimately identified ZMYND8 gene as the effector molecule of SPOP-mediated everolimus resistance. To clarify the description of our findings, we have removed "as a chromatin reader".

3. The statement "Treatment with 1,6-hexanediol, a potent inhibitor of LLPS, …" is not entirely accurate. It would be more precise to specify that 1,6-hexanediol is particularly effective against LLPS driven by hydrophobic interactions. Adding this clarification would improve the scientific accuracy of the text.

Reply: We thank the Reviewer for their meticulous suggestion. We have modified the

sentence as suggested.

Reviewer #3 (Remarks to the Author):

In this study, the authors reported that SPOP promotes K63-linked polyubiquitination of ZMYND8 for inhibiting ZMYND8 to form phase separation compartments and drives the formation of ZMYND8-ZHX2 transactivation complex, resulting in aberrant NEK7 kinase gene transcription, alternative activation of p70S6K, and mTOR inhibitor-resistant cell growth. The authors further demonstrated that inhibition of SPOP or NEK7 increased ccRCC cell sensitivity to mTOR inhibitor. Treatment with a NEK7 PROTAC inhibited aberrant p70S6K activation and overcame everolimus resistance in ccRCC cells in vitro and in vivo. This study identified a function switch of ZMYND8 driven by SPOP as a key mechanism that causes mTOR inhibitor resistance and suggested NEK7 as a potential target of increasing mTOR inhibitor sensitivity in ccRCC. Overall, this is a well-designed and thoroughly conducted study to demonstrate the crucial function of SPOP in promoting mTOR inhibitor resistance of ccRCC tumor. However, several minor concerns remain to be investigated before publication.

Reply: We thank the Reviewer for the positivity on the design and execution of our studies in this manuscript. All the minor concerns raised by the Reviewer have been addressed fully as outlined below.

1. The authors claimed that NEK7 degradation mitigated mTOR inhibitor resistance of ccRCC tumor cells, a mTOR inhibitor-resistant cell line should be used to validate this conclusion.

Reply: We sincerely appreciate the Reviewer's valuable suggestion. As suggested, we first established an everolimus-resistant subline from Caki-2 cell line (Caki-2 ER) by using the method of generating everolimus-resistant cells as reported previously (Anticancer Res 2023, PMID 37772579). WB analysis showed that NEK7 protein expression was slightly upregulated in drug-resistant cells. Importantly, addition of the NEK7 PROTAC SJW-8136 to drug-resistant cell lines Caki-2 ER restored the sensitivity of the cells to the mTOR inhibitor everolimus (**Review Figure 7 and Fig. S8**).

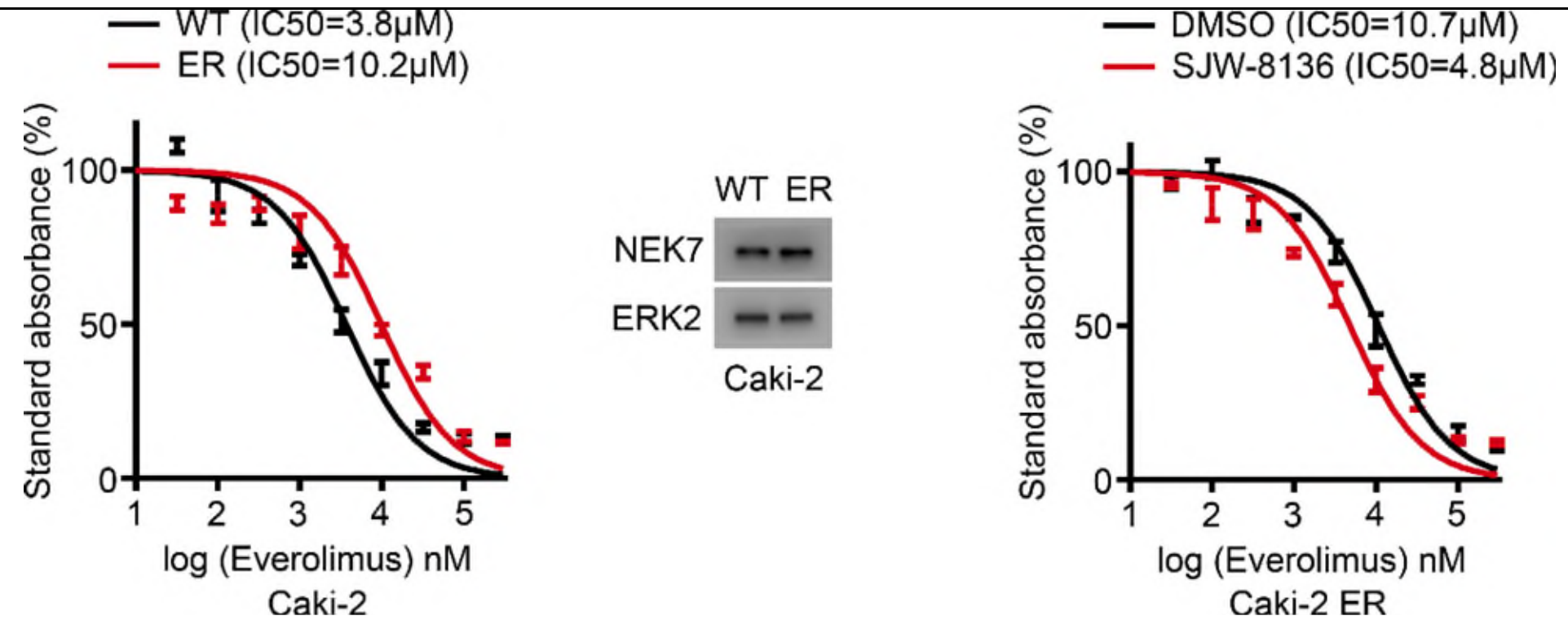

Review Figure 7. (Left) IC50 of everolimus in everolimus sensitive (WT) or resistant (ER) Caki-2 cells. Data are shown as mean ± SD (n=3). (Mid) WB analysis of indicated protein in Caki-2 everolimus sensitive (WT) or resistant (ER) cells. (Right) IC50 of everolimus in Caki-2 ER cells treated with DMSO or SJW-8136.

2. The NEK7 PROTAC was used for combination with mTOR inhibitor in vivo, the administration procedure should be provided in the method section, PK values of the NEK7 PROTAC should be evaluated.

Reply: As requested, the administration procedure of the NEK7 PROTAC has been provided in the method section on manuscript page 30. In the current study, we did not experimentally determine the PK parameters of NEK7 PROTAC in vivo. However, we performed in silico predictions using (https://lmmd.ecust.edu.cn/mmpk/) to estimate its PK properties. The predicted values (shown below) suggest that SJW-8136 exhibits moderate pharmacokinetic characteristics suitable for in vivo studies (**Review Table 1**).

Additionally, our preliminary data from in vivo assays also support the drug-like properties of this compound and demonstrate a potent antitumor activity in combination with the mTOR inhibitor. Since SJW-8136 is our lead compound, further optimization of its activity and PK profile is currently underway. We acknowledge that experimental PK studies would provide more definitive evidence for the in vivo use of this compound. Indeed, we plan to conduct these studies in the future to further validate our findings.

Review Table 1. The predicated PKs of SJW-8136 after 20 mg/kg IP

C_{max} [ng/mL]	T_{max} [h]	$t_{1/2}$ [h]	AUC [ng*h/mL]	CL/F [L/h]	F [%]
82.47	3.5	29.67	1244.64	14.8	5.55

3. The maximal degradation concentration of the NEK7 PROTACs should be quantified.

Reply: We performed a concentration gradient experiment for the PROTAC and found that the maximal degradation effect can be achieved in the range of 0.5-3 μM in 786-O

cells. Excessive drug concentration will reduce the degradation effect (**Review Figure 8 and Fig. S8C**).

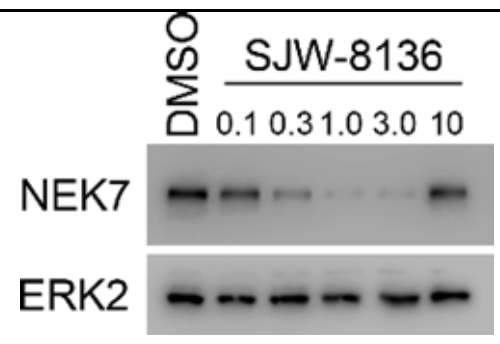

Review Figure 8. WB analysis of indicated protein in 786-O cells treated with DMSO or SJW-8136 at indicated concentrations for 24 h.

4. The advantage of the NEK7 PROTACs over the inhibitors should be discussed.

Reply: As suggested, we have discussed the advantages of NEK7 PROTACs over inhibitors and have added this content to the Discussion section of the manuscript on page 23.

5. NEK7 expression in the tumor tissue upon NEK7 PROTAC treatment should be evaluated to validate NEK7 eradication in vivo.

Reply: After removing the mouse tumor, we extracted proteins from tumor suggests and performed Western blot analysis. Our new data confirmed the eradication of NEK7 proteins by this PROTAC in vivo (**Review Figure 9 and Fig. S8Q**).

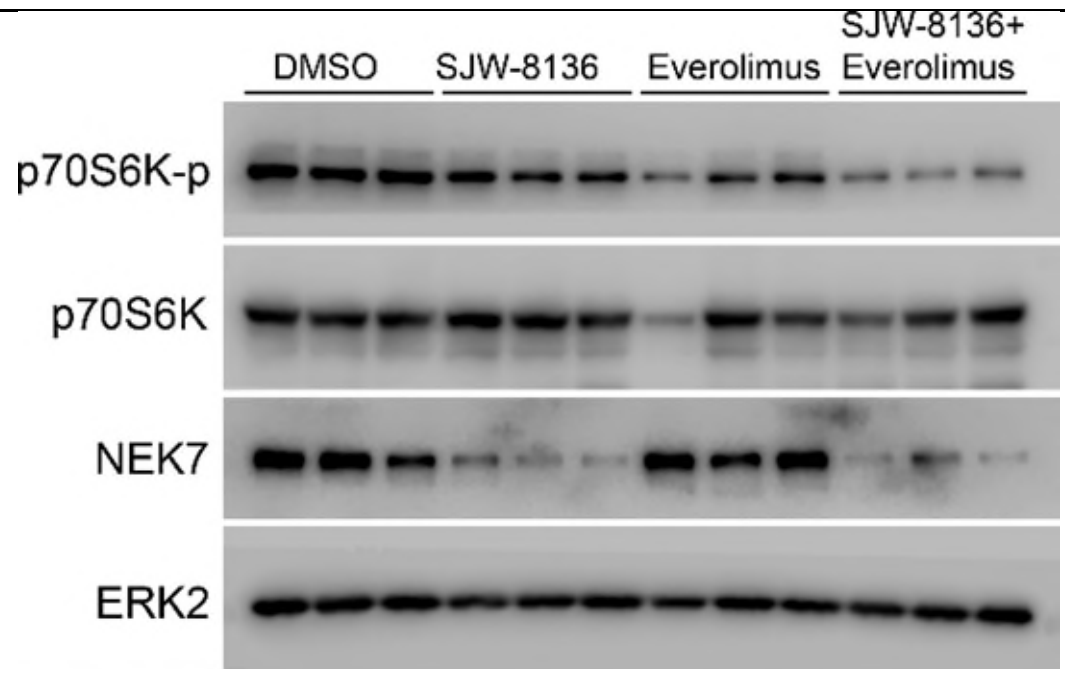

Review Figure 9. WB analysis of indicated proteins in cell lysates isolated from 786-O ccRCC tumors at the end of treatment (n=3 tumors randomly selected from each treatment group).

Reviewer #4 (Remarks to the Author):

Tang et al. show that SPOP promotes non-degradable K63-linked ubiquitination of ZMYND8 at lysine 398 in human ccRCC cell lines. The ubiquitinated ZMYND8 inhibits the formation of phase separation compartments, but promotes the transcription of NEK7 by forming a transactivation complex with ZHX, leading to activation of p70S6K and growth resistance to the mTOR inhibitor everolimus. Inhibition of either SPOP or NEK7 enhances the growth inhibitory effect of everolimus. Also, both in vitro

and in vivo studies show that inhibition of NEK7 can suppress aberrant p70S6K activation and overcome everolimus resistance. The research reveals that the SPOP-ZMYND8-NEK7-p70S6K axis plays a critical role in ccRCC resistance to mTOR inhibitors. The finding is novel and interesting. However, before the manuscript is suggested for publication in the journal, some concerns need to be addressed.

Reply: We thank the Reviewer for recognizing our finding novel and interesting. All the concerns raised by the Reviewer have been addressed fully as outlined below.

Major concerns:

1) Full activation of p70S6K is determined by its phosphorylation on multiple sites within the catalytic, linker, and pseudosubstrate domains. While the phosphorylation of Thr229 (primarily by PDK1) in the catalytic domain and Thr389 (primarily by mTOR) in the linker domain is most crucial for the kinase activity, the phosphorylation on Ser411, Thr421, and Ser424 in the pseudosubstrate domain is also important for the activation of p70S6K. Fig. 3 and Fig. S4 show that SPOP-mediated ZMYND8 ubiquitination promotes ccRCC cell proliferation and Thr389 phosphorylation of p70S6K independently of mTOR. Given the availability of antibodies to phospho-p70S6K on Thr412 and Thr229, it is good to determine whether the phosphorylation of p70S6K on Thr412 and Thr229 is altered. The result may advance our understanding of why SPOP-promoted cell proliferation is mTOR-independent.

Reply: We thank the Reviewer for the excellent point. After acquiring an antibody targeting p70S6K Thr229, we first experimentally validated the effects of SPOP, ZMYND8-WT, and ZMYND8-K398R on p70S6K Thr389 and Thr229. Similar to our previous results, we found that the SPOP-ZMYND8 axis specifically affected p70S6K Thr389 phosphorylation activity but had little effect on Thr229 (**Review Figure 10 and Fig. S4G and H**).

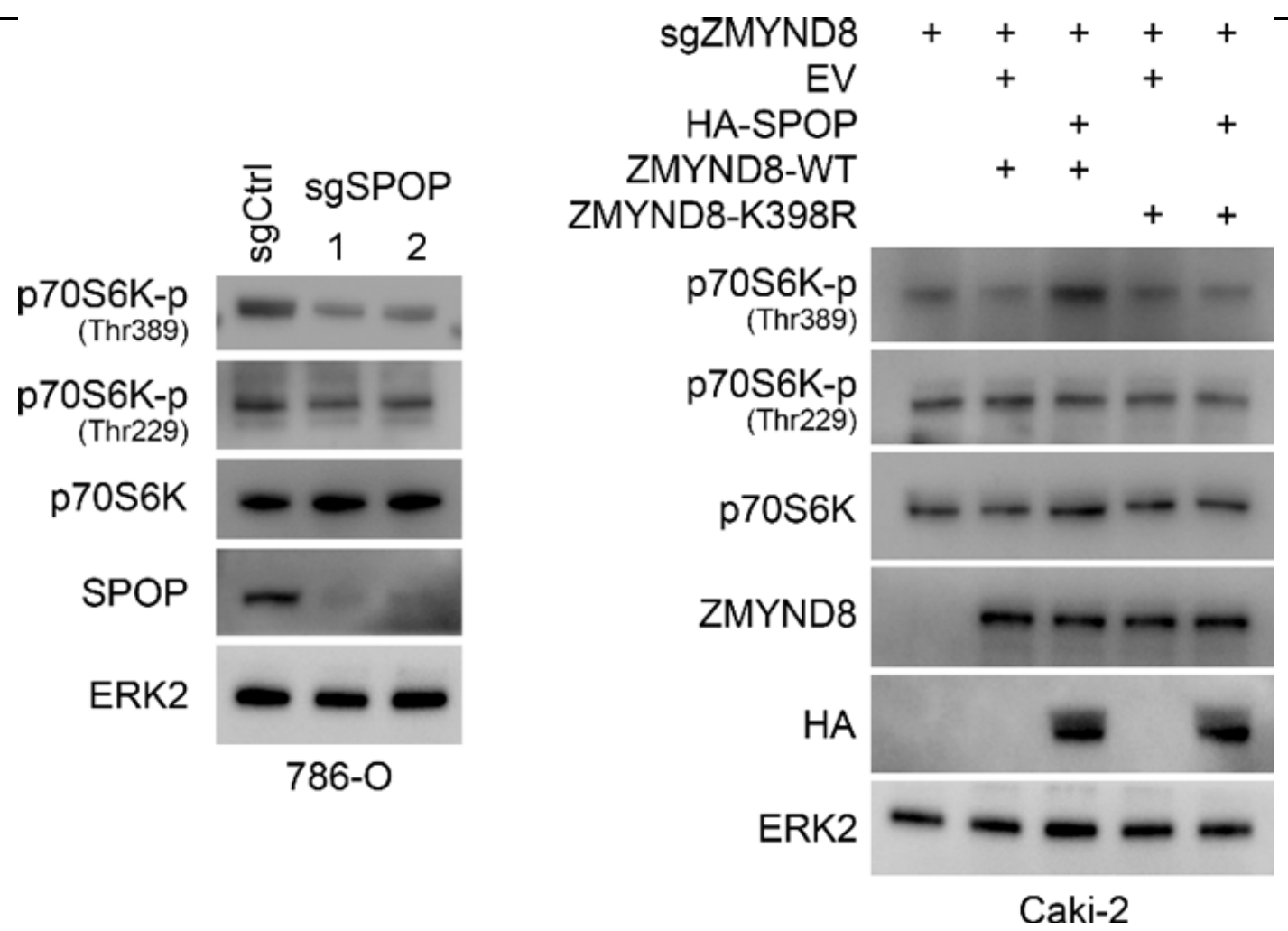

Review Figure 10. WB analysis of indicated proteins in 786-O and Caki-2 transfected with indicated plasmids.

2) It has been shown that NEK6 and NEK7 can phosphorylate p70S6K on Thr412 (PMID: 11516946; PMID: 12589797). Fig. 4 and Fig. S5 show that the SPOP-mediated polyubiquitination of ZMYND8 upregulates the transcription of NEK7, consequently increasing Thr389 phosphorylation of p70S6K. It is unclear whether NEK7 impacts the phosphorylation of p70S6K on Thr412 and Thr229 in ccRCC cells. This piece of data may help understand why inhibition of NEK7 can enhance the anticancer activity of everolimus (mTOR inhibitor).

Reply: We found that NEK7 also only affects the phosphorylation of p70S6K Thr389, but has little effect on Thr229 (**Review Figure 11 and Fig. S5H**).

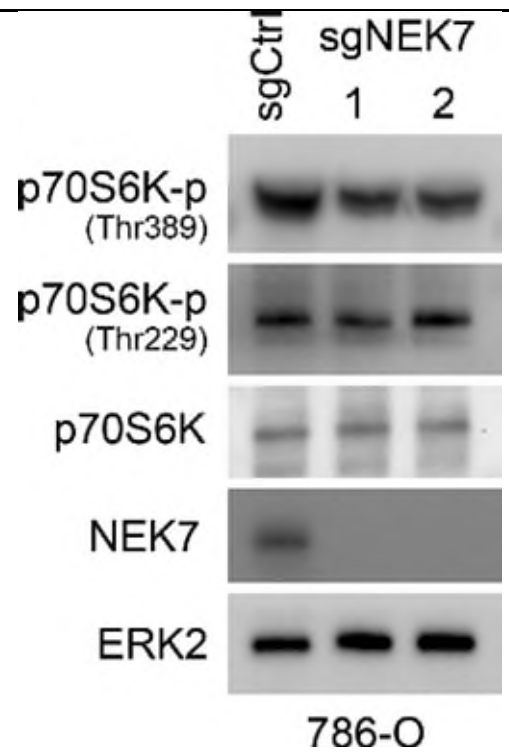

Review Figure 11. WB analysis of indicated proteins in 786-O and Caki-2 transfected with indicated plasmids.

3) It has been shown that treatment with everolimus at low micromolar concentrations (~1 μM) can potently inhibit the phosphorylation of p70S6K in 786-O cells (PMID: 23571736; PMID: 30771617). However, Fig. 6A and Fig S7A show that treatment with everolimus at a higher concentration (5 μM) only moderately inhibits the phosphorylation of p70S6K (Thr389) in the cells. Please have a discussion on the discrepancy.

Reply: In fact, the experimental conclusions in the two articles you mentioned are inconsistent. In the first article (PMID: 23571736), after 24 hours of treatment with 1 μM everolimus in 786-O cells, a considerable amount of p-p70s6k still remained. In the second article (PMID: 30771617), after just 6 hours of treatment with 0.1 μM everolimus in the same cell line (786-O), p-p70s6k was almost undetectable. While we cannot know the specific conditions and details of the experimenters from these two groups, we can assure here that our conclusions were derived from repeated experiments with reproducible results.

4) Fig. 6J shows that treatment with SJW-8136 (NEK 7 degrader) moderately reduces the level of NEK7 in 786-O cells, compared to treatment with sgNEK7 (Fig. 6A). However, SJW-8136 and sgNEK7 can enhance the anticancer activity of everolimus at a similar level, comparing Fig. 6B, C, E, F and G to Fig. M, N, O, P and Q. The results

suggest that SJW-8136 be not a selective degrader of NEK7. In addition to inhibiting the phosphorylation of Stat3 (Tyr705), SJW-8136 may target more proteins critical for cell proliferation and tumor growth. If there were more convincing data available, it would be better to soften the tone, regarding the specific effect of SJW-8136 on NEK7 degradation.

Reply: We thank the Reviewer for noticing an experimental detail we have missed. Since we repeatedly confirmed in subsequent experiments that SJW-8136 significantly degrades NEK7 at a concentration of 0.5 μM, we have repeated this experiment and replaced the previous image (**Review Figure 12 and Fig. 6J**). The previous results may have been due to issues with drug preparation or storage.

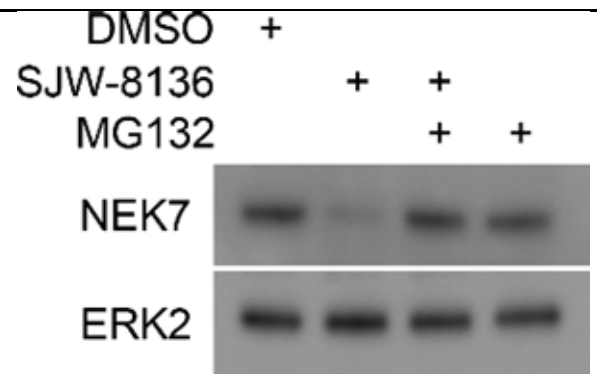

Review Figure 12. WB analysis of NEK7 protein in 786-O cells treated with SJW-8136 (0.5 μM, 24h) and/or MG132 (20 μM, 12 h).

5) It is suggested to have semi-quantification analyses for all blots, which can be shown as “Supplemental data”.

Reply: We used Image J software to semi-quantify the grayscale of all blots. Considering that there are many blot experiments in this study, we did not put the protein quantification data into figures, but put it in the Source Data.

Minor Concerns:

1) Please define all abbreviations at the first appearance, such as BRD4, SPOP, AR, ERG, ZHX2, p70S6K, etc.

Reply: We carefully reviewed the manuscript and defined all the abbreviations of proteins or genes when they first appeared.

2) Please provide more detailed information about how each experiment was performed in the figure legends. For example, how long time were the cells treated with compounds for the MTS assay, colony formation assay, and Western blotting?

Reply: We thank the Reviewer for pointing these out. We have carefully reviewed the figures, and some necessary experimental details have been added to the figure legends.

3) Please carefully check whether the descriptions in the figure legends are correct. For instance, “WB analysis of SPOP protein level in 786-O cells transfected with indicated plasmids” (Fig. S1B) vs. “786-O cells infected with lentivirus for sgControl

or sgSPOP…" (Fig. 1A).

Reply: We carefully reviewed the figures and figure legends and legend errors have been corrected.

4) It is good to provide uncropped western blots (raw data) for related figures, which can be shown as "Source data".

Reply: Uncropped western blots and other necessary raw data have been organized into Source Data Excel files.

Reviewer #5 (Remarks to the Author):

Cullin-RING ligases (CRL) play broad and essential roles in gene expression and tissue homeostasis. Not surprisingly, CRL dysfunction can lead to a wide range of disease pathologies including cancer. In particular, the CRL CUL3SPOP has been shown to function as a tumor suppressor in many solid tumor types, including cancer of the prostate, endometrium, and breast (Yang and Zhu, 2022). On the other hand, SPOP is highly expressed in clear cell renal cell carcinomas (ccRCC), the most prevalent type of kidney cancer, where it is believed to function as an oncogene (Zhang et al., 2023). Thus, determining the molecular mechanisms by CUL3SPOP regulates tumor formation is important for developing new cancer treatments as well as overcoming resistance to existing chemotherapeutics.

Tang et al. seek to elucidate the role of the CRL, CUL3SPOP, in driving ccRCC and promoting its resistance to current treatments, namely mTOR inhibitors everolimus and temsirolimus. The authors show that SPOP ubiquitinates ZMYND8 within its intrinsically disordered region at lysine 398, inhibiting phase separation of ZMYND8. Ubiquitinated ZMYND8 forms a complex with ZHX2 to induce the expression of NEK7, which phosphorylates p70S6K in an mTOR-independent manner. This ultimately leads to cancer cell proliferation and explains why mTOR inhibition would have no effect. The authors have also made the novel finding that the small molecule SJW8136 is a NEK7 inhibitor and have shown that SJW8136 reduces the phosphorylation levels of p70S6K and enhances the sensitivity of cancer cells towards everolimus.

The manuscript can be accepted for publication after the following points have been addressed:

Reply: We thank the Reviewer for the positivity on our manuscript. All the concerns raised by the Reviewer have been addressed fully as outlined below.

Major issues:

1. Several areas of the manuscript need to be revised to prevent over interpretation of the results:

a. The last sentence of the introduction, “This ubiquitination event acts as a molecular switch...mTOR inhibitor resistance in ccRCC.” is a very direct statement and can be revised to be more descriptive instead.

b. In the third section of Results (SPOP-mediated ZMYND8 ubiquitination..), first paragraph, last sentence - “…suggesting a functional switch of ZMYND8..” seems like an oversimplification of the authors’ findings.

c. Same section as above, last paragraph, last sentence, “These data suggest that SPOP-mediated ubiquitination of ZMYND8 promotes p70S6K phosphorylation…”. Perhaps this could be adjusted to “ZMYND8 is needed/required for p70S6K phosphorylation”?

d. Discussion section, first paragraph, last sentence - “assembly of the ZMYND8-ZHX2 transactivation complex that promotes expression of oncogenes...”. The authors have only found the altered expression of NEK7, hence, this sentence seems to be an overstatement.

Reply: Thanks to the reviewer for all the great suggestions. The above points have been well taken and we have modified the manuscript accordingly.

2. In Fig. 1, the Flag-ZMYND8 immunoprecipitations performed with HA-Ub are done under native conditions. Thus, the detected polyubiquitin signal could arise from SPOP substrates that may be associated with ZMYND8 rather than ZMYND8 itself. To distinguish this, can the authors either: (1) perform a denaturing His-Ub pulldown and blot for Flag-ZMYND8 or (2) look for the presence of a high-molecular weight smear using anti-FLAG antibody in Fig. 1I or 1J?

Reply: As suggested by the Reviewer, we performed a denaturing His-Ub pulldown assay and blot for Flag-ZMYND8. We demonstrated that the ubiquitination modification was originated from ZMYND8 protein (**Review Figure 13 and Fig. S2D**).

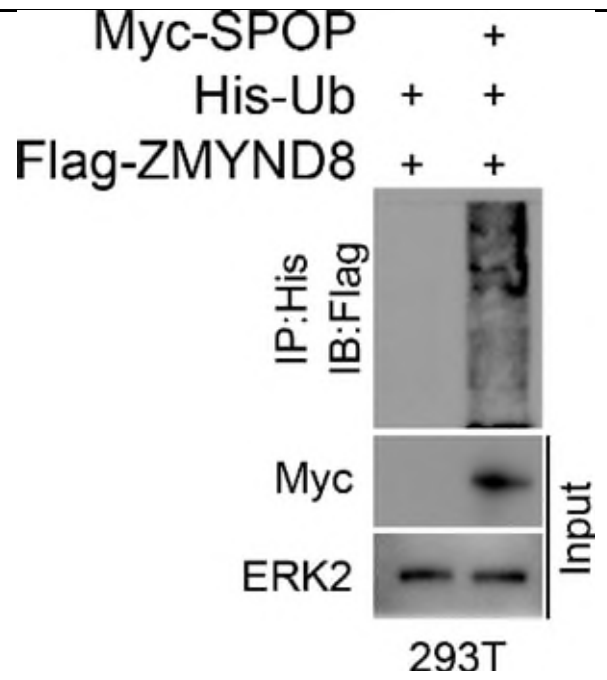

Review Figure 13. WB analysis of WCL and co-IP samples in 293T cells transfected with indicated plasmids. ERK2 was used as a loading control.

3. For Figure 3H, can the authors include a positive control for mTOR inhibition to ensure that repression of this pathway in 786-O cells indeed leads to loss of p70S6K

phosphorylation?

Reply: As suggested, we included a positive control group by treating 786-O cells with everolimus for 24 hours. We demonstrated through WB analysis that the phosphorylation activity of the mTOR-p70s6k signaling pathway in the cells decreased to a certain extent (**Review Figure 14 and Fig. S4F**).

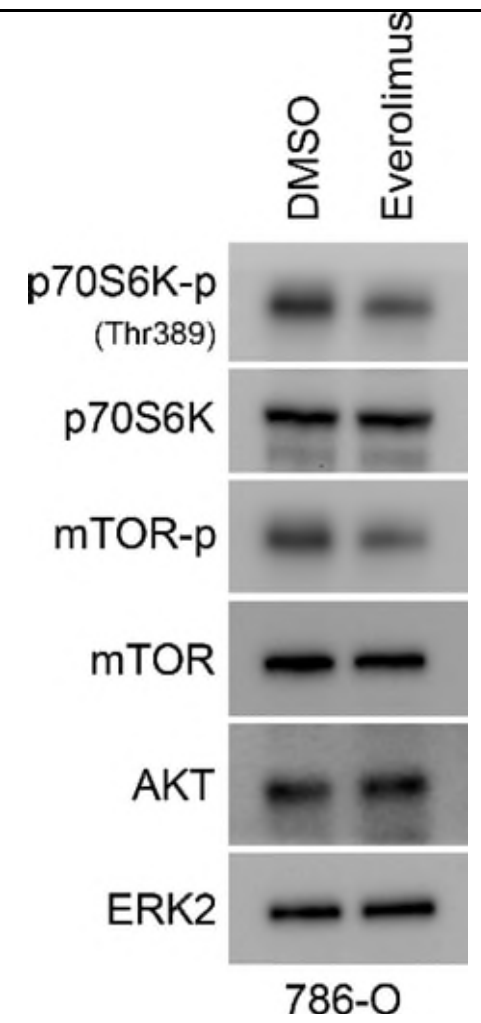

Review Figure 14. WB analysis of indicated proteins in 786-O cells treated with DMSO or everolimus (5μM) for 24 hours.

4. Comparing Figures 1B and S1G, it seems that SPOP inhibitor is causing more cell death as compared to knockdown of SPOP. Please include appropriate controls (i.e. treatment of CAKI2 cells with SPOP inhibitor) to account for any pleiotropic effects.

Reply: The experimental conditions for SPOP inhibition and gene knockdown were somewhat different. For example, the control group may have used DMSO, been transfected with an empty plasmid, or used a transfection reagent, which could have affected cell proliferation. Therefore, we believe that comparing the results of these two different experiments can be very challenging. The development of the SPOP inhibitor has been reported previously in detail (Cancer Cell 2016, PMID 27622336), which is not a key focus of our current study.

5. In vitro reconstitution assays are needed to support the claim that ZMYND8 is a substrate of SPOP. At a minimum, the authors need to show direct binding between purified recombinant ZMYND8 and SPOP. However, an in vitro ubiquitination reaction would work best to address this point.

Reply: We agreed with the Reviewer. Indeed, the same concern was raised in the first comment of Reviewer #1. As can be seen in our response discussed above, to determine whether the polyubiquitination of ZMYND8 is indeed directly modified by SPOP, we performed in vitro polyubiquitination assay using purified proteins and the method as

we reported previously (Mol Cell 2015, PMID 26344096). Our new data showed that only in the presence of SPOP, CUL3-RBX1 protein complex was able to promote polyubiquitination of purified ZMYND8 in vitro (**Review Figure 2 and Figure S2**).

6. In Figure S2A, failure to detect interaction between MYC-SPOPΔMATH and Flag-ZMYND8 could arise due to gross misfolding of the deletion mutant. Can the authors either provide a reference that supports the structural integrity of the ΔMATH mutant, or, perform the same assay via point mutation of the MATH domain?

Reply: F102C and F133V are two of the most common SPOP point mutations that are single amino acid mutants with a mutation in the MATH domain disrupting the ability of SPOP to bind its substrates (Cancer Research 2021, PMID 33762355). We employed these two mutants to validate its interaction of SPOP with ZMYND8. We found that the interaction between SPOP mutants and ZMYND8 was barely detectable (**Review Figure 15 and Fig. S2C**). It is important to note that our current study is mainly focusing on renal cancer but not on prostate or endometrial cancer. SPOP is often overexpressed and no point mutation has been reported in renal cancer. That's the reason we did not originally use the point mutants of SPOP to perform the experiments.

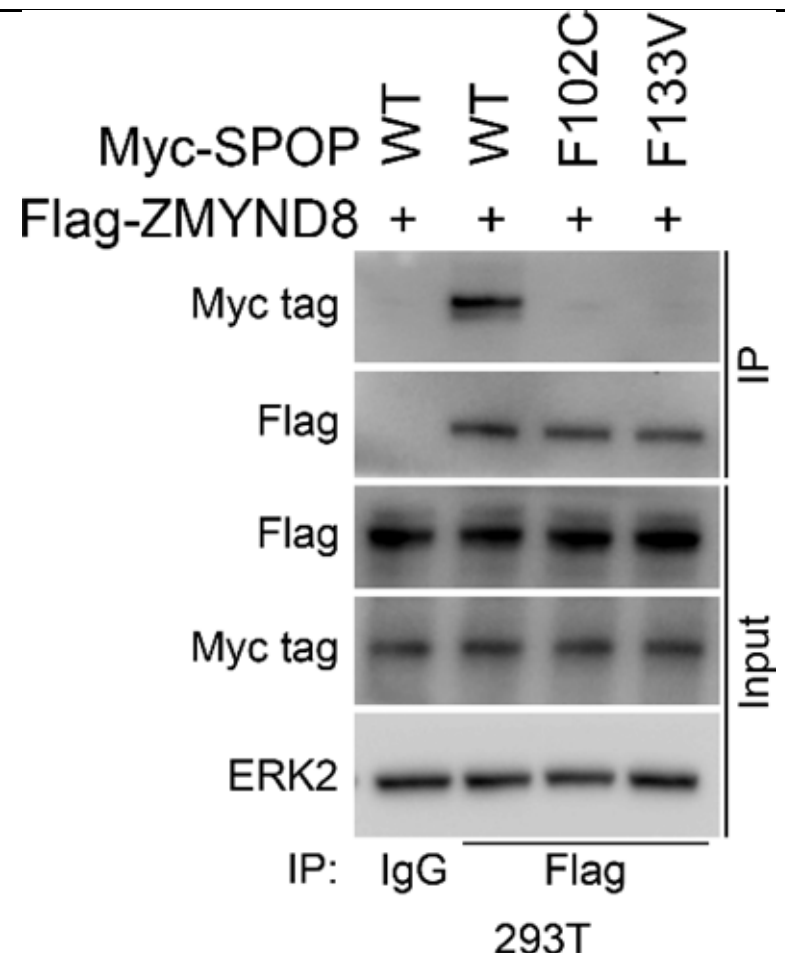

Review Figure 15. WB of co-IP samples of IgG or Flag antibody in whole cell lysate (WCL) of 293T transfected with indicated plasmids.

7. The potential reason for C-terminal 1088-GSSSS-1092 motif of ZMYND8 being critical for SPOP binding should be included in the discussion. Can this motif be targeted for therapeutic purposes?

Reply: This is a great idea for follow-up research on this topic. However, we must admit that the development of small molecule drugs targeting specific peptides is not the main research direction of our research group. Of course, we can try to cooperate with relevant drug development research groups on this issue in the future. Nevertheless, we have added a discussion in the revised manuscript on page 22.

8. In Figures 2D and E, the image shown does not appear to match the quantification.

Reply: Thanks for pointing this out. To address this point, we re-quantified the results. For each biological repeat in Figures 2D and E, 20 cells were re-analyzed in each group and the average number of puncta/cells was quantified. For analyzing puncta numbers, cell images were analyzed by the Analyzed Particles function in ImageJ. Puncta with a size larger than 0.1 μm^2 were counted (**Review Figure 16 and Fig. 2D and E**). We have added the quantification method to Materials and Methods.

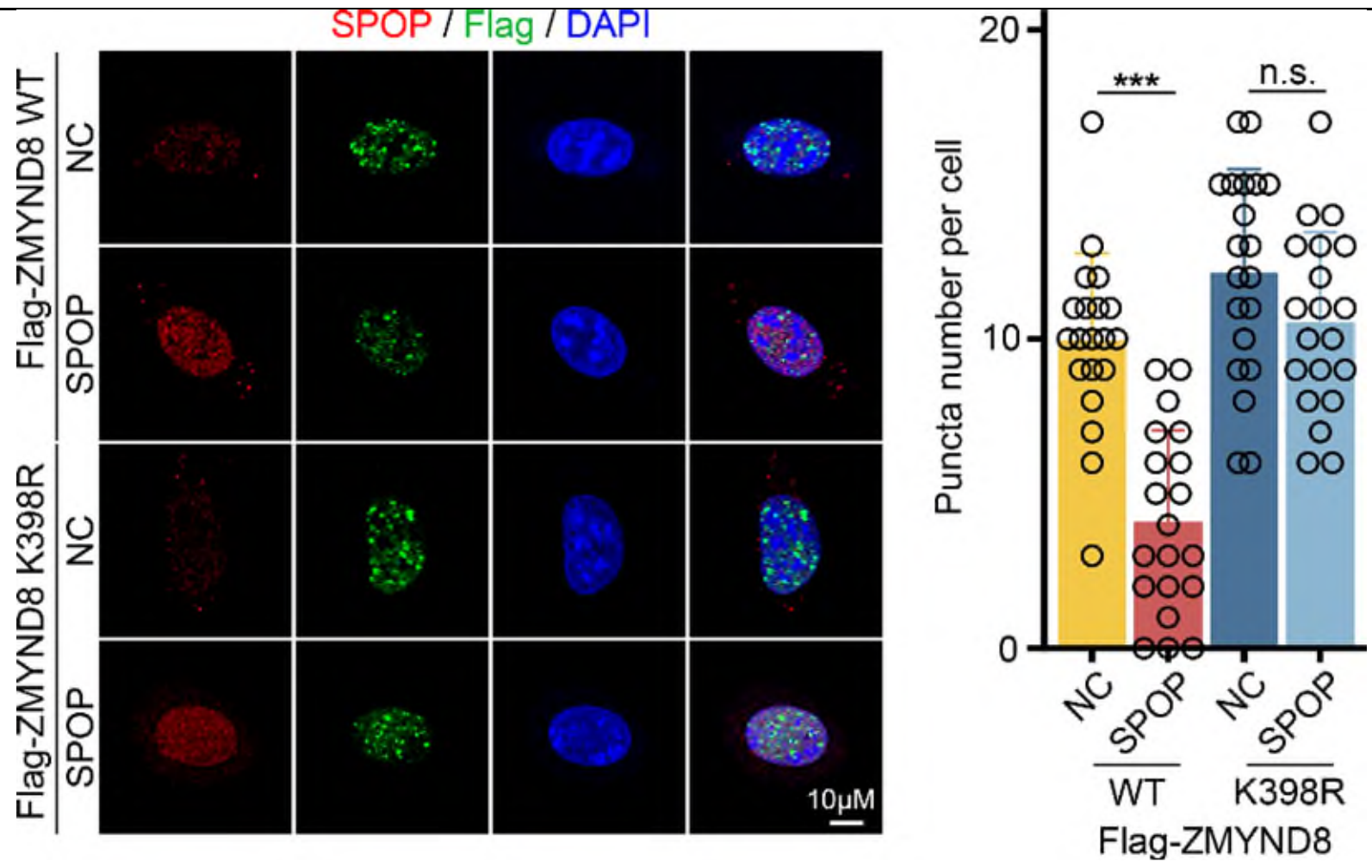

Review Figure 16. (Left)Immunofluorescent cytochemistry (IFC) of SPOP and Flag in 293T cells transfected with indicated plasmids. (Right) Quantification of Flag puncta number in cells from left figure. ***P < 0.001, n.s., not significant.

9. Addition of 1,6-hexanediol seems to cause significant changes in nuclear morphology (Fig. 2H). A negative control (sgCtrl) cell line treated with 1,6-HD should be included to ensure these effects are not specific to sgSPOP cells. A discussion should be included if there are any known intranuclear effects of 1,6-HD.

Reply: We believe this phenomenon is simply an accidental artifact of our image selection process. We re-examined our experimental images and found that some anomalous nuclei were present in each experimental group, and this was not due to 1,6-HD treatment. As shown in the figure below (**Review Figure 17**), Caki-2 cell nuclear morphology was not affected by 1,6-HD.

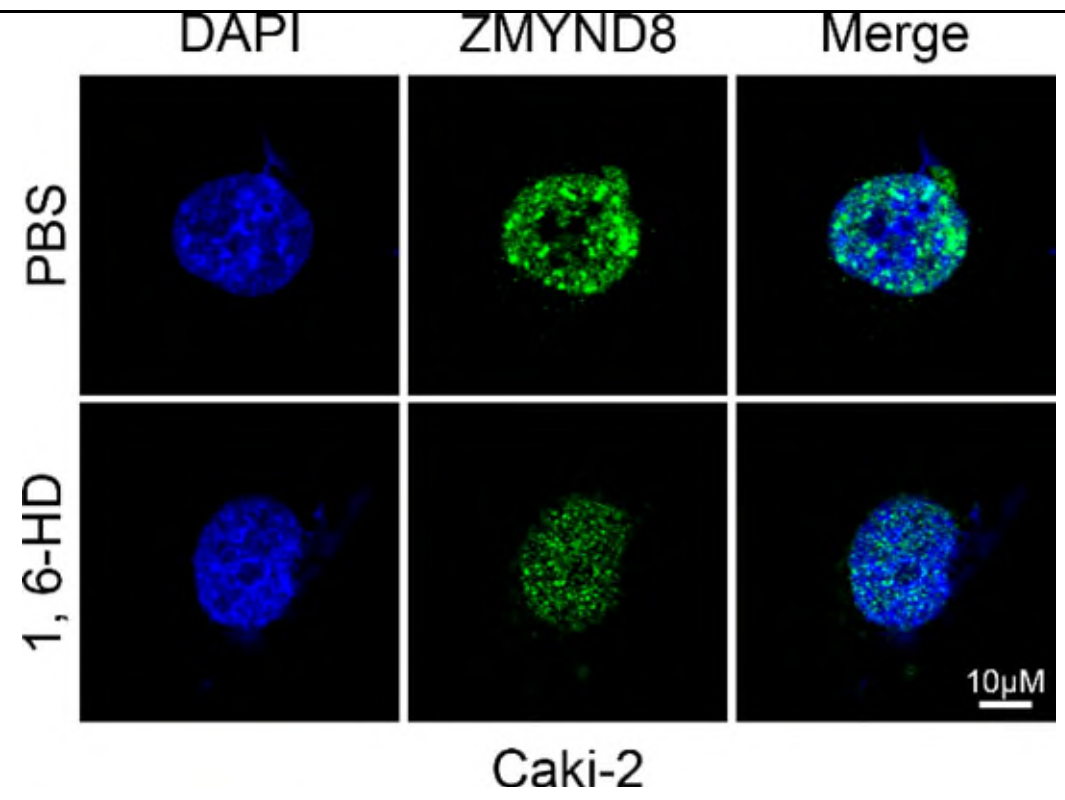

Review Figure 17. Immunofluorescent cytochemistry (IFC) of Caki-2 cells treated with DMSO or 1,6-hexanediol.

10. Figure 4 – Can the authors assess the level of phosphorylated p70S6k in NEK7 and ZMYND8 double knockouts, to confirm that their relationship is epistatic? If the double knockout reveals a synergistic effect, this would not necessarily invalidate the authors' model, but it would be important to know the relative magnitude of each gene's contribution.

Reply: We performed a double knockdown of NEK7 and ZMYND8 in 786-O cells and found that simultaneous knockdown of both proteins did not further reduce p70S6k phosphorylation (**Review Figure 18 and Fig. S5J)**, further supporting the notion that NEK7 and ZMYND8 are involved in the same signaling pathway.

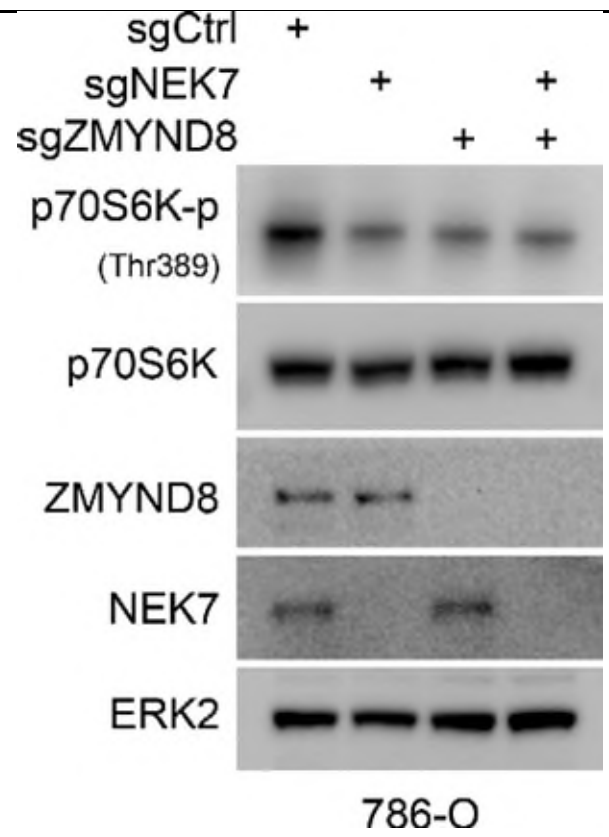

Review Figure 18. WB analysis of indicated proteins in 786-O cells transfected with indicated plasmids.

11. In addition to Figures S5 C, D it would be useful to know how levels of phosphorylated p70S6K compare in ccRCC versus normal tissue (if such data are available). I believe this would strengthen the claim of the authors of using SJW-8136 as a potential therapeutic molecule.

Reply: It is generally accepted that the activation of the mTOR-P70S6K signaling pathway and the high expression of p-p70s6k occur in renal cancer. For example, it has

been reported previously that the level of p-p70s6k in renal cancer is higher than that in surrounding normal tissues (Ann Clin Lab Sci 2006, PMID 16951269; Br J Cancer 2024, PMID 38341510). These two publications have been cited in the revised manuscript.

12. Are there any known roles of SPOP in mediating nondegradative ubiquitination of proteins other than ZMYND8 and, if so, can the authors comment on how their observations compare with these other findings?

Reply: A few proteins have been identified as non-degradation ubiquitination substrates of SPOP, including 53BP1 previously reported by our group (Sci Adv 2021, PMID 34144977), and SQSTM1/p62 (Cell Death Differ 2022, PMID 34987184). The common point between our findings and these findings is that these nondegradative ubiquitination modifications often result in changing the function of the substrate proteins, rather than the common ubiquitination degradation of the protein.

Minor issues:

1. Cited references are not matching with the text in certain places. For example, in the second paragraph of the Introduction ("…serves as a reader of the dual histone mark H3K4me1/H3K14ac4."), this reference should be "Li N et al., Mol Cell, 2016".

Reply: We have carefully examined our citation of references to make sure our reference citations are accurate.

2. The authors have used multiple cell lines throughout their work. To help the reader and avoid confusion, all figures should be labeled with its respective cell line (not just in the figure legend). For example, none of the blots in Fig. 1 using 293T cells are labeled as such.

Reply: We have indicated the cell line names used in all figure panels and legends.

3. In Fig. 1C and D, a Western blot showing SPOP overexpression for these experiments is missing.

Reply: SPOP overexpression WB has been performed and new data have been included in the revised manuscript (**Review Figure 19 and Fig. S1E**).

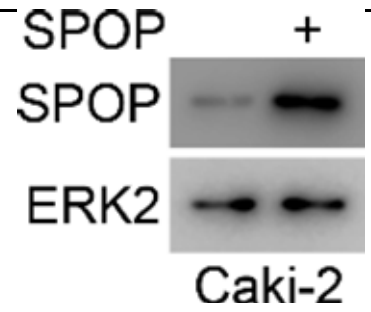

Review Figure 19. WB of indicated proteins in Caki-2 cells transfected with indicated plasmids.

4. Please include the molecular weight standard for western blot panels. This is particularly important for polyubiquitin signals that should be larger in size compared to FLAG-ZMYND8 (Fig. 1I, 1J, 1L, 1M, 1P, S2H, S8E).

Reply: We have included the molecular weight of the ubiquitinated bands in the figures. For other bands, due to limited image space, we have annotated the molecular weight of other bands in the Source Data.

5. Figure labeling needs to be checked. For example, Fig. S7E should be labeled "NEK7" instead of "SPOP".

Reply: We thank the Reviewer for pointing out this error. We carefully reviewed the figures and legends, and such errors have been corrected.

6. Methods section does not include information on protocol followed for quantification of ZMYND8 puncta. Are puncta defined based on size or fluorescence intensity?

Reply: We are sorry for our oversight for not including the quantification method in the Materials and Methods section of the manuscript. We have added the detailed quantification method to Materials and Methods on page 30.

Tang et al. seek to uncover the molecular basis by which CUL3SPOP promotes prostate cancer and its resistance to mTOR inhibitors, a class of small molecule that is used as a second-line treatment for this cancer type. The authors' data are generally of high quality and their claims supported by experimental evidence. One of the authors' major findings, that ZMYND8 may be a novel substrate of CUL3SPOP, needs to be substantiated with additional experiments to rule out the possibility that its ubiquitylation might occur indirectly (i.e. possible contribution of other E3, coadaptors, or substrates). If this cannot be reconciled, then the text will need to be adjusted accordingly to allow consideration for alternative explanations. Overall, this manuscript would be of significant interest to the scientific community and I would support publication provided the above corrections can be incorporated.

Reply: We thank the Reviewer for recognizing that our data are generally of high quality and our claims supported by experimental evidence. We also thank the Reviewer for recognizing that this manuscript would be of significant interest to the scientific community and therefore would support publication provided the above corrections can be incorporated.

As to the concern regarding whether ZMYND8 is a novel substrate of CUL3-SPOP, we have added in vitro polyubiquitination assays by using reconstituted CUL3-SPOP ubiquitination system and purified ZMYND8 as a substrate. As discussed in our response to the first comment of Reviewer #1 (Review Figure 2), our new data showed

that only in the presence of SPOP, CUL3-RBX1 protein complex was able to promote polyubiquitination of purified ZMYND8 in vitro (**Review Figure 2 and Figure S2**), supporting our conclusion that ZMYND8 is a direct ubiquitination target of SPOP.

Authors' Response to Reviewers' Comments on NCOMMS-24-78902A

We very much thank the Editor for publishing the manuscript in principle. We revised out paper according to the remaining comments from reviewers.

REVIEWER COMMENTS

Reviewer #1 (Remarks to the Author):

The authors have done extensive revisions to address the prior comments and I would recommend the publication of the paper.

Reply: We thank the Reviewer for the positivity and support of publication of our manuscript in Nature Communications.

Reviewer #2 (Remarks to the Author):

The authors have fully addressed the concerns I previously raised. They now provide rigorous evidence and clear explanations regarding the liquid properties of ZMYND8 under various conditions, leaving no remaining ambiguities. The revision has markedly improved the overall clarity of the article. I recommend publication of this manuscript in Nature Communications.

Reply: We thank the Reviewer for the positivity and support of publication of our manuscript in Nature Communications.

Reviewer #3 (Remarks to the Author):

The authors had sufficiently and correctly responded to all my comments, I prefer to recommend acceptance for publication at current stage.

I checked the procedure for PROTAC synthesis in the supporting information file, all the synthesis protocol and the reaction condition are reasonable. Therefore I recommend acceptance for publication.

Reply: We thank the Reviewer for the positivity and support of publication of our manuscript in Nature Communications.

Reviewer #4 (Remarks to the Author):

The manuscript has been well revised.

Reply: We thank the Reviewer for the positivity and support of publication of our manuscript in Nature Communications.

Reviewer #5 (Remarks to the Author):

Cullin-RING ligases (CRLs) are critical regulators of gene expression and tissue homeostasis, and their dysfunction is implicated in diverse disease pathologies, including cancer. Among them, the CUL3-SPOP complex has been shown to act as a tumor suppressor in prostate, endometrial, and breast cancers, but intriguingly functions as an oncogene in clear cell renal cell carcinoma (ccRCC). In this study, Tang et al. uncover a novel mechanism by which CUL3-SPOP drives ccRCC progression and resistance to mTOR inhibitors, demonstrating that SPOP ubiquitinates ZMYND8 at lysine 398, thereby disrupting its phase separation and enabling complex formation with ZHX2 to induce NEK7 expression. This leads to mTOR-independent phosphorylation of p70S6K, providing a compelling explanation for the failure of mTOR-targeted therapies, while the identification of the NEK7 inhibitor SJW8136 highlights a potential therapeutic strategy to restore sensitivity to everolimus. Overall, this work is of high quality, its claims well supported by the data, and the findings will be of broad interest to the cancer biology community.

The authors have satisfactorily responded to all of our queries and concerns. One error that should be addressed is in Figures 2D and E where in the text the authors mention using Caki-2 ZMYND8-KO cells. However, the figure is labeled as 293T cells. A thorough proofreading will be critical to avoid these errors. We recommend this manuscript to be accepted for publication following this minor revision.

Reply: We thank the Reviewer for pointing out this error. We carefully reviewed the figures and legends, and we have been corrected the cell line name in Figures 2D and E.

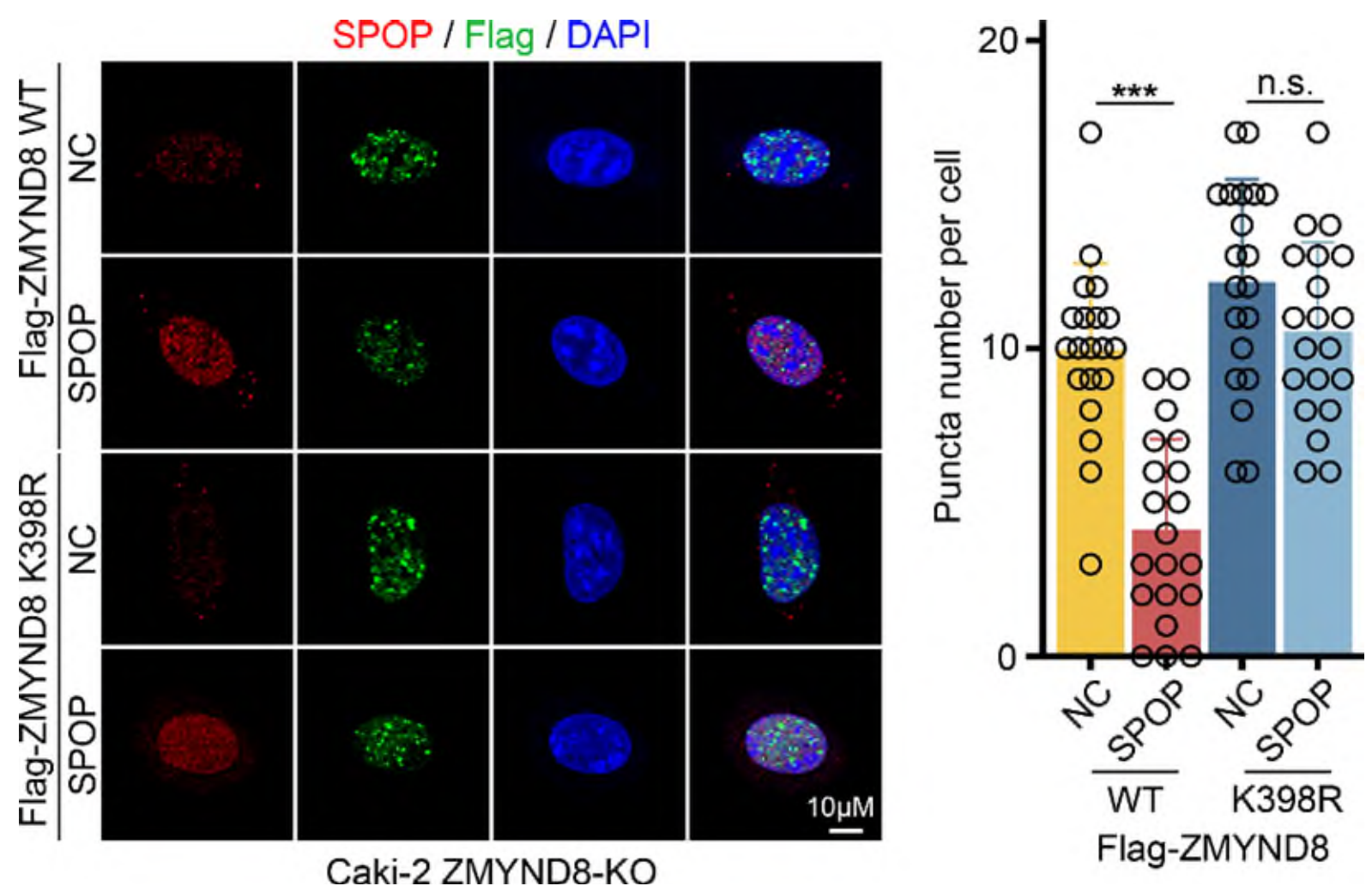

Review Figure 1. (Left) Immunofluorescent cytochemistry (IFC) of SPOP and Flag in Caki-2 ZMYND8-KO cells transfected with indicated plasmids. (Right) Quantification of Flag puncta number in cells from (Left).

Reviewer #6 (Remarks to the Author):

I co-reviewed this manuscript with one of the reviewers who provided the listed reports. This is part of the Nature Communications initiative to facilitate training in peer review and to provide appropriate recognition for Early Career Researchers who co-review manuscripts.

Reply: We thank the Reviewer for reviewing this paper and support of publication of our manuscript in Nature Communications.
